# Combining virtual reality and tactile stimulation to investigate embodied finger-based numerical representations

**DOI:** 10.3389/fpsyg.2023.1119561

**Published:** 2023-04-27

**Authors:** Alyson Matheus de Carvalho Souza, Roberta Barrocas, Martin H. Fischer, Emanuel Arnaud, Korbinian Moeller, César Rennó-Costa

**Affiliations:** ^1^Digital Metropolis Institute, Federal University of Rio Grande do Norte, Natal, Brazil; ^2^Leibniz-Institut für Wissensmedien, Tübingen, Germany; ^3^Department of Psychology, University of Potsdam, Potsdam, Germany; ^4^Centre for Mathematical Cognition, School of Science, Loughborough University, Loughborough, United Kingdom; ^5^LEAD Graduate School and Research Network, University of Tuebingen, Tübingen, Germany

**Keywords:** virtual reality, numerical cognition, finger counting, embodied cognition, cognitive science, virtual environment

## Abstract

Finger-based representation of numbers is a high-level cognitive strategy to assist numerical and arithmetic processing in children and adults. It is unclear whether this paradigm builds on simple perceptual features or comprises several attributes through embodiment. Here we describe the development and initial testing of an experimental setup to study embodiment during a finger-based numerical task using Virtual Reality (VR) and a low-cost tactile stimulator that is easy to build. Using VR allows us to create new ways to study finger-based numerical representation using a virtual hand that can be manipulated in ways our hand cannot, such as decoupling tactile and visual stimuli. The goal is to present a new methodology that can allow researchers to study embodiment through this new approach, maybe shedding new light on the cognitive strategy behind the finger-based representation of numbers. In this case, a critical methodological requirement is delivering precisely targeted sensory stimuli to specific effectors while simultaneously recording their behavior and engaging the participant in a simulated experience. We tested the device’s capability by stimulating users in different experimental configurations. Results indicate that our device delivers reliable tactile stimulation to all fingers of a participant’s hand without losing motion tracking quality during an ongoing task. This is reflected by an accuracy of over 95% in participants detecting stimulation of a single finger or multiple fingers in sequential stimulation as indicated by experiments with sixteen participants. We discuss possible application scenarios, explain how to apply our methodology to study the embodiment of finger-based numerical representations and other high-level cognitive functions, and discuss potential further developments of the device based on the data obtained in our testing.

## 1. Introduction

There is a current surge of interest in our bodies’ constitutive role in our minds’ functioning. This new approach to understanding cognition encompasses various fields of study, including language comprehension, knowledge and decision-making, social coordination, emotional processes, and numerical cognition (for reviews, see Fischer and Coello, 2016; Robinson and Thomas, 2021). In numerical cognition, a significant association between fingers and numbers has been observed repeatedly in studies investigating embodied cognition (Domahs et al., 2010; Barrocas et al., 2019). For instance, on a behavioral level, finger counting habits seem to influence the magnitude processing of Arabic digits (Domahs et al., 2010) and arithmetic performance even in adults (Domahs et al., 2008; Klein et al., 2011). Domahs et al. (2010) showed that when a number is habitually counted across two hands (e.g., number 6 requires extending 5 fingers on one hand and another finger on the other hand) then this incurs an extra time cost when this number is shown in a speeded number comparison task. This was found by comparing the response speed of German and Chinese adults where the latter can show numbers from 6 to 10 on one hand. Domahs et al. (2008) showed that children’s errors in simple arithmetic typically fell close to the correct result and rarely fell farther away. The exception were unusually many errors of plus or minus 5, suggesting that the children kept a hand-based representation. Although, in principle, any configurations of fingers can be used to represent numerical magnitudes, configurations arising from finger counting habits and from displaying quantities seem to establish so-called canonical finger patterns (i.e., culturally prevalent finger postures for depicting numerical quantities, such as thumb, index, and middle fingers for representing 3 in Germany). These canonical finger patterns activate the cognitive representation of number meaning more or less automatically without requiring cognitive effort. Based on this the claim was made that we entertain an embodied representation of numbers that remains systematically coupled to perceptual and motor features of number use (Andres et al., 2008; Moeller et al., 2012). This is supported by behavioral congruency effects and neuroscientific co-activation data.

Two main lines of behavioral research have explored signatures of such embodied finger-based numerical representations in adults. The first line consists of studies reporting sensorimotor influences on adult number processing, such as effects of finger movements on number classification or calculation (Badets et al., 2010; Michaux et al., 2013; Sixtus et al., 2017, 2018). The second line of research is represented by studies investigating how the visual recognition of specific finger patterns facilitates access to mental representations of numbers (Di Luca et al., 2006, 2010; Di Luca and Pesenti, 2008; Barrocas et al., 2019), and how this association is modulated by individual finger counting habits, such as the preference to start counting on either the left or right hand (Fischer, 2008; Lindemann et al., 2011; Wasner et al., 2014, 2015; Hohol et al., 2018).

Although canonical finger patterns do seem to activate numerical representations more or less automatically and without cognitive effort, not much is known about how specific these effects are to embodiment itself rather than being driven by perceptual features of the presented stimuli [see (Berteletti et al., 2021), for an attempt to dissociate these variables]. Recent studies offered insight into that question by showing that the processing of numbers is affected by observing grasping actions only when the hand performing them resembles an actual human hand but not with a robotic gripper (Badets and Pesenti, 2010; Badets et al., 2012; Grade et al., 2017). Therefore, the presentation of human body-related visual stimuli seems necessary for embodied interactions with number processing to occur, although the question lingers as to whether different degrees of embodiment exist, which may produce different degrees of interference with numerical thinking [for a review of graded embodiment, see (Meteyard et al., 2012)]. Why should we stimulate the fingers to study the numerical representation, then? This becomes evident if one assumes embodied representations of numbers where number meaning is systematically coupled to sensory and motor features of number use, such as seeing or producing extended fingers. By stimulating the fingers, we can then examine the predicted presence of behavioral congruency effects.

Testing the interaction between varying degrees of embodiment and numerical reasoning might be the next step in this field of study. One possible way to vary the degree of embodiment someone experiences over fingers or a hand is provided by employing Virtual Reality (VR) to present a virtual hand to the user and to generate different levels of ownership of the virtual hand (Perez-Marcos et al., 2009; Slater et al., 2009). VR is a technology that has been around for some time but has recently attracted the attention of researchers from different disciplines as a new method for researching embodied cognition (Cipresso et al., 2018; Wiepke, 2023). This increase in interest is partly due to new systems coming to the market, making equipment cheaper to acquire and easier to use while still providing realistic VR experiences with a high degree of immersion. Using such VR simulations, scientists can stimulate different senses of the user in a well-controlled environment, thus simulating life-like scenarios with great experimental control (Parsons, 2015).

Besides these methodological advances and advantages, VR can also provide researchers with scenarios that would be impossible to recreate or experiment on in real life. Manipulating the presence or mobility of body parts or changing body representation during an experiment can easily be achieved in virtual scenarios but would be hard to achieve in natural settings (Maselli and Slater, 2013; Kilteni et al., 2015). This versatility of VR allows researchers to decouple, for example, movements of the user’s real hand from movements of their virtual hand presented in the VR environment. Moreover, the virtual hands of a user may move in ways that would not be possible physiologically. Using VR, it is possible, for instance, to have the user raise three fingers of their real hand but see only two fingers raised in VR, creating perceptual conflicts between what participants see and proprioceptively experience in ways that were impossible in real-life experiments before. Implementing such conflict scenarios opens new approaches for understanding embodied cognition in general and the influence of fingers on number processing in particular. To present those scenarios and capture empirical data to evaluate the resulting psychological phenomena, specialized VR software and hardware must be developed and applied.

There are specific requirements for a VR system to be functional in research on finger-based influences on number processing. Tracking and stimulating fingers should be precise about the onset, duration, and location. Additionally, virtual fingers need to respond fast enough to allow continuous interaction without delays so that users perceive them as their own. Lastly, as the VR headset is a wearable device, the VR equipment and the potential stimulation equipment require seamless integration. Considering tactile stimulation of one’s fingers, a Braille cell can produce tactile letters of the Braille alphabet at a user’s fingertips (Holden et al., 2012) and has previously been used to deliver precise tactile stimulation in research on finger-based influences on number processing (e.g., Sixtus et al., 2018). The Braille cell is, however, a relatively inflexible device that constrains the hand of the user into a fixed position, not allowing dynamic tracking of finger movements, as is desirable for VR.

Accordingly, this article describes the development, implementation, and evaluation of a new methodology that uses the Oculus Quest VR headset, integrated with an Arduino-based finger stimulation device, to create a VR experience capable of immersing the user in a virtual environment in which they can receive visual, auditory, and tactile stimulation while seeing their virtual hand moving, because of hand tracking, contingently with their real hand. The Arduino-based device is cheap and simple to build and works well with VR hand tracking, as it is compact and does not interfere with hand and finger movements. Experimenters can present and manipulate different experimental stimuli and the virtual hand in ways that can be both life-like and reality-breaking.

In the following, we will first describe the setup with all parts involved (hardware and software), including information on their construction and relevance to the setup. In the next step, we will present initial tests to validate the devices technically and in a realistic experimental setting. Last, we will discuss our results considering the potential of the methodology for experimental research on finger-based (numerical) representations.

## 2. Materials and equipment

**Figure 1:**
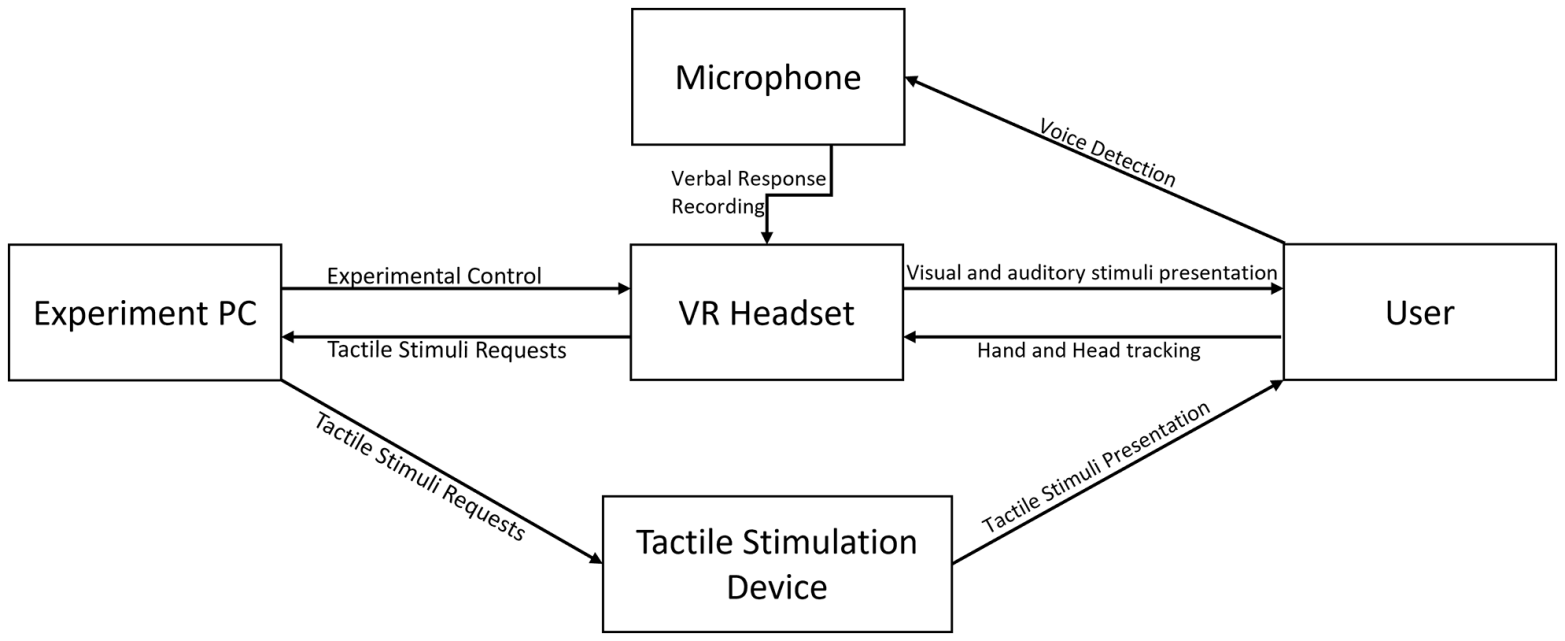
Basic schematic of the four hardware components and how they interact with the user.

The setup consists of four pieces of hardware and three different pieces of software that are integrated to present the user with the virtual environment while also allowing the presentation of an experiment and data collection on the respective experiment. Figure 1 gives an overview of these pieces and how they are connected. In the following, we will describe hardware before software parts of the setup.

First, an *Experiment PC* is responsible for running the control software discussed later in this session. It should be able to execute the game engine Unity smoothly and have a USB port for the Arduino connection. We used a laptop with an Intel Core i7-6,700, a Windows 10 operating system (64 bits), 8 GB of RAM, and an Intel Integrated Graphics Card. Additionally, we developed a *Tactile Stimulation Device* as part of this work, using an Arduino Nano V3 board and five vibration motors, as described in more detail in the methods section. The device is connected to the PC and receives through this connection stimulation requests about which fingers to stimulate and how long to stimulate them for. The VR software generates the requests through the software logic, as described later, and sends them, through a wireless connection, to the PC, which, in turn, sends them to the stimulation device. Supplementary material S1 has an assembly guide for the device and an overview of how to set it up, as described in this methodology. The device is easy to build when following the instructions and uses cheap, readily available parts. It is a core part of this methodology, as it is a more accessible alternative when compared to other hardware used in related work, such as a braille cell. The VR experience is run directly on a *VR Headset*, with all controls and tracking done by the VR software we developed and deployed to the headset as part of this methodology. It is described, alongside the methodology, in the next section. We used an Oculus Quest headset (Oculus Quest, Facebook Technologies, USA; 1,440 × 1,600 resolution per eye, run at 72-Hz refresh rate) because it has built-in hand and head tracking and can present visual and auditory stimulation with sufficient resolution and refresh rate as to increase immersion in the virtual environment. Supplementary material S2 gives a step-by-step guide on setting up this part of the methodology. Lastly, we use the integrated *Microphone* on the Oculus Quest to detect the user’s verbal responses.

On the software side, three pieces integrate all the hardware parts. The first and most important one that will be described in the next section alongside the methodology is the *VR Experience* software, which runs in the Oculus Quest and presents the user with the task that they will be doing inside the virtual world, which is the goal of this methodology. The second part is the *Arduino Software* that will be uploaded into the Tactile Stimulation Device that was developed as part of this work. This software controls the possible stimulation configuration and is made available with a description of how to build the device in Supplementary material S1. The last piece is the *Experiment Control* software. It controls the experiment and connects the VR headset with the tactile stimulation device while recording participants’ answers and storing them in the VR headset. The experiment PC executes the experiment control software written in Unity, which has three different modules. The first one is responsible for the network communication between the PC and the VR headset. It sends and receives UDP packages to/from the VR headset and sends those packages to the control module. The second module is the tactile stimulation device control. This module triggers the previously described patterns and other stimuli the device can provide. The third and central module is the experiment control module which uses the other two modules to manage the experiment and the other equipment involved. The software used in this part is made available with a description of how to install and use it in Supplementary material S2. This software can also record user’s verbal responses, if needed, but is not programmed to do so in this version, as only the detection of the initial verbal response time was relevant to our specific experiment.

## 3. Methods

**Figure 2:**
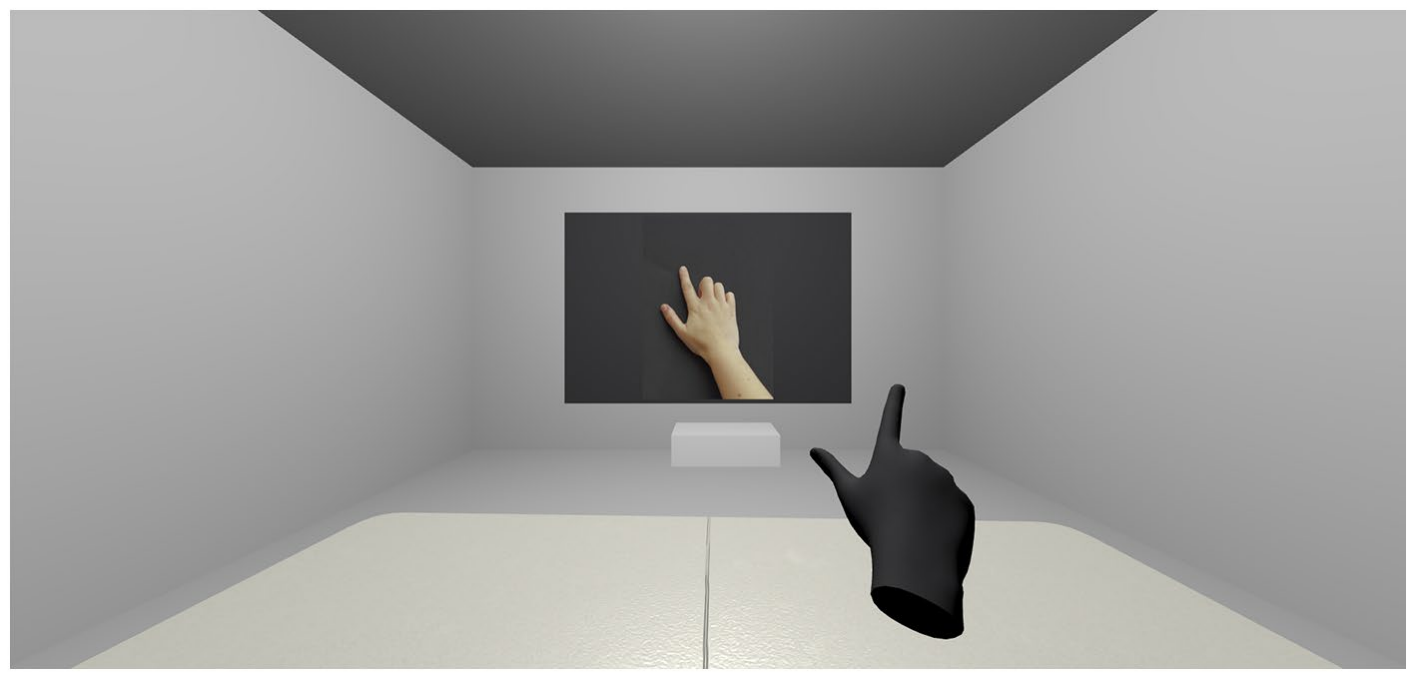
Screen capture of the Virtual Reality experience. The image shows the virtual room the participant is in during the experience. The room has a table, a virtual representation of the participant’s hand (shown in black in the image), and a blackboard that can show different visual stimuli, such as pictures of a real hand (shown in the image) or representations of a virtual hand similar to the participant’s or Arabic digits.

Our methodology consists of a series of trials presented to the user through both the VR environment and the hardware described above. Users are instructed to rest their hands on top of a baseball ball, wait for a tactile stimulus and, when this happens, raise their fingers, making the pattern that was stimulated, which will trigger an image to appear on the virtual blackboard they saw in VR (visible in Figure 2). They should, then, verbally state the number that was on the image. The goal of the method is to detect differences in naming times in case the tactile stimulation either matches or differs from the numerical value shown on the virtual blackboard (*cf.* Sixtus et al., 2017, 2018). Another methodological variation would be to examine such cross-modal integration with different types of visual stimulation on the blackboard, such as Arabic numerals, Roman numerals, non-symbolic dot patterns, images of canonical finger patterns, or non-canonical ones. The tactile stimulation can also be delivered in various ways, such as following a canonical pattern, a non-canonical one, using single finger stimulation, or applying sequential stimulation of the relevant fingers instead of vibrating them simultaneously, as described before.

### 3.1. VR experience and tactile stimulation device development

In the following subsubsections we will describe the development of the tactile stimulation device and the virtual part of the experience, also describing the integration between the parts. Both the tactile device and the virtual experience are described in detail, with their accompanying software made available in the Supplementary materials S1, S2.

#### 3.1.1. Tactile stimulation device

**Figure 3:**
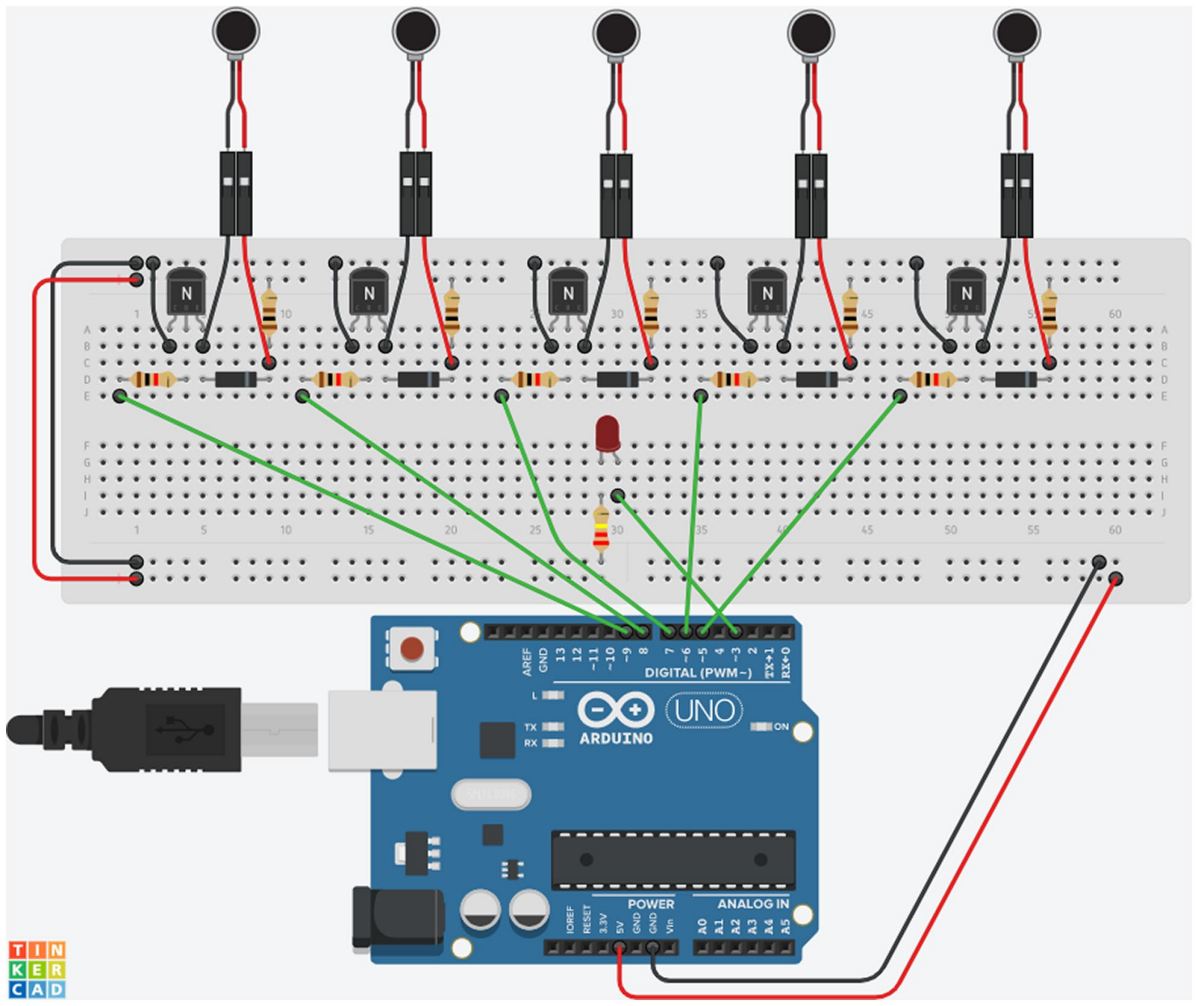
Components design of the Tactile Stimulation Device, using the parts described in Table 1. Each small circuit is composed of one vibration motor (coin-shaped object connected through black and red wires), two resistors, one with 1,000 Ohms (marked with the brown, black, red, and golden lines), and one with 100 Ohms (marked with the brown, black, brown, and golden lines), one Diode (the small black component with a grey line on it), a transistor (the black component marked with an N) and the connecting wires.

The VR experiment is the central part of the present method. However, it relies on the tactile stimulation device to deliver the stimuli quickly and precisely without interfering with the VR rendering. It is important for the results that (i) there is as little delay as possible between the stimulation request and the delivery and (ii) participants experience it adequately. To achieve these objectives, we developed a tactile stimulation device using an Arduino board and a series of electronic components to create a circuit that controls five vibration motors and an LED light, used for control purposes on the system. The list of components is given in Table 1. The design for the circuit can be seen in Figure 3. It is possible to use both the Arduino Uno, as shown in Figure 3, or the Arduino Nano, as described in Table 1. We opted to use the Nano for the final version as it allows for a smaller device overall while also being cheaper. We built two devices with the pieces described in Table 1 so far – the first one in Germany came to a total cost of €45.00 and the second one, in Brazil, cost R$237,39 (Brazilian reais), which was converted to €43.00 at the time, attesting the low-cost of the device.

**Table 1:** Components used in the Tactile stimulation device.

Component	Quantity
Arduino Nano V3 (with USB cable)	1
Breadboard	1
Jumper Wires	15
Jumper Wires (Female to Male)	20
1,000 Ohms Resistor	5
100 Ohms Resistor	5
220 Ohms Resistor	1
LED	1
NPN Transistor PN2222	5
Diode	5
Coin Micro Vibration Motor	5

The device is set up by connecting the Arduino board to the experiment PC through a USB cable that powers the Arduino with 5 volts of current from the PC USB port while allowing the PC to exchange messages with the board. We wrote and deployed a sketch - the Arduino term for an application on the board – that presets the messages the controller PC and the Arduino will exchange during the experiment. The sketch allows us to stimulate all different sets of fingers but also includes the possibility for the motors to be triggered individually through keypresses on the experiment PC and all at once for a short period for testing purposes. We also added in the code the lighting of the LED every time any of the motors is triggered to have visual confirmation of the stimuli.

The device’s final design, shown in Figure 3, is the outcome of several iterations and provides the best results for what we aim to achieve. The main change from the first version was related to the motors. In the first version of the device, we used a different type of vibration motors with a cylindrical shape. These motors used more current than the coin-shaped ones of the final version and were more prone to lockups as these cylindrical motors use a non-symmetrical *head* on the top that turns at a fast speed and, because of its format, makes the whole piece vibrate. This *head* part, though, would frequently get stuck in the hand of the user, failing to start its movement when it was supposed to. This condition would create scenarios where the vibration was delayed or, in some cases, not present at all. For this reason, we decided to use coin-shaped motors, as displayed in Figure 3.

The device is precise, reliable, cheap, and easy to build. To achieve this, we used widely available components and assembled them in a way that should be easy to replicate and require little to no knowledge of electronics. The use of a breadboard to connect the components enables the device to be built without any soldering on the board itself, using just the jumper wires to connect the pieces.

**Figure 4:**
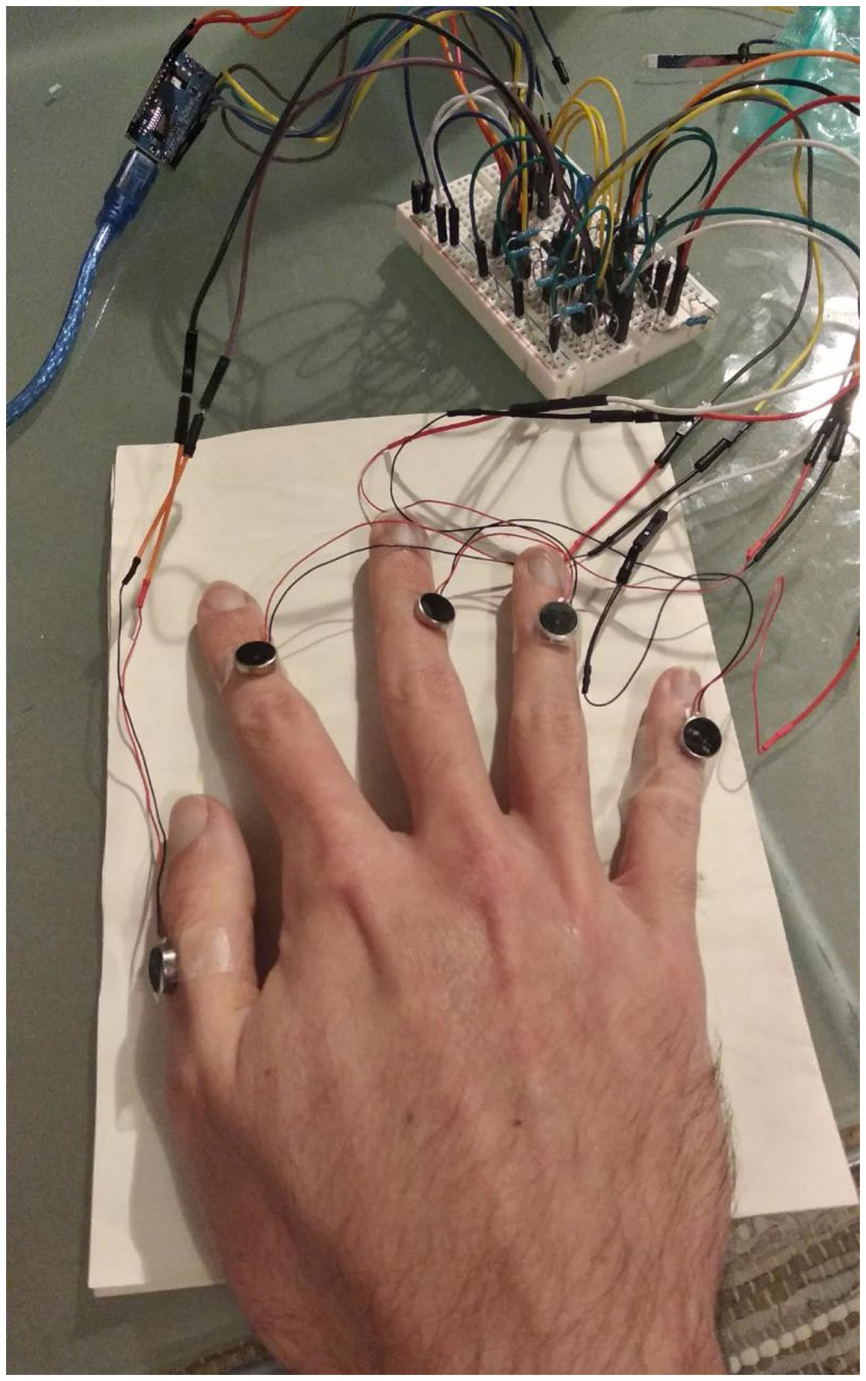
Final version of the Tactile Stimulation Device as applied to the hand of a participant.

The circuit comprises five replications of the same small circuitry, one for each vibration motor, as seen in Figure 3, plus the LED indicating the powering on and off of the motors. Each small circuit is composed of one vibration motor, that is the actuator of the circuit, two resistors, one with 1,000 Ohms (marked with the brown, black, red, and golden lines), and one with 100 Ohms (marked with the brown, black, brown, and golden lines), one Diode (the small black component with a grey line on it), a transistor (the black component marked with an N) and the connecting jumper and Dupont wires. The Arduino is the controller for the whole circuit. The breadboard (the whiteboard with the holes where everything is connected, shown in Figure 3) spreads the current necessary to power the circuit, taken from the Arduino. A detailed description with pictures of how to assemble the device can be found in Supplementary material S1. The final version of the device and how it is applied to the participant’s hand can be seen in Figure 4.

#### 3.1.2. VR experience

The central part of the setup is the VR experience. It is presented to the user *via* the Oculus Quest VR headset. The experience consists of a grey room with a table and a blackboard in front of the user. The blackboard is used to present stimuli (here, images of finger patterns and Arabic numbers, as often occurs in real-world finger-based experiments). This setup allows us to compare results to real-world experiments if needed. The virtual environment also contains a virtual representation of the user’s hand (see Figure 2, in black), animated by movements of the user’s real hand through the built-in hand tracking system of the Oculus Quest. This animation is the most important part of the setup, as it allows to manipulate this virtual hand to realize effects that would not be possible in a real-life setting.

Previous studies show that it is possible and somewhat easy to induce a sense of ownership over a virtual limb when it is tracked and adequately represented (Slater et al., 2009; Maselli and Slater, 2013; Kilteni et al., 2015). This is also the case in our virtual hand setup.

To this basic setup, we added the possibility to manipulate which real finger controls which virtual finger. This feature is a main advantage of our methodology, as such possibility allows for creating visual misrepresentations of gestures performed by the participant. In turn, this also generates the possibility of decouple proprioceptive sensation from visual perception by misrepresenting the lifting of the virtual fingers. For example, we may couple the real index finger with the virtual index finger and the virtual pinky. Thus, when the participant raises the thumb and index finger of their real hand, in a gesture as the one displayed in Figure 2, the virtual hand will, instead of repeating the gesture, raise the thumb (as it is mapped to itself) as well as index finger and pinky. Such manipulations can create different degrees of conflict between the proprioceptive sensation the participant is experiencing by raising two fingers and the visual perception, where they see more (or fewer, or different) fingers raised on their virtual hand.

To achieve this conflict, we use the information on the angles of the finger joints (raised or bent) and the fingers’ current position relative to the palm. We can translate the movement to a different finger without distorting the target finger, changing its size, or placing it in the wrong location. The method creates a realistic representation of the “mirrored” finger and allows the display of hand gestures on the virtual hand more organically and fluidly. However, the method does not work for the thumb that is positioned in a different orientation and has a different range of motion compared to the other four fingers. We, therefore, decided to always keep the thumb as itself in the virtual hand representation. The other four fingers, however, could be interchanged, used to represent multiple fingers, or just be ignored (making the coupled virtual finger immobile even when the real one was moved).

**Figure 5:**
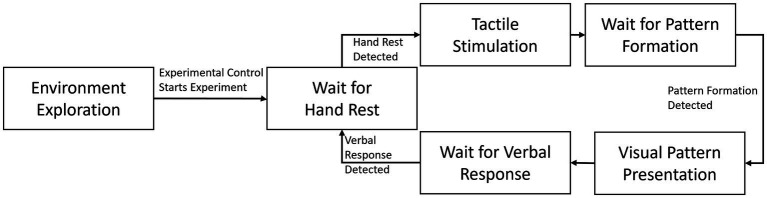
Experimental loop used in the example experiment.

Besides the virtual hand implementation and the blackboard with the stimuli presented, the VR interface contains all the code necessary to run the experiment and control the tactile stimulation device. As mentioned above, the experimental control comes from the experiment PC, through a common network connection between the VR headset and the PC. Everything runs directly on the headset to guarantee a smoother VR experience, with very little delay (we estimate it around 5 ms but could not measure it as it was so small) and precise input/output timing. The general experimental loop of our methodology is depicted in Figure 5 and described in detail in the next subsection.

### 3.2. Experimental methodology

The first part of the experiment is just for the individual user to get familiar with the virtual environment. The participant can look around, move their hands, and get a feeling for the virtual room. After this initial immersive experience, the critical experiment is started by a keypress on the experiment PC. The blackboard shows instructions to the user, first asking them to raise their dominant hand from a resting position on a table. This step helps the system to calibrate the height at which the participant usually lifts their hands while also setting up the dominant hand that will be used for the rest of the VR experience. After this calibration step, the critical trials begin. The trials are based on a predetermined stimulus set uploaded to the system by the experimenters beforehand. This list contains information like trial order, the finger pattern that will be shown in each trial, the type of visual input presented (finger images, Arabic numbers, virtual models, or the participant’s virtual hand), and both spatial and temporal details of the pattern that will be stimulated on the users’ hands for each trial, using the tactile stimulation device.

A trial starts with the participant resting their hand on a baseball placed on the table. The ball forces a closed hand instead of an open one at the start of each trial. As a result, when asked to generate a specific finger pattern with their hand in the next step, the participant raises the correct number of fingers instead of bending the ones unnecessary. The system automatically detects the resting pose, which triggers the second step of the trial – the tactile stimulation.

The tactile stimulation device can stimulate the users’ fingertips through vibration motors, allowing the experimenter to stimulate different finger patterns without the need for verbal or visual communication of those patterns. After the stimulation, the user raises their dominant hand to produce the stimulated pattern of fingers. This method is highly intuitive in that responding with the stimulated fingers is a compatible response that bypasses the time cost of response selection, according to which response latencies normally increase with the number of stimuli to select from Leonard (1959).

The system again detects the respective pose and exhibits, with the appropriate timing, the visual number in one of the formats mentioned above, either on the blackboard or directly on the users’ virtual hands, using finger swapping and mirroring technique as described above, when intended. After the visual stimulation has occurred, the system waits for the user to state the observed pattern verbally and, as soon as the microphone detects the verbal response onset, it goes back to the initial state, waiting for the participant to rest their hand once again on the baseball so a new trial can begin.

Depending on the use of our methodology, it is also possible to add an additional step to the end of this process, namely, to manually record the verbal response on the keyboard of the experiment PC. If this is desired (e.g., to facilitate later data analysis), the system waits for the respective keypress to ensure that the experimenter recorded the participant’s response before moving on. After all defined trials from the stimulus set were presented, the blackboard displays a message thanking the user for their participation and instructing the removal of the VR apparatus.

The system also generates, after the experiment, a data table, which includes a participant ID, the start time of the experiment, the time it took for the experiment to load and then, for each trial: trial number, item characteristics such as the tactile pattern that was stimulated in the example experiment, the onset time of the stimulation, the time it took for the participant to produce the stimulated pattern, the visual stimuli that was displayed, the onset time of the visual stimulus and the onset time of the verbal response regarding the displayed pattern. If configured accordingly, the system also records the verbal response coded by the experimenter. All responses are measured to the nearest millisecond but stay within a single frame window: as the experiment runs at 72 frames per second (FPS) (matching the refresh rate of the VR headset) the recording occurs once every 13.8 milliseconds. After the experiment is concluded, a .txt file is generated with the participant ID as its name and then saved to the headset. It can later be extracted *via* USB connection for analysis.

### 3.3. Validation of equipment

**Figure 6:**
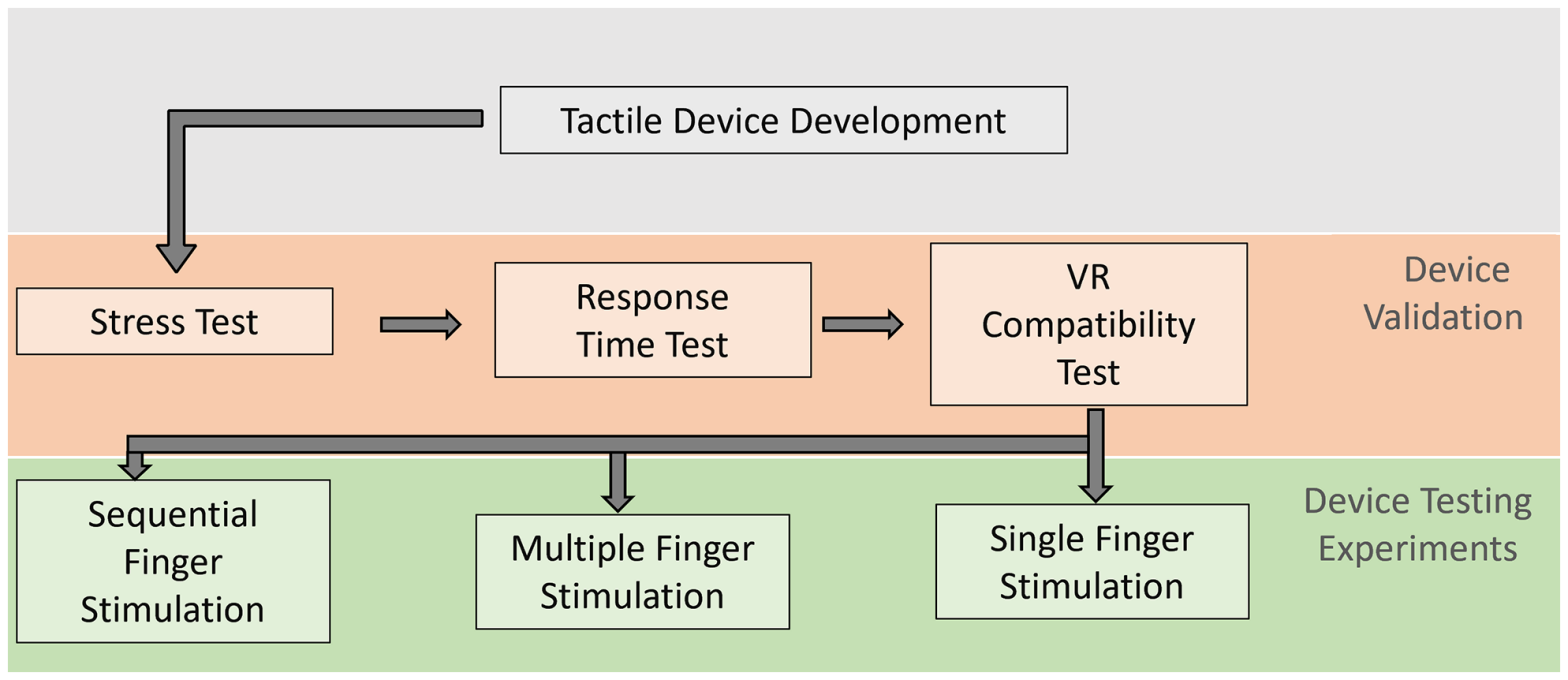
Flowchart illustrating the evaluations steps. Following the development of the tactile stimulation device, it was validated in three tests on the usability of the device itself. After it proved functionally valid, three empirical experiments tested the participants recognition of finger stimulations, as described in Section “Validation of Equipment.”

The evaluation of the tactile stimulation device was performed in four steps. The first step was to stress-test whether the system was reliable and capable of delivering the stimuli without interruptions or failures, aggregating confidence in the data. The second step was to test the response time of the system. As the VR headset controlled the device through the experiment PC, we wanted to evaluate whether this added any extra delay between the controller and the device, which could alter the results of the experiment. The third test addressed the compatibility of the device with the VR headset. As our apparatus adds extra bits to the participant’s hand, this test validated that the hand could still be tracked well enough even with the device present. Lastly, we needed to evaluate whether the device adequately delivered the stimuli. To do so, we tested participants’ ability to recognize which fingers were being stimulated. These tests are described in the following subsubsections Figure 6 illustrates the steps taken for a technical validation of the device in a first and experimental testing of its stimulation capacity in a second step.

#### 3.3.1. Tactile stimulation device stress test

The first validation procedure was the stress test to evaluate the device’s reliability. This test was performed on the device itself and involved no participants.

##### 3.3.1.1. Procedure

The stress test aimed to evaluate whether the system was reliable in delivering the stimuli and capable of working for a long period of time without interruptions. To do so, we connected the device to a PC that would later be used as the experiment PC and then connected both the PC and the VR headset to the same wi-fi network. We did this to mimic the experimental setup and have the VR headset control the device as would happen in a proper experiment.

After setting up the devices, we uploaded a code to the VR headset that would message the experiment PC to trigger all the motors in the stimulation device once every second for a duration of 200 milliseconds and then turn them off again. This code would repeat indefinitely until manually stopped. The motors were then placed on top of a hard surface (a table with a glass top) and recorded using a 60 FPS cell phone camera.

##### 3.3.1.2. Experiment and measures

The experiment consisted of running the vibration code for 2 hours, with the motors vibrating every second for 200 milliseconds and turning off. The goal was to measure if each of the motors would activate and deactivate every time. After 2 hours passed, the recording and the experiment were stopped, and the video was manually evaluated in search of any absence of activation of the motors. As the timings were predetermined and the LED light would also indicate that the motors should be firing at that moment, it was possible to precisely evaluate whether any of the motors failed to activate during any of the activation requests.

#### 3.3.2. Tactile stimulation device response time test

After its validation regarding reliability on the stress test, the second test done with the prototype targeted the system’s response time. The goal was to measure the delay between the stimuli request from the VR headset and stimuli delivery (i.e., vibrations) delivered by the device. When not accounted for, this time would be considered extra time for the user to react. This test was performed on the device by the experimenters and required no participants.

##### 3.3.2.1. Procedure

The experimental setup was assembled again, using the experiment PC and the VR headset, connected to the same wi-fi network, and the tactile stimulation device connected to the experiment PC, as described above. From there, we ran a test application similar to the one used in the stress test that would turn on all the motors and the LED every 2 seconds, keeping them vibrating for 200 milliseconds. This application would also make the screen on the VR headset entirely white when the stimuli started and fully black when they ended, as this would create a bright light coming out of the headset when it was asking for the stimuli.

We then placed the VR headset and the tactile stimulation device close to each other to capture both devices in the same recording of a 60 FPS camera (the same one used in the stress test). After that, we ran the application on the VR headset and filmed the response time test using the camera. The LED light of the tactile stimulation device would indicate that the stimulus was being delivered while the sound made by the vibration motors could also be detected in the footage. The lighting of the VR headset screen would also be visible in the frame.

##### 3.3.2.2. Experiment and measures

The experiment consisted of recording the setup vibrating the motors and lightning the VR device for 5 minutes. After that, we took the footage to a video processing software to evaluate, frame by frame, when the stimulation was requested and when it was delivered. We also considered the sound made by the vibration motors to see if there was any difference in delivery time between the lighting of the LED and the start of the motors. We then measured the time between the lighting up of the VR screen (indicating that a signal was sent), the lighting of the tactile stimulation device LED (indicating that the signal was received), and the beginning of the vibration of the motors.

#### 3.3.3. VR compatibility test

After validating the tactile stimulation device hardware and its integration with the VR headset as a controller, the next step was to validate whether it could be used with the built-in hand tracking that the Oculus Quest provides.

##### 3.3.3.1. Participants

For this test, we used two different participants: a male participant (aged 31) with a larger hand (25.5 cm hand span from the tip of the thumb to the tip of the pinky) and a female participant (aged 34) with a smaller hand (20.8 cm hand span).

##### 3.3.3.2. Procedure

We ran a few tests using the device in different positions applied to the hand of the user to see how this would affect the Oculus’ hand tracking. We tried using the motors placed on the dorsal part of the hand in the region of the middle phalanges and on the distal phalanges and tried the motors in the palmar part, in the region of the distal phalanges. At each position, we tested whether there was any loss of tracking during movements of the hands into poses like those expected during behavioral experiments (when producing both canonical and non-canonical finger patterns).

##### 3.3.3.3. Experiment and measures

We tested each of the placements of the motors in the two participants and measured if there was any loss of track of the hand during the experience. In all three cases for the motor positioning, we started the participants in the resting hand position and asked them to do one of the hand signals that were expected during the real experiment. We repeated that for each of the signals and measured the virtual hand movement in the VR environment.

#### 3.3.4. Device accuracy test—Single finger stimuli detection

After conducting all the technical tests on the equipment successfully, the next step was to test the interaction of the device with a participant because delivering the adequate experience of the vibrotactile stimulation is the main goal of the device. The first test conducted was intended to validate whether participants could identify single-fingers being stimulated by the device. They wore the device as they would normally do during an experiment and then had to answer verbally about single finger stimuli, which we presented using software similar to the experiment control described before.

##### 3.3.4.1. Participants

For this test and each of the following tests, we conducted sixteen replications of the experiment with the same eight male and eight female volunteers (aged between 31 and 66 years). All participants reported no relevant discomforts during or after the experiment, through a self-report questionnaire. The tests used a different order for each participant to minimize the effects of learning and tiredness on the outcome.

##### 3.3.4.2. Procedure

For the experiment, participants would come to the lab and give their informed consent to participate voluntarily in the experiment. Afterward, participants would be invited to sit in a height-adjustable office chair. The chair would then be adjusted so that the participant’s dominant arm would rest comfortably on the table in front of them. Next, we cut small pieces of a finger sleeve made of elastic fabric lined with silicone gel, one for each finger, and placed them on the fingers of the participant, over their fingernails. After that, we attached the motors to this piece of fabric on each finger, using a hook-and-loop fastener. The wires of each motor were secured away from each other to not interfere with their vibration. Then each motor was triggered once to check whether they were working properly and whether the participant felt their vibration.

Afterward, participants started with their respective experimental conditions following a Balanced Latin Square design on the order of the experiments between this one and the next two that will be presented. A Balanced Latin Square design was employed to counterbalance the order of conditions across participants to minimize effects of learning, tiredness, or experience, in being the first or last in a series of tests, for example. The experimenter would then set the parameters corresponding to each condition and instruct participants on the experimental procedure. Lastly, participants would be asked to state when they were ready to start. The participants had a full view of their hand with the equipment during the whole experiment, with no soundproofing. After each condition, a one-minute break was allowed while the experimenter checked the data files and their validity. After that, the second condition would start. This procedure would repeat until the last condition was finished.

##### 3.3.4.3. Experiment

Participants had to detect 60 stimulations, 10 on each finger and 10 on all fingers. Stimulus order was random but with the constraint that no finger was stimulated twice in a row. After each stimulation, participants were asked to raise the finger that was stimulated and state its name. They were also told that, if multiple fingers were stimulated, they should state all of them. After participants raised and named the finger(s) during each trial, the experimenter would type in the response(s), the software would register it and move on to the next stimulus. After all stimuli were presented, a text file was saved with the experiment data for later statistical analysis.

##### 3.3.4.4. Measures

For each participant, we wrote down the fingers raised by the participant, indicating that they were stimulated and the correct answer generated by the software. We then compared the data to see the number of correct answers by each participant.

##### 3.3.4.5. Statistical analysis

To analyze the sample statistically, we used a binomial test based on the percentage of correct answers for each participant. The test was applied to test the following hypothesis:

- Null hypothesis: the probability of answering correctly is equal to 95% ( $H_{0}:p=0.95$ )
- Alternative hypothesis: the probability of answering correctly is different than 95% ( $H_{1}:p\neq 0.95$ )

This 95% threshold value was chosen based on the observation of the mean success rate in this experimental case and was confirmed, using the binomial test, to be a value that would be close enough to what was observed in the results, but did not reject the null hypothesis.

#### 3.3.5. Device accuracy test—Gesture simultaneously stimuli detection

The second test was done to test the precision of the device when stimulating multiple fingers simultaneously, as it would happen in a situation where stimulating a certain combination of fingers is relevant.

##### 3.3.5.1. Procedure

Participants were again asked to raise the fingers that were stimulated after each stimulation. After the finger pattern was generated, the experimenter would then type in the respective number generated and whether it was a canonical or non-canonical. The procedure for this experiment was identical to the single finger stimulation test mentioned above, except that the stimulation happened for 500 ms and for all respective fingers simultaneously. We had six different finger patterns for this test – canonical two (thumb and index), canonical three (thumb, index, and middle finger), canonical four (thumb, index, middle and ring finger), non-canonical two (thumb and pinky finger), non-canonical three (thumb, index, and pinky finger) and non-canonical four (thumb, middle, ring, and pinky finger).

We also added a device check in the experiment to make sure we could detect any problem during the experiment. This check consisted of a 200 ms stimulation of each finger individually, from thumb to pinky, to verify that all the motors were working, and the participant was experiencing their vibration adequately, guaranteeing that we had no problems in the device and no disconnects of the motors. This test always succeeded during all the experiments, indicating no problem with the device or motors. The experimenter was able to observe the motors as they vibrated to confirm with the participant that they were all working.

##### 3.3.5.2. Experiment

As in the single finger stimulation test, the participant would receive 60 stimuli, 10 for each of the poses mentioned before in random order with the constraint that no finger pattern would be stimulated twice in a row. At the beginning of the experiment, participants were instructed to raise their fingers after the stimuli to show the pattern that they felt was stimulated. The experimenter would then write down in the software the pattern that was raised, and that was saved in a table with the correct one for later analysis.

##### 3.3.5.3. Measures

For each participant, we saved the gesture that was reported by the participant through lifting the fingers they felt were stimulated and the correct answer, generated by the software. We then compared the data to see the number of correct answers by each participant.

##### 3.3.5.4. Statistical analysis

To analyze the sample statistically, as in the single finger stimulation test, we used a binomial test based on the percentage of correct answers for each participant. The test was applied to test the following hypothesis:

- Null hypothesis: the probability of answering correctly is equal to 75% ( $H_{0}:p=0.75$ )
- Alternative hypothesis: the probability of answering correctly is different than 75% ( $H_{1}:p\neq 0.75$ )

#### 3.3.6. Device accuracy test—Gesture sequential stimuli detection

The third test was done to evaluate whether participants would have an easier time detecting the multiple finger stimulation when it happened sequentially instead of all fingers simultaneously. This is a different condition that can also be useful in some finger-counting experiments. This test was picked up after feedback from some colleagues who tested the device that the sensation became blurry when multiple fingers vibrated at the same time, which was not reported when the stimulation happened sequentially.

##### 3.3.6.1. Procedure

For this test, we had the same six different finger patterns as in the previous test. The stimulation would always happen from thumb to pinky in each gesture and the duration of the stimuli was 500 ms, as in the other conditions, but a 200 ms delay between the end of the one stimulus and the start of the next one was added. For example, when the canonical number three was to be stimulated, the thumb would vibrate for 500 ms, followed by 200 ms of no vibration, then the index would vibrate for 500 ms, then 200 ms of no vibration and lastly the middle finger would vibrate for 500 ms, concluding the sequence for the canonical number three.

We also added the same device check as mentioned in the last experiment to make sure we could detect any problem during the experiment. This test succeeded in all the experiments, indicating no problem with the device or motors. The experimenter was able to observe the motors as they vibrated to confirm with the participant that they were all working.

Participants were again asked to raise the fingers that were stimulated but this time only after the stimulation had ended. After the finger pattern was generated, the experimenter would then type in the respective number generated and whether it was a canonical or non-canonical. The rest of the procedure for this experiment was identical to the single finger stimulation one mentioned above.

##### 3.3.6.2. Experiment

As this test was a lot longer because of the delays between each stimuli and also the duration of each single finger vibration, the participants received only 30 stimuli, 5 for each of the poses mentioned before still in random order and with the same constraint that no finger pattern would be stimulated twice in a row. As mentioned before, this time they were instructed to only raise the stimulated fingers after the whole stimulation had ended, and all at once. The experimenter would then write down in the software the pattern that was raised and that was saved in a table with the correct one for later analysis.

##### 3.3.6.3. Measures

As before, for each participant, we saved the gesture that was reported by the participant through the lifting of stimulated fingers and the correct answer, generated by the software. We then compared the data to see the number of correct answers for each participant.

##### 3.3.6.4. Statistical analysis

To analyze the sample statistically, we used a binomial test based on the percentage of correct answers for each participant. The test was applied to test the following hypothesis:

- Null hypothesis: the probability of answering correctly is equal to 92.5% ( $H_{0}:p=0.925$ )
- Alternative hypothesis: the probability of answering correctly is different than 92.5% ( $H_{1}:p\neq 0.925$ )

### 3.4. Ethics committee

The studies involving human participants were reviewed and approved by the Ethics Committee of the Leibniz-Institut für Wissensmedien. The patients/participants provided their written informed consent to participate in this study.

## 4. Results

### 4.1. Tactile stimulation device stress test

The second version of the device (with the coin-shaped motors) was tested in the setup described before. This test was interrupted before the end of the first 10 minutes, as it was detected that some motors were not firing when they were supposed to. After some investigation, it was discovered that this was related to the connection between the motor and the female to male jumper wires that was used to connect it to the breadboard. As the wire in the motor is very thin, it would, with the vibration, detach from the connecting wire, thus breaking the circuit. After some vibration from the other motors, though, the cable would randomly slide back in place and the motor would be back to working again. This fault was corrected in the new version, shown in Figure 4.

**Figure 7:**
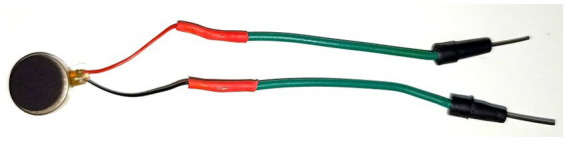
Soldered connection.

To avoid those random disconnections, the motors’ thin wires were soldered into some jumper wires and braced with thermal bracers, as shown in Figure 7. This modification stabilized the connection of the jumper wires to the breadboard and allowed for the motors to vibrate without damaging the connections. After this last change, the test run with the final version of the tactile stimulation device was started. The test ran for 2 hours, as planned, and the device was intact throughout the experiment. Reviewing the video indicated that the device showed no signs of failure, and the motors were able to deliver all the stimulation that they were requested to.

### 4.2. Tactile stimulation device response time test

In a 60 FPS camera, there is one frame in about every 16.7 milliseconds. After moving through the frames in the video footage of the experiment, we detected that both the white screen of the VR device and the LED would always light up in the same frame, indicating that the delay between the stimulation request and the delivery would not be longer than 16.7 milliseconds in the setup we are using. In the next frame, the vibration motors would start moving. We attribute this delay of, at maximum, 16.7 milliseconds for the movement of the motors to be the startup time that the motor has, as the LED was indicating that the request was almost instantaneous, and the motor needs to rotate slightly to generate the vibration before it is noticeable. Another important observation was that this interval was constant throughout the experiment. So, even if this small delay is not ideal, it can be considered irrelevant when defining the timings in the experiment. Not once, during the five-minute trial – totaling 150 stimuli requests - the motors took more than one frame (16.7 milliseconds) to start.

### 4.3. VR compatibility test

In all the three conditions assessed with these two participants, the Oculus Quest built-in hand tracking worked as expected, with no extra losses of tracking when compared to the same movements done without the motors attached to the hand. As described in the VR Experiment section, the system was able to recognize the hand rest position and the pattern formation trigger without problems in all device positions. This successful recognition of hand poses was expected because the cables on the motors are very thin (less than 1 mm), and the motors themselves are also small (1 cm diameter).

### 4.4. Device accuracy test—Single finger stimuli detection

The results indicated that the success rate of the sample was 96.56%, with a standard deviation of 4.41%. The binomial test resulted in a value of *p* of 0.0858757, meaning that it’s not possible to reject the null hypothesis or, in other words, there is not enough statistical evidence to say that the probability of answering correctly is different than 95% for this experiment. This 95% threshold value was chosen based on the observation of the mean success rate in this experimental case and was confirmed, using the binomial test, to be a value that would be close enough to what was observed in the results, which had a mean of 96.56%, but did not reject the null hypothesis. For each experiment, we tested values from 70 to 100% to define which would work as the threshold. The same was done on the other experiments, always observing the results from what was obtained in the experiment. This guided us on the expected accuracy of each method of stimulation, based on the sample we had.

### 4.5. Device accuracy test—Gesture simultaneously stimuli detection

The results observed indicated that the success rate of the sample was 84.38%. The standard deviation of the sample was 11.25%. The binomial test resulted in a value of *p* of 0.0542599, meaning that it’s not possible to reject the null hypothesis or, in other words, there is not enough statistical evidence to say that the probability of answering correctly is different than 75% for this experiment, following the same method as the last experiment.

### 4.6. Device accuracy test—Gesture sequential stimuli detection

The results indicated a success rate of 98.54% with a standard deviation of 2.45%. The binomial test resulted in a value of *p* of 0.0554578, meaning that it’s not possible to reject the null hypothesis or, in other words, there is not enough statistical evidence to say that the probability of answering correctly is different than 92.5% for this experiment.

## 5. Discussion

The present study developed and evaluated a new method for combining tactile stimulation with VR to allow new avenues for research into embodied representations, presented in the example of finger-based numerical representations (see “Introduction”). By creating a VR environment where we can manipulate the virtual hand of the user and create mental conflicts regarding the representation of the virtual hand and the perception of the real one, it is possible to evaluate new behaviors from the participants based on those new possibilities. This experimental possibility is especially relevant to finger-based embodied cognition research as this can help further understand how the mental representation of numbers happens based on the hand signals we use to represent them. The device built as part of this research was tested on its technical reliability and the practicality of its use. Reliability tests were done with a large number of stimulations to validate that the parts of the device were working and would do so even in a longer experimental setting. Those settings included a stress test of the parts, a device response time test, and a VR compatibility test to validate whether the device could be used with the standard Oculus Quest hand tracking. The device successfully withstood a two-hour constant use stress test, showed a response time of no more than 16.7 milliseconds for the initial stimulation to take place, and proved compatible with the VR headset hand tracking, indicating that the device works adequately and may well be used with human participants.

We then proceeded to test the device for the practicality of its use with 16 participants on three different conditions. The first experiment evaluated whether participants were able to detect single finger stimulations adequately, whereas the second one appraised the detection of stimulating multiple fingers at the same time. The third experiment tested whether participants could detect stimulation of multiple fingers in a sequential pattern better than during simultaneous stimulation. The devices always vibrated at the same frequency and intensity and were placed at the same site on the participant’s fingers.

The duration and the frequency of stimulation can affect vibration perception (Ekman et al., 1969) and the placement of the motors may also affect perception (Sofia and Jones, 2013), with the hand being the most sensitive part and especially the fingers (Verrillo, 1971). With a shorter vibration duration of around 500 ms and with the used coin motors’ vibration frequency, the stimulation seems to feel more concentrated as it does not continue long enough to disperse through the skin from the fingers into the palm.

In the first and last conditions, mean response accuracy (i.e., correctly detected stimulation of 1 or multiple fingers) was 96.5 and 98.5%, respectively, suggesting that the developed device can effectively deliver tactile stimulation to participants in those cases. The second condition generated confusion among the participants, especially when only a single finger was not stimulated, with about 75% of correctly recognized stimulations.

An aspect to consider in this context is that the developed device cannot change the intensity nor the frequency of the vibrating stimulation. There might be other options to manipulate tactile sensations instead of changing stimulus duration. Developing a device with the capacity for intensity manipulations goes beyond the scope of the present study, which aimed at establishing the validity and practicality of an easy-to-build device for tactile stimulation of fingers and its integration in a VR environment as described exemplarily for research on embodied finger-based (numerical) representations.

It is also possible to assemble the device slightly differently, exchanging the vibration motors for step motors and adding their corresponding drivers to create a slightly more complex device that can deliver different tactile stimulation. Vibration motors are vibrotactile devices and lead to a different sensation when compared to pressure perception (Dargahi and Najarian, 2004). It would be possible to develop something closer to a Braille cell using stepper motors while keeping a lower cost. The device would be a single point pressure device instead of the multiple point matrix the Braille cell provides. These modifications would allow experimenters to try and explore different types of stimulation at lower cost and with a smaller profile compared to devices that have already been applied in the literature (Sixtus et al., 2018). This alternative is a potential future avenue for the newly developed stimulation device described in this article.

The described tactile stimulation device and its integration with the VR system (with parts mentioned in Figure 1) presents a novel methodology for research on embodied cognition in general and finger-based (numerical) representations. The developed device is low-cost and straightforward enough to be assembled with almost no prior knowledge of electronics anywhere in the globe. Additionally, the costs of VR devices are getting lower, so the described combination should enhance possibilities for experimentation in this field of research. This approach can lead to a better understanding of the involvement of bodily experiences in human cognition. The combination of tactile stimulation and VR can also contribute to our understanding of the role of bodily sensations in the immersive experience of VR environments.

## Data availability statement

The raw data supporting the conclusions of this article will be made available by the authors, without undue reservation.

## Ethics statement

The studies involving human participants were reviewed and approved by Ethics Committee of the Leibniz-Institut für Wissensmedien. The patients/participants provided their written informed consent to participate in this study. Written informed consent was obtained from the individual(s) for the publication of any potentially identifiable images or data included in this article.

## Author contributions

AS, KM, RB, and MF contributed to the conception and design of the methodology. AS and CR-C designed the tactile device. AS developed the hardware and software used in the methodology. AS and EA designed and executed the experiments, collected, and analyzed data related to the testing of the hardware. AS and RB wrote the first draft of the manuscript. AS, RB, MF, EA, KM, and CR-C wrote sections of the manuscript. All authors contributed to manuscript revision, read, and approved the submitted version.

## Funding

This study was financed in part by the Coordenação de Aperfeiçoamento de Pessoal de Nível Superior – Brasil (Capes) – Finance Code 001. MF was partially supported by DFG grant FI 1915/5-2 and publication costs were covered by the University of Potsdam under sponsorship from the Deutsche Forschungsgemeinschaft. The study was supported by NPAD/UFRN. Part of this research was funded by Deutsche Forschungsgemeinschaft project number MO 2525/7-1 to KM and Stephanie Roesch. This study was supported in part by the Royal Society - Newton Advanced Fellowship R2/202209 to CR-C.

## Conflict of interest

The authors declare that the research was conducted in the absence of any commercial or financial relationships that could be construed as a potential conflict of interest.

## Publisher’s note

All claims expressed in this article are solely those of the authors and do not necessarily represent those of their affiliated organizations, or those of the publisher, the editors and the reviewers. Any product that may be evaluated in this article, or claim that may be made by its manufacturer, is not guaranteed or endorsed by the publisher.
