# Supplementary material for: Combining virtual reality and tactile stimulation to investigate embodied finger-based numerical representations
Source: Front Psychol. 2023 Apr 27;14:1119561. doi: 10.3389/fpsyg.2023.1119561 (PMC10174462; doi:10.3389/fpsyg.2023.1119561)

Combining virtual reality and tactile stimulation to investigate embodied finger-based numerical representations

Supplementary Material 1

Tactile Stimulation Device

Equipment, Components and Tools

Component	Quantity
Arduino Nano V3 (with USB cable)	1
Breadboard	1
Jumper Wires	15
Jumper Wires (Female to Male)	20
1000 Ohms Resistor	5
100 Ohms Resistor	5
220 Ohms Resistor	1
LED	1
NPN Transistor PN2222	5
Diode	5
Coin Micro Vibration Motor	5
Thermo-retractable Tube for the Motor Wires*	10

Tools	Quantity
Soldering Iron	1
Pliers	1

Equipment	Quantity
Experiment PC	1
Arduino USB Cable	1

* Highly recommended but not necessary

Tactile Stimulation Device

- The Tactile Stimulation Device is controlled by the Experiment PC, running the Experiment Controller application in Unity.

- In order to build and test the device in parts, we will first describe how to set up the Experiment PC application in Unity and then elaborate on how to build the Tactile Stimulation Device so it can be tested with the application step-by-step.

- All the necessary files and diagrams will be made available in their latest version at https://github.com/AlysonSouza/FingerCountingVR

Setting up the Experiment PC

- Download and install Unity Hub from the link: https://unity.com/download

- In Unity Hub, create a Unity account and/or login. Close the welcome windows and go to the *Installs* tab in the hub. Click Install Editor and download and install Unity version 2020.3 with **Microsoft Visual Studio, Android Build Support, Android SDK & NDK Tools** and **OpenJDK.**
- When prompted, choose the appropriate Unity license.

- With Unity installed, go to the Projects tab and create a new project

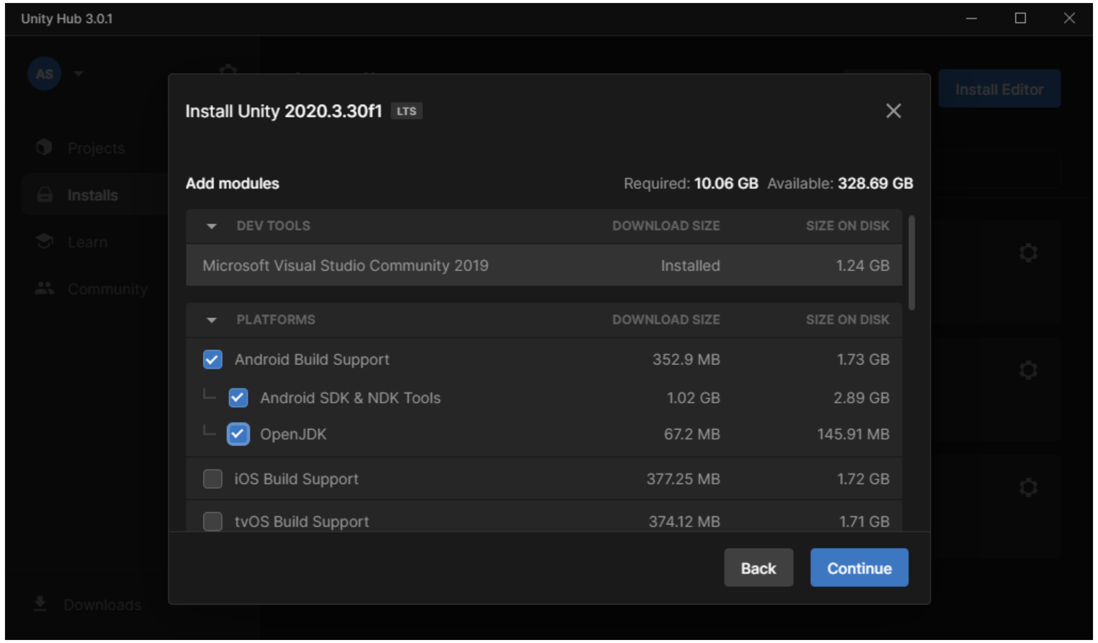

Setting up the Experiment PC

- In the New Project tab, go to All Templates and select the 2D Core template. Define your project name and location and create the project.

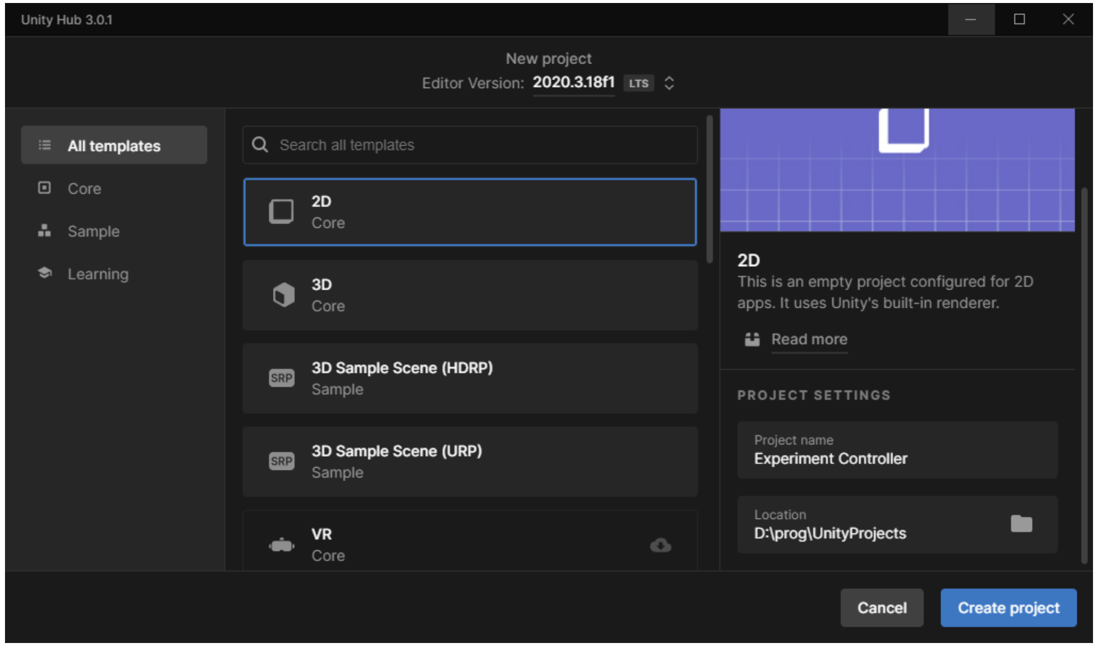

Setting up the Experiment PC

- Download the unitypackage (*ExperimentControllerFinal.unitypackage)* containing the project files from the repository https://github.com/AlysonSouza/FingerCountingVR
- In Unity, go to the Assets menu -> Import Package -> Custom Package and select the downloaded file.
- Import all the files as prompted in Unity and wait for them to load.
- After importing the files, go to Edit -> Project Settings and in the Player tab, select Other Settings and change the **API Compatibility Level** to **.NET 4.x**, as shown in the figure**.** Close the window afterwards.

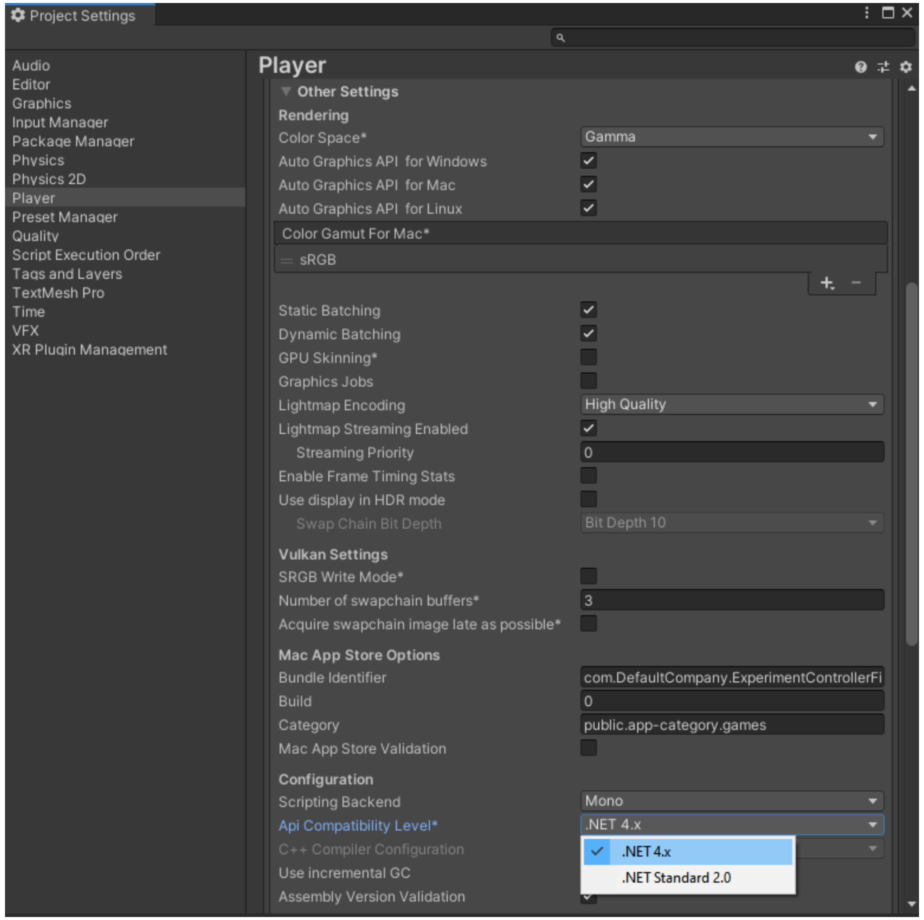

Setting up the Experiment PC

- In the Project tab, in Unity, navigate the Assets folder -> Hand Experiment -> Scenes and double click the Controller scene to open it.

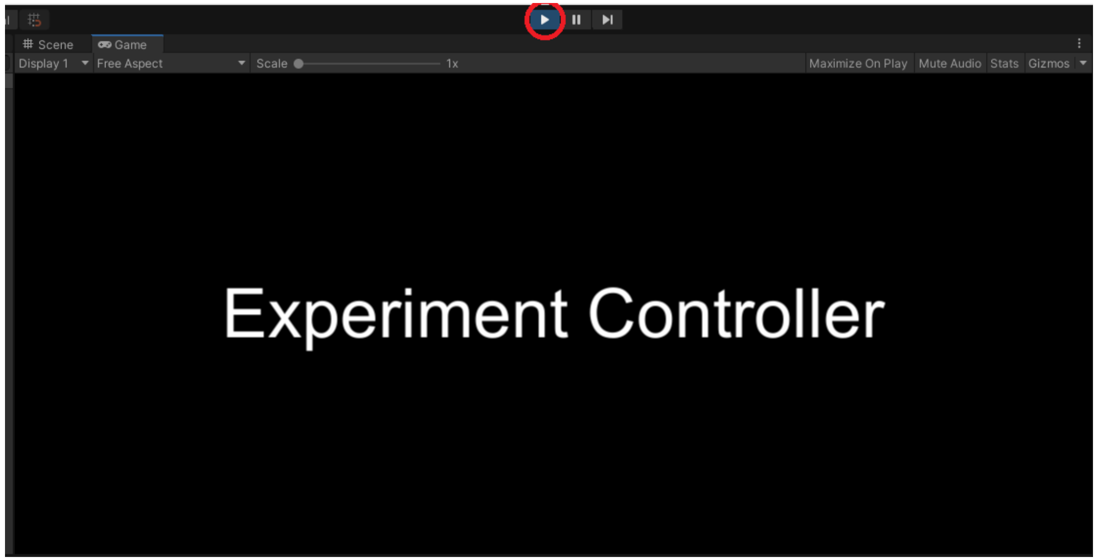

- After loading the scene, the application is ready to be executed. Press the Play button (see figure on the right, red circle) to test the application. A message saying “NO ARDUINO DETECTED” should appear on the bottom left corner with also a red text on the screen.

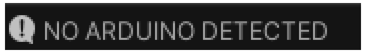

- Press Play again to stop the application.

Tactile Stimulation Device

- With the application ready to test the Arduino, we can now start to assemble the Tactile Stimulation Device.

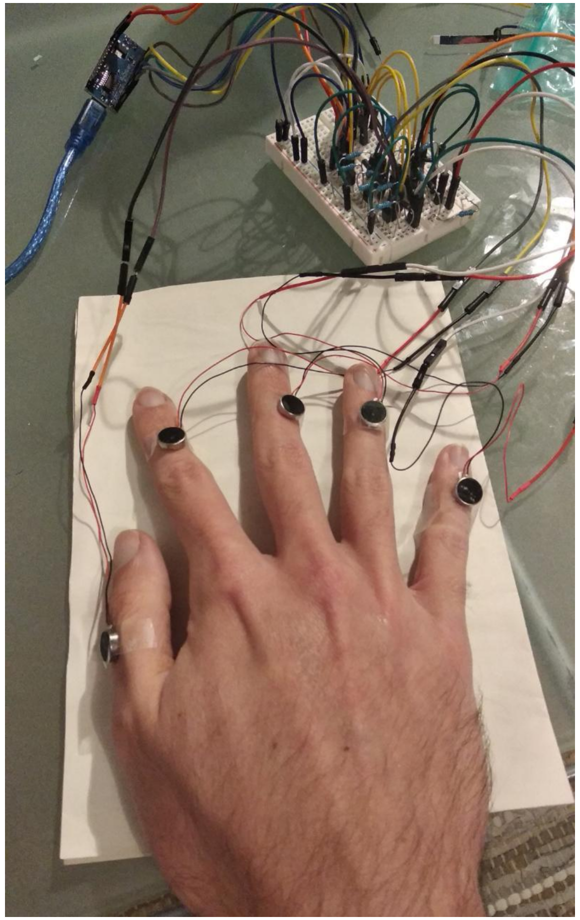

Tactile Stimulation Device

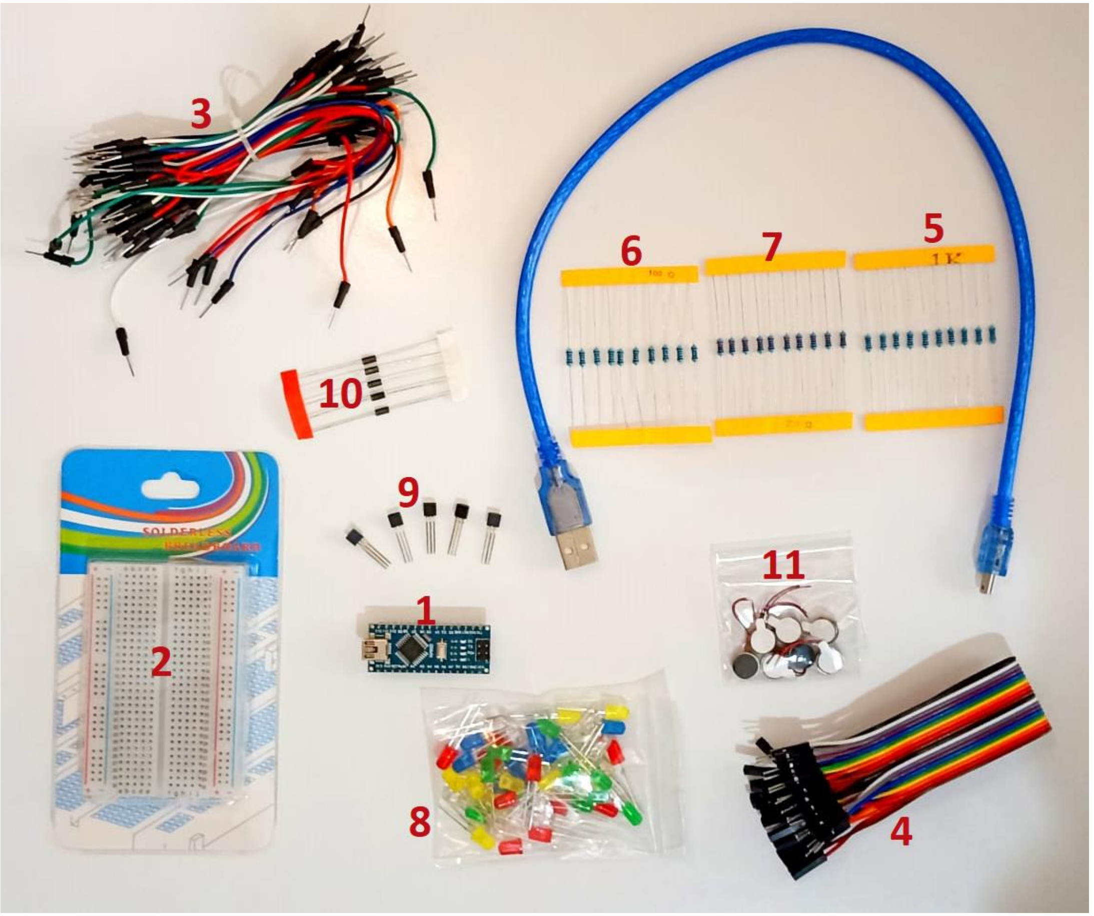

	Component	Qty
1	Arduino Nano V3 (with USB cable)	1
2	Breadboard	1
3	Jumper Wires	15
4	Jumper Wires (Female to Male)	20
5	1000 Ohms Resistor	5
6	100 Ohms Resistor	5
7	220 Ohms Resistor	1
8	LED	1
9	NPN Transistor PN2222	5
10	Diode	5
11	Coin Micro Vibration Motor	5

Installing the Arduino Software

- First, download and install the latest Arduino IDE: https://www.arduino.cc/en/software

- Follow the instructions on the official website, if needed: https://docs.arduino.cc/software/ide-v1/tutorials/Windows

Configuring the Arduino Board

- After installing the Arduino IDE, launch it, go to Tools -> Ports and see the current ports, as shown in the figure below.
- Connect the Arduino to the PC using its USB cable and go to Tools -> Ports again, to see which port the Arduino connected to.

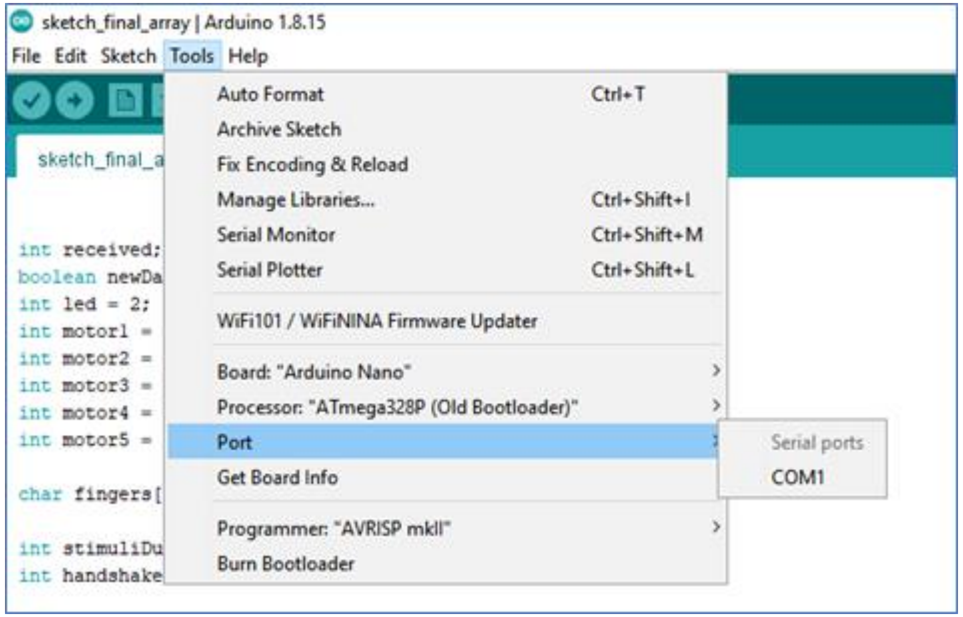

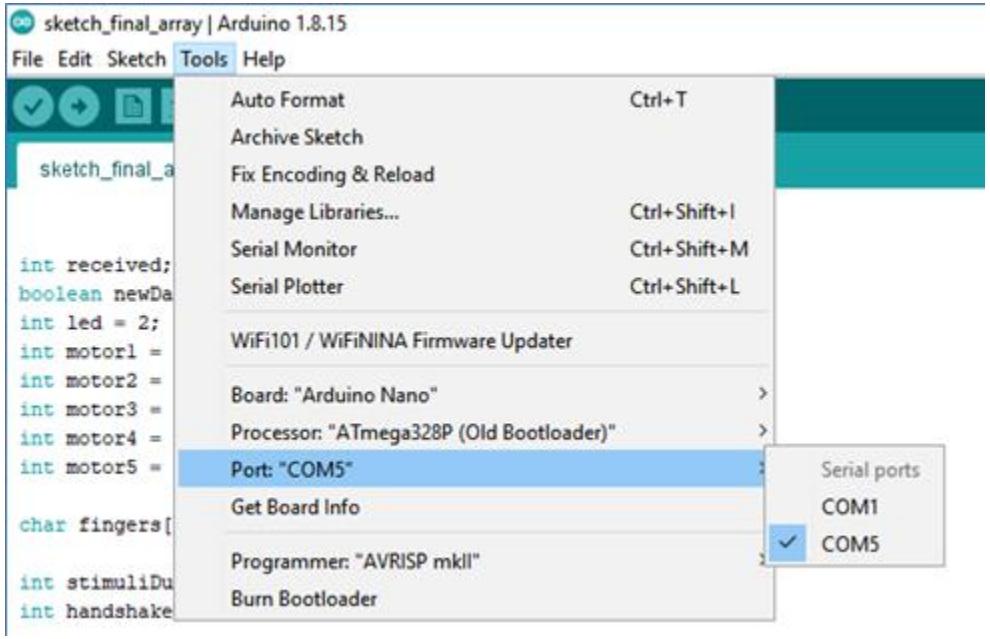

Configuring the Arduino Board

- If not already selected, choose the Board “Arduino Nano” and the Processor “ATmega328P” or “ATmega328P (Old Bootloader)” (highlighter in the figure below). This will vary depending on which Arduino was purchased. Which one is correct can be verified in the manuals that come with the Arduino. If the wrong one is chosen, the program will not upload correctly in the next step. Changing the processor should solve the issue.

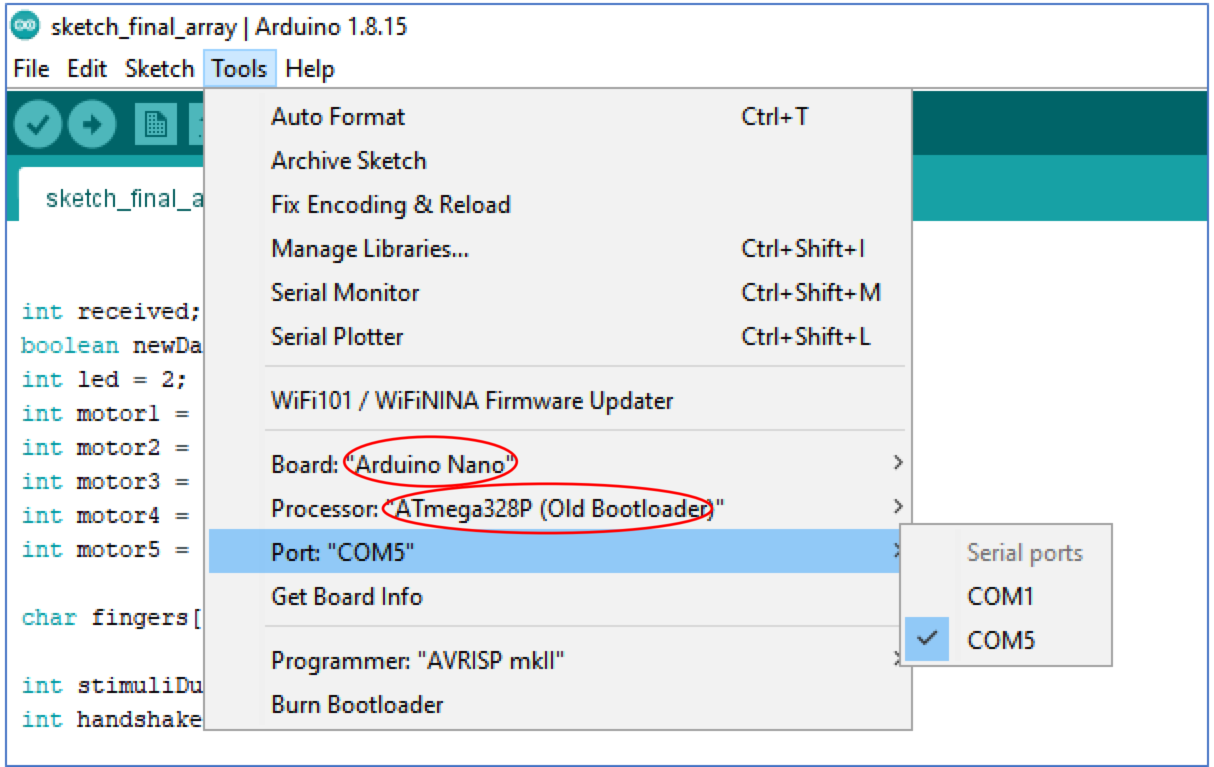

Installing the Device Firmware

- After connecting the Arduino and selecting the correct parameters in the Arduino software, the next step is to upload the firmware code.

- Download the sketch ***(sketch_array_final.ino)*** from the repository https://github.com/AlysonSouza/FingerCountingVR
- Open it in the Arduino IDE using the menu: File -> Open.

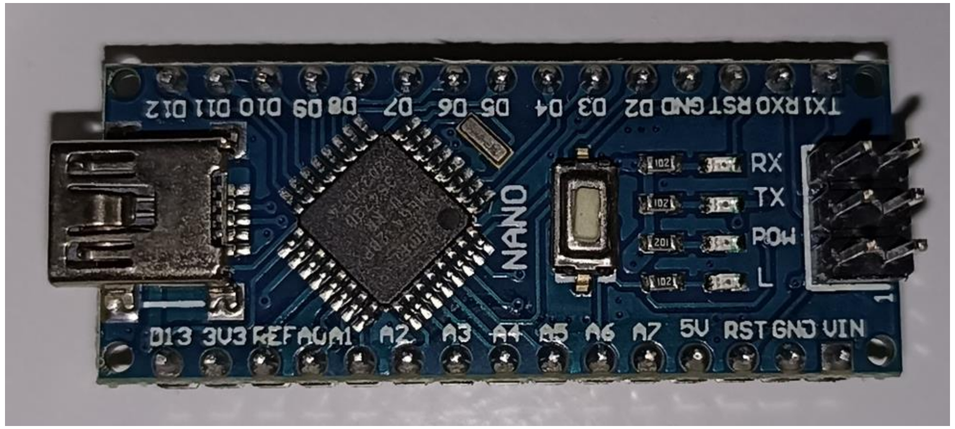

Installing the Device Firmware

- After opening the file, compile it using the checkmark button in the interface (red circle). It should compile and display the message "Done compiling".
- After compiling, upload the firmware to the board using the arrow button (orange circle). If all steps in the previous page were done correctly, the file should be uploaded successfully (Done Compiling message), and the Arduino board is now ready to be used.
- Execute the Unity application again, it should display a message saying that the Arduino was detected. This means Unity has connection with the board and is ready to use it. Stop it again afterwards.

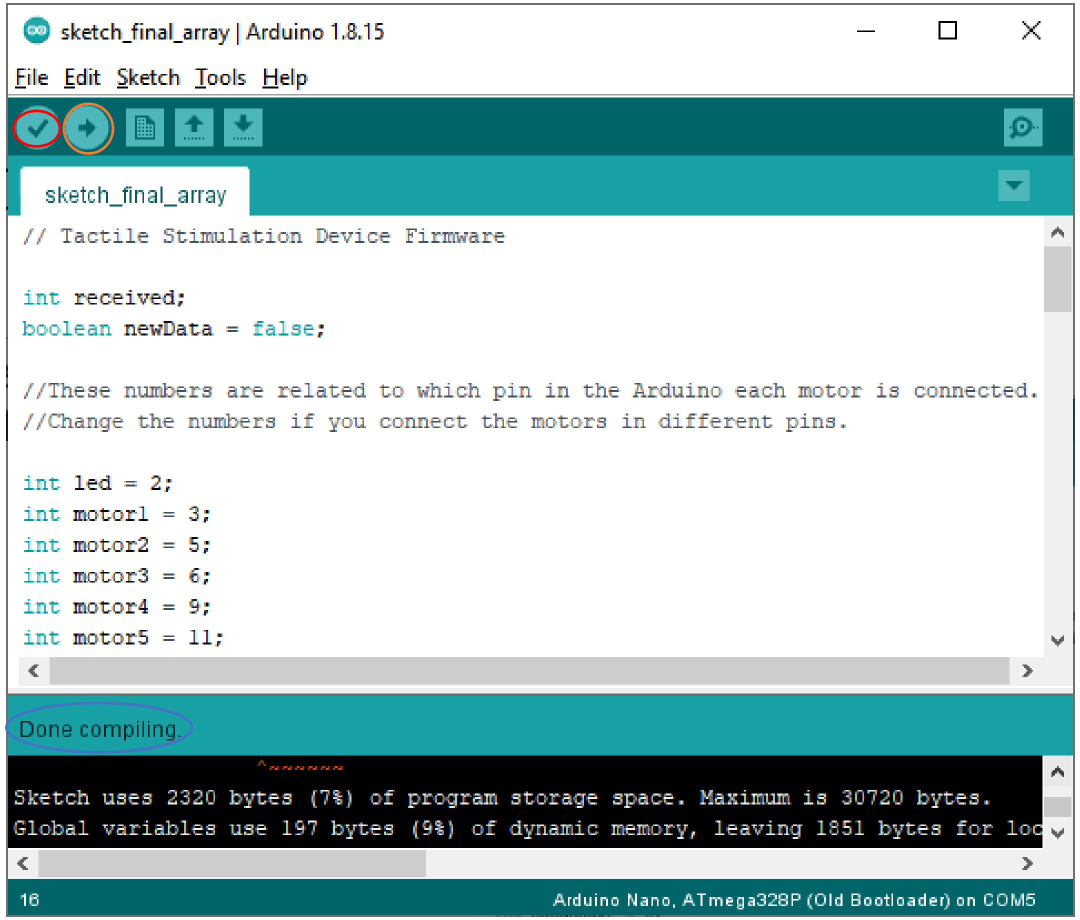

Changing the Device Firmware

- The firmware code on the Arduino can be changed as needed. The main parts of the code are in the beginning.
- motor1, for example, corresponds to the first finger and will be triggered by pressing F1 in the keyboard. The code says that it is connected to the D3 pin. Changing the corresponding number will change the pin. Always save the code, recompile and re-upload to the board after any changes.
- The other important part is the stimuliDuration. It is set to 500ms by default and can be changed in the same way as the pins. Change the number after the equals sign to set a new duration in milliseconds.
- Save, compile and upload the code to the board after changing it.
- Be careful not to delete the semicolon after the number, as it will break the code.

```
//These numbers are related to which pin in the Arduino each motor is connected.
//Change the numbers if you connect the motors in different pins.

int led = 2;
int motor1 = 3;
int motor2 = 5;
int motor3 = 6;
int motor4 = 9;
int motor5 = 11;

//This number defines the stimuli duration in milliseconds.
int stimuliDuration = 500;
```

Tactile Stimulation Device

- Now that the Arduino is ready, the next step is to create the circuit in the Breadboard. Start with the LED circuit as it is the simplest and can be used to test the board.

- Always disconnect the Arduino from the computer before connecting wires to the circuit. The device can simply be disconnected from the cable.

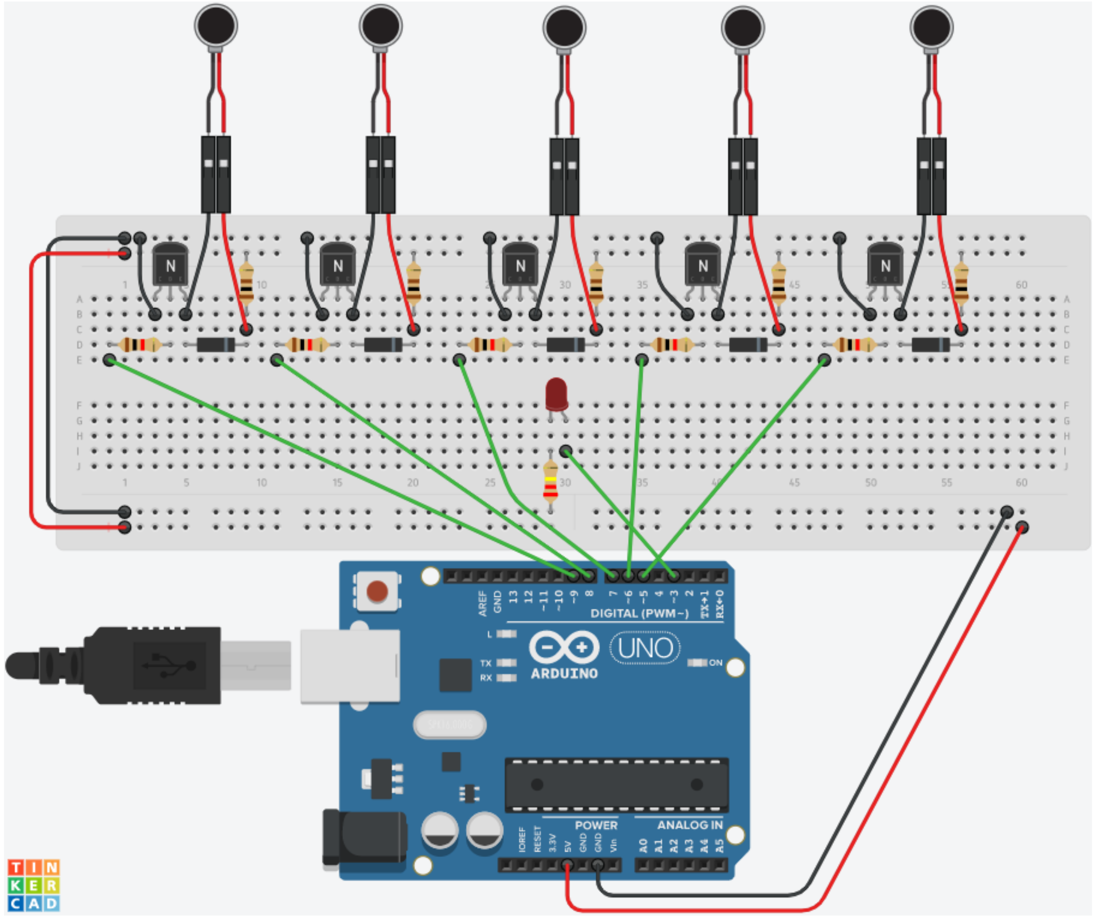

Creating the LED circuit

- Get a pair of female to male jumper wires and connect them to the 5V and GND pins of the Arduino. Note that the GND pin is the one close to the 5V pin. This will provide the circuit with electric energy and power all the components. Insert the pins all the way.

- Note that the breadboard has a column labeled + and another one labeled -. Connect the GND cable to the – column and the 5V cable to the + column on the same side of the board. Next, get a pair of jumper wires and connect the + column on one side of the board to the + column on the other side. Do the same for the – column.
  - **Be careful not to connect the + to the -!!!**

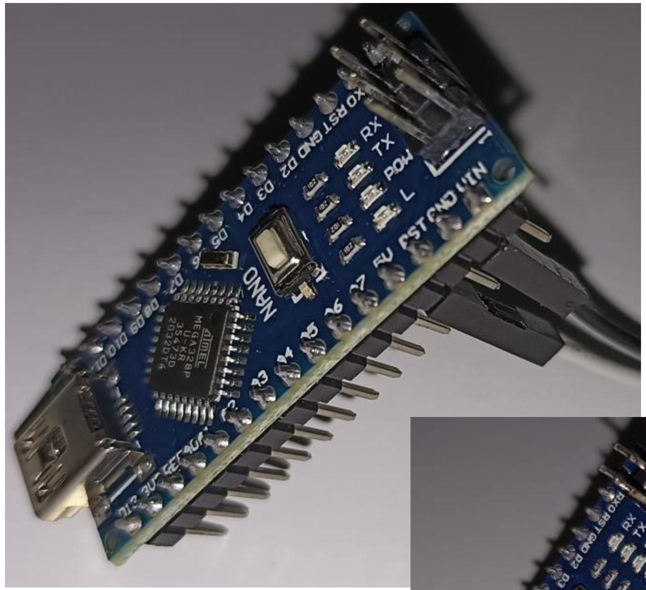

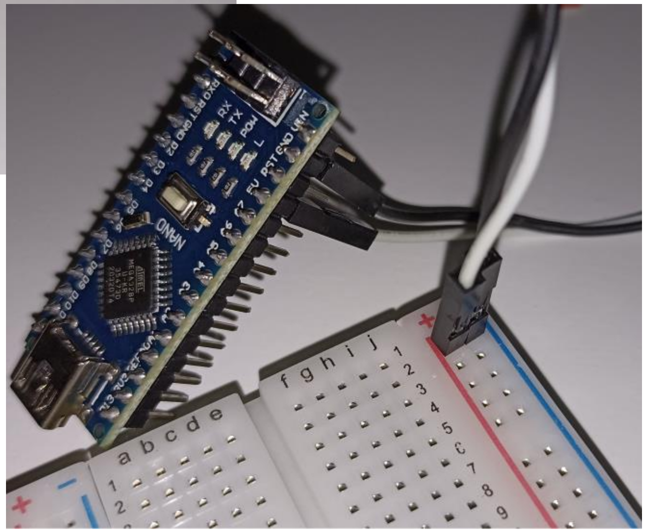

Creating the LED circuit

- Use another female to male jumper wire to connect the D2 pin to any row (row 1 was chose on the figure to the right) on the breadboard. This will be used to control the on/off of the LED based on the Arduino signals. Always refer to the diagram in Figure 4 of the paper to see the connections.

- On the row to the side of the one used for the D2 pin (row 2, on the figure), connect one pin of a 220 Ohms resistor. The other pin of the resistor should go to the – column (any row).

- Lastly, connect the LED's longer pin on the same row as the D2 wire (row 1) and the shorter pin on the same row as the resistor (row 2). Make sure to insert the pins until they touch the bottom of the breadboard.

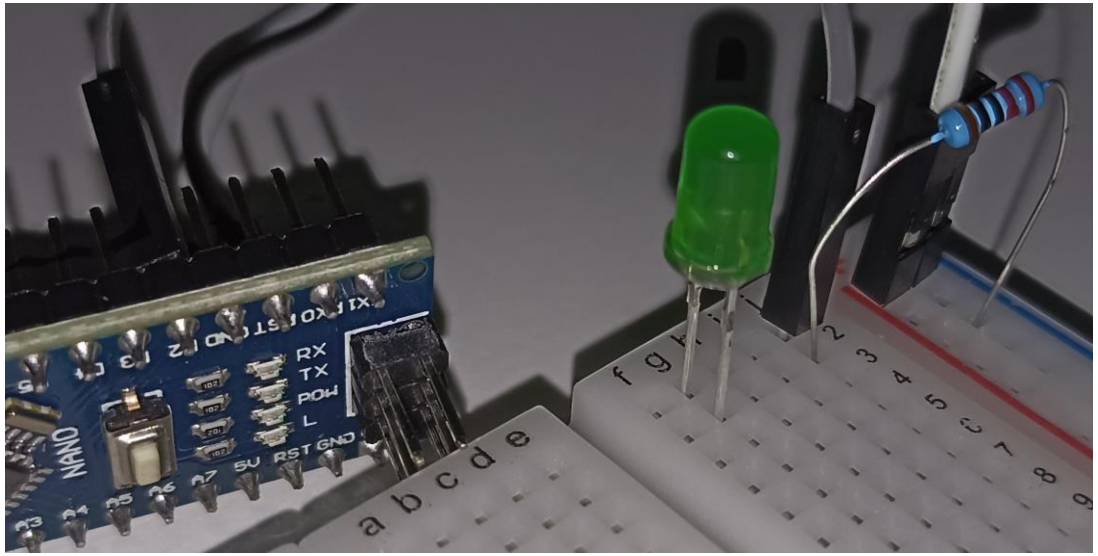

Testing the Tactile Device LED

- After connecting everything to the breadboard, reconnect the USB cable to the Arduino and connect it to the PC.
- Go back to the Unity application, press the Play button and Left click the “Experiment Controller” phrase. If the Arduino is detected, an according message will be displayed on screen (see figure on the right).
- Use the keys F1 to F6 on your keyboard to test the LED and the motors. F1 to F5 will vibrate the corresponding motor, once it is installed on the board, and F6 will vibrate them all together when the circuit is done. All keys will always make the LED blink at the same time.
- A message saying “LED ON” will be displayed on screen when the LED is supposed to be on. There is a small delay on this message, and it should only be used for debugging purposes.
- Close the program after testing.

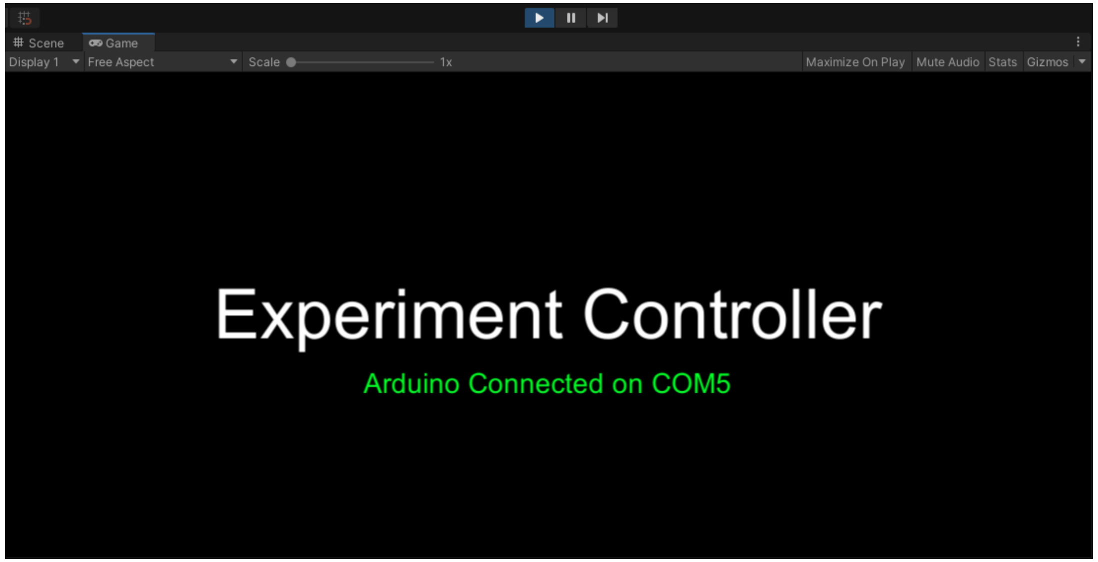

Troubleshooting

- Make sure to only press play on Unity after connecting the Arduino device, as its detection is made on the program startup.
- There should only be COM1 and the Arduino port in the system (this can be seen in the Tools -> Port menu in the Arduino software). If there are other devices, try disconnecting them before using the application.
- If Windows does not detect the Arduino board, it may be a generic one instead of the original Arduino. Search for which board it is and find the corresponding driver before using the Arduino. CH340 is a common one.
- If the LED is not lighting up after pressing the numbers in Unity, make sure first that the application is selected and is the active window (the message “LED ON” should be appearing when you press the F1-F6 key).
- If the message is appearing but the LED is not lighting up, make sure all the pins are inserted all the way into the breadboard. Also make sure the LED is connected the right way (shorter pin in the same row as the resistor). Try a different LED to see whether the one being used is broken.
- Lastly, make sure all the connections are in the right pins in the Arduino (GND, 5V and D2), or change the pin in the code (replacing D2) if you are using a different one, and upload it again to the board.

Creating the First Motor Circuit

- Each motor circuit in comprised of the following components, as seen in the diagram:
  - 1 Diode
  - 1 NPN Transistor PN2222
  - 1 Coin Micro Vibration Motor
  - 1 100 Ohms Resistor
  - 1 1000 Ohms Resistor
  - 3 Dupont Wires
  - 1 Jumper Wire

- Its important to note that both the diode and the transistor must be connected as shown in the diagram. The transistor's pins will always be described as if the flat surface is facing the reader. The diode has a thin grey line indicating its position.

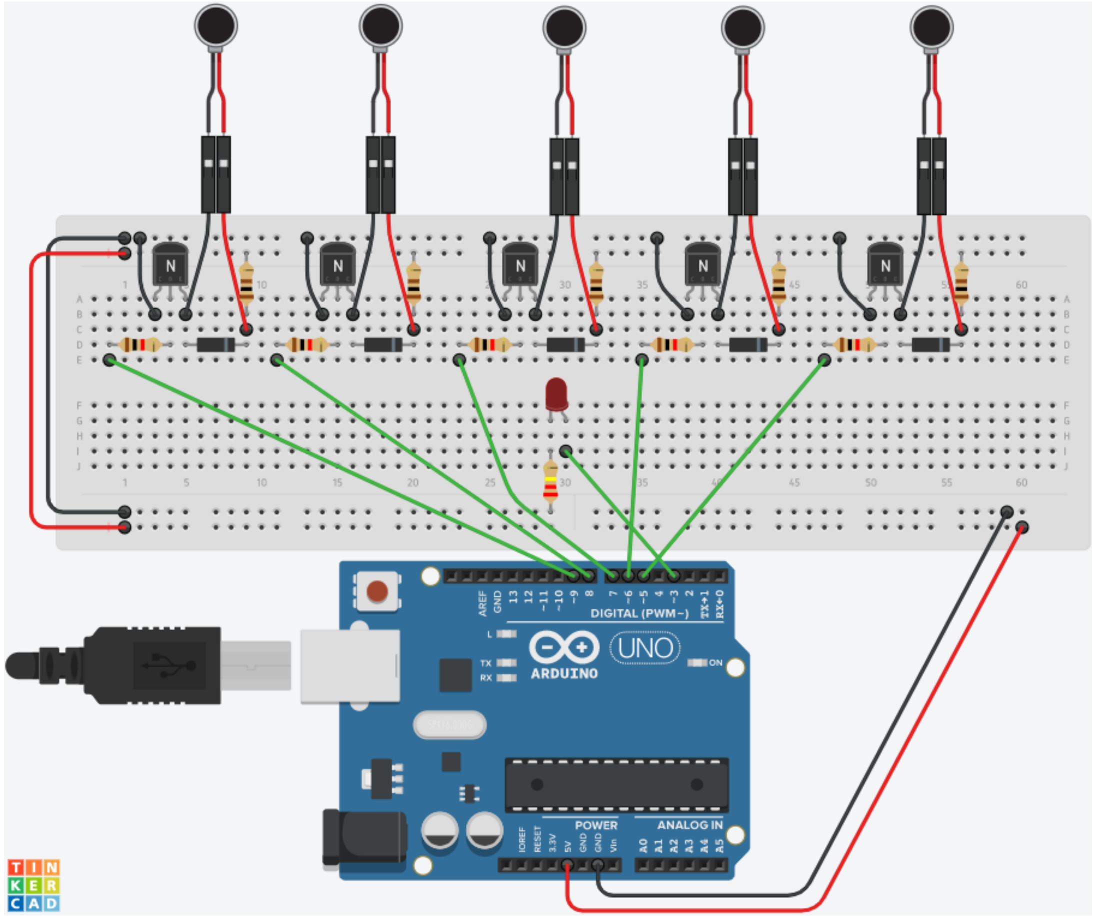

Creating the First Motor Circuit

- The first step again is to connect the port from the Arduino into the breadboard. Be mindful of the space being used, specially if using a small breadboard as the one shown here. Remember that each row must **only** have the components described in the diagram. Placing different components in the same row will make them connected and may damage the components if done wrongly.

- Make sure to always insert the pins all the way down on the breadboard.

- Always make sure **no pin is touching one another** as this may cause a short in the circuit, as highlighted in the image.

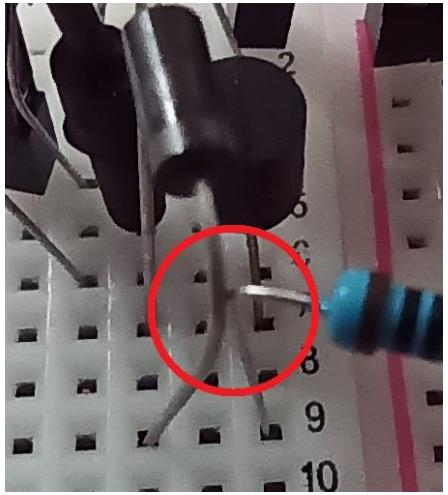

Creating the First Motor Circuit

- Connect the D3 pin of the Arduino to a free row on the breadboard using a female to male jumper wire (row 4 in the image, in red – the rows used do not need to follow the numbering shown here but the one used in the image will always be displayed in bracket for clarity).

- Connect one pin of a 1000 Ohms resistor to the same row as the D3 pin wire (row 4 on the figure, in orange). Connect the other pin of the resistor to a free row nearby (e.g., line 6 as in the figure, in orange). Leave one empty row (5) for the transistor later.

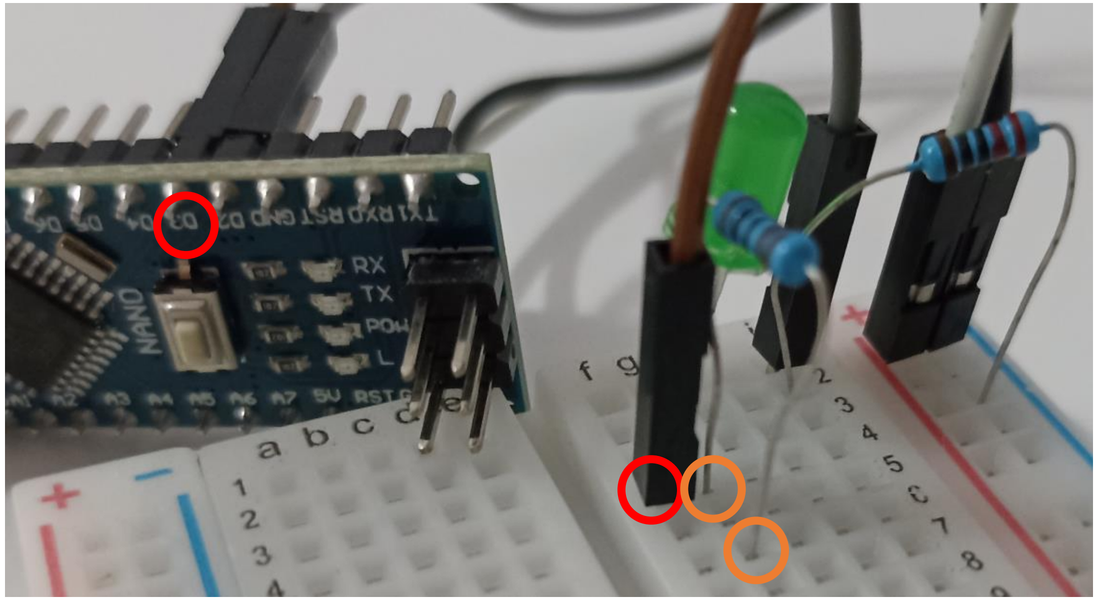

Creating the First Motor Circuit

- Place the transistor with the central pin in the same row as the second pin of the 1000 Ohms resistor (row 6 in the figure). Place the flat side of the transistor pointing to the resistor, with the two pins on free rows (5,7). Try to use the last column (column j) for the transistor, as it will make it easier to mount the other components.

- After that, the transistor will be, in this design, in column j, rows 5, 6 and 7.

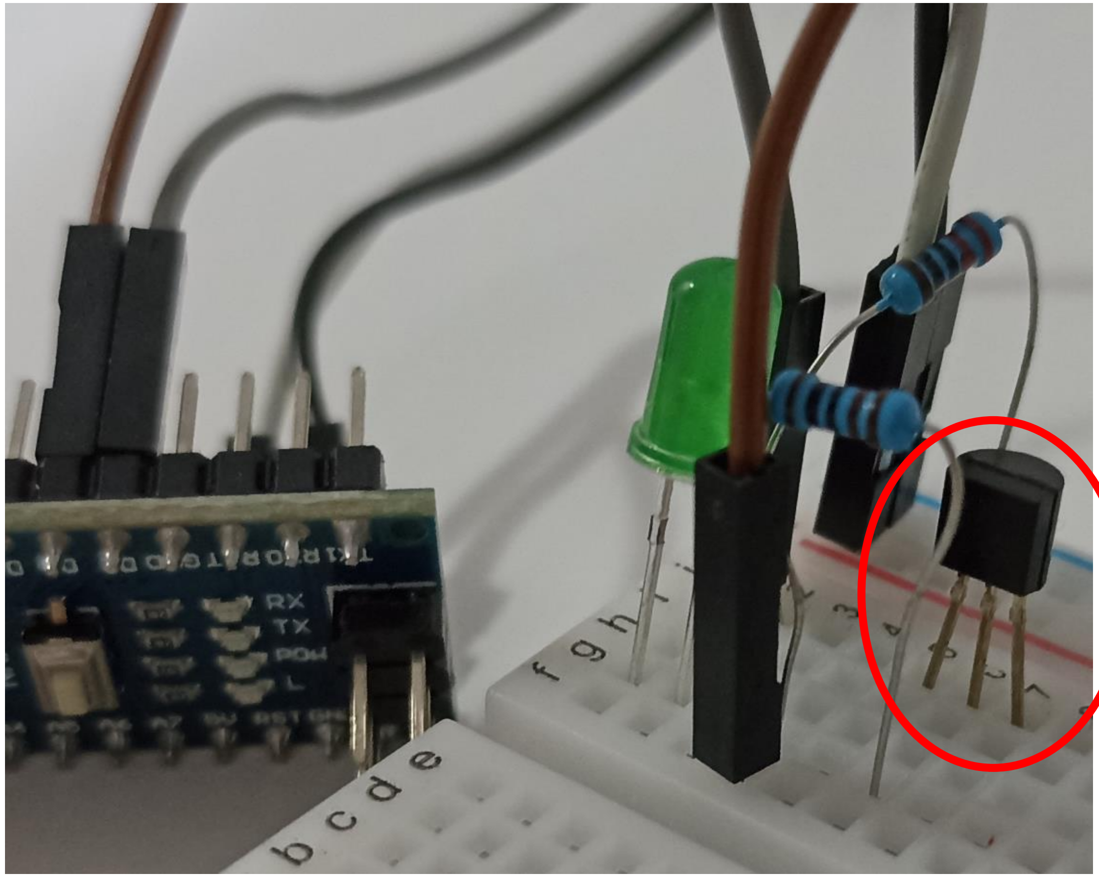

Creating the First Motor Circuit

- Use a Jumper Wire to connect the row of the left pin of the transistor (row 5 in the figure) to the – column of the breadboard.

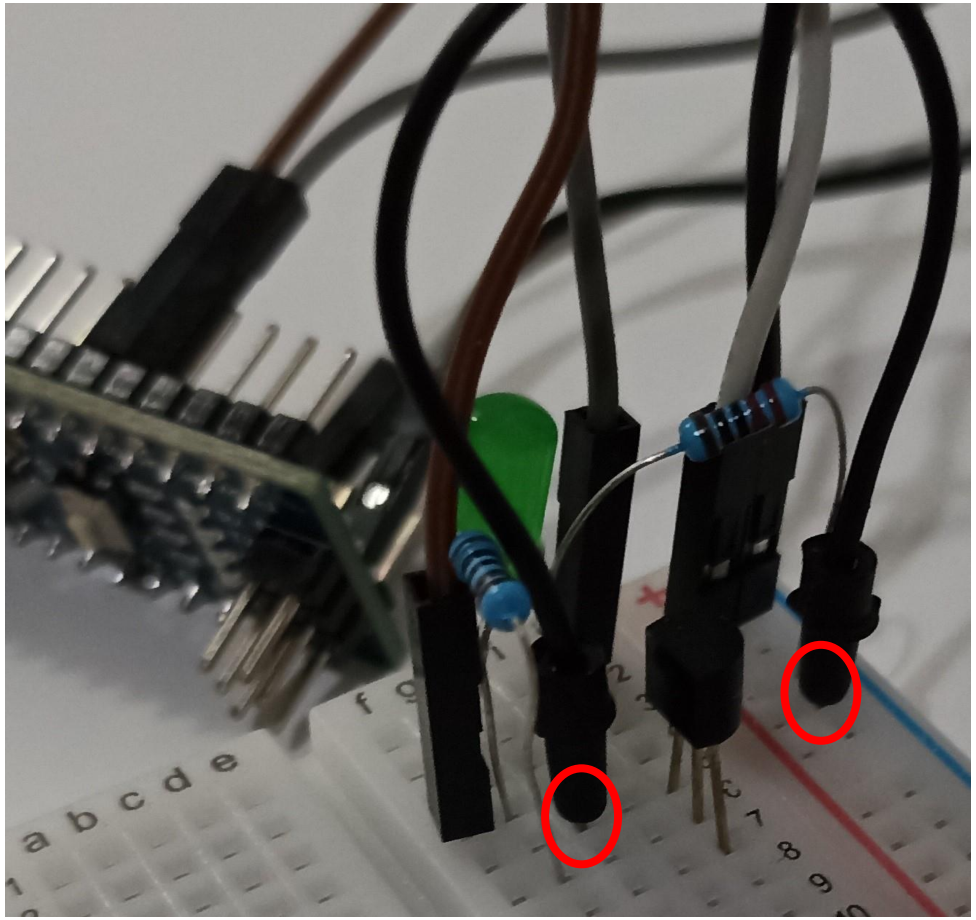

Creating the First Motor Circuit

- Connect the side **without** the grey mark of the diode to the same row as the right side of the transistor (7). Connect the side **with** the grey mark to a nearby free row (9).

- Remember to use the grey line as reference for the diode, as highlighted in the image.

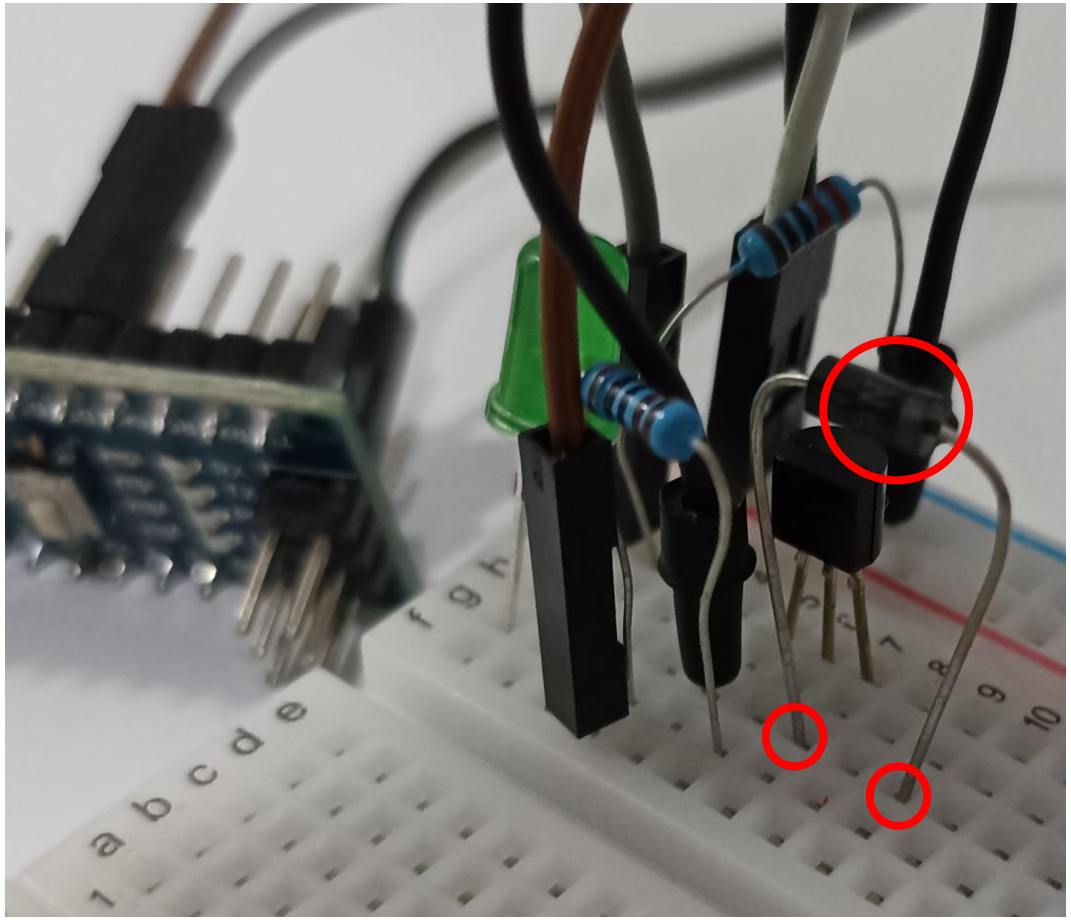

Creating the First Motor Circuit

- Use a 100 Ohm resistor to connect the row of the diode's marked side (9) to the + column.

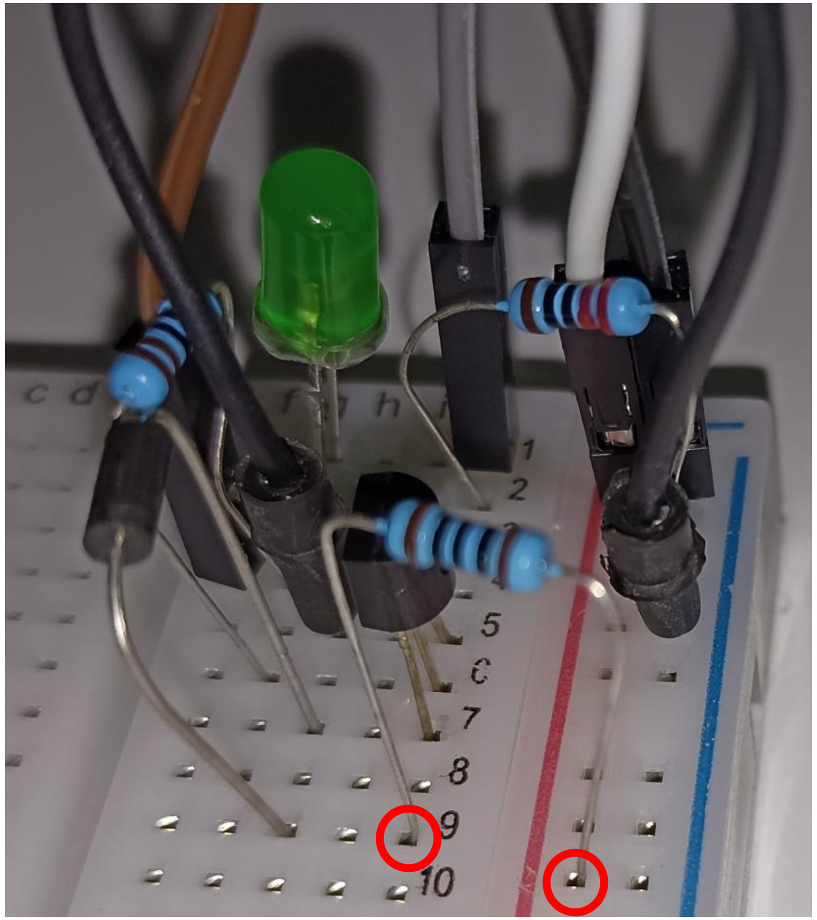

Creating the First Motor Circuit

- The last step is to add two female to male jumper wires to the circuit so the motor can be connected.
- Add one female to male jumper wire to the same row as the right side of the transistor and the unmarked side of the diode (7).
- Add another female to male jumper wire to the same row as the marked side of the transistor and the 100 Ohm resistor (9).
- **Notice that the row 8 is not used here!**

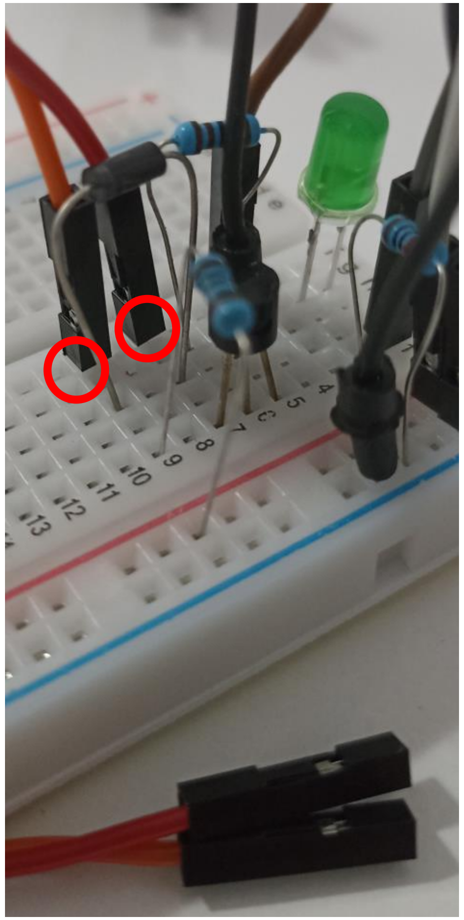

Creating the First Motor Circuit

- The motor circuit is now ready to be used. It is possible, as described in the paper, to simply put the motor wires in the female connectors of the Dupont wire and it will work. The motor can be connected in any orientation. The vibration will eventually make the motor disconnect from the Dupont wire and stop, but it can be tested this way with some care, if desired.

- The next step is to solder the motors with some jumper wires for a more stable connection.

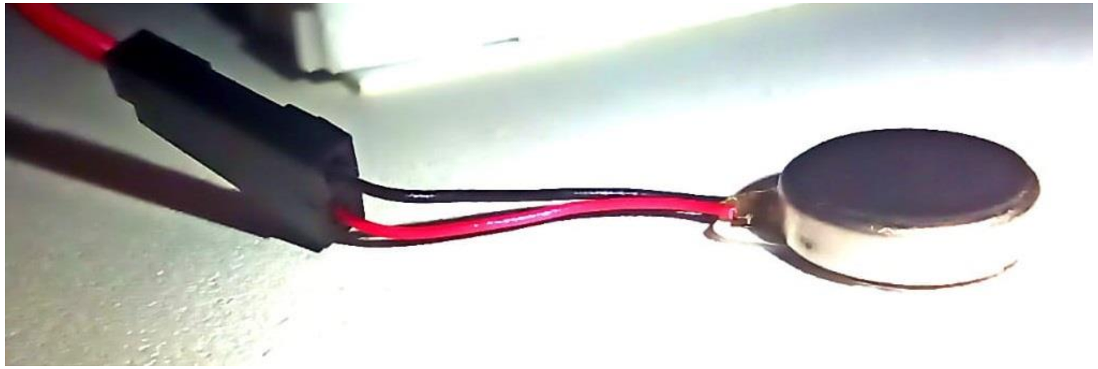

Soldering the motors for better stability

- To create a more reliable connection between the motors and the Dupont Wires, soldering them to jumper wires is recommended.

- Grab a pair of scissors or pliers and cut a jumper wire in half so we have two small cables with connections. If a longer cable is desired, cut just the connector out of a jumper wire and keep the other connector with the longer wire.

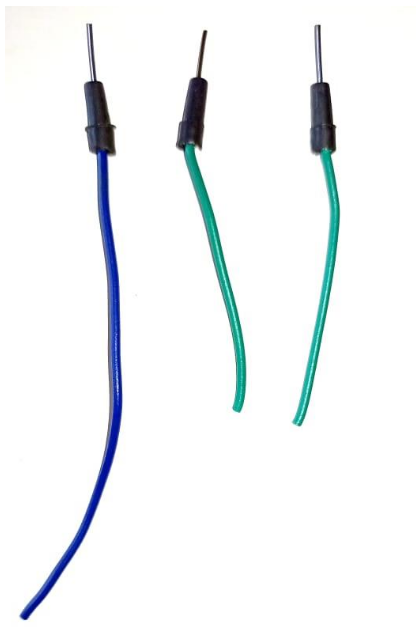

Soldering the motors for better stability

- Use a Wire Stripper or any cutting tool to carefully remove the insulation from the wires. Be careful not to cut the wires inside the plastic.
- Add the thermo-retractable tube to the wires now, if opting to use them.

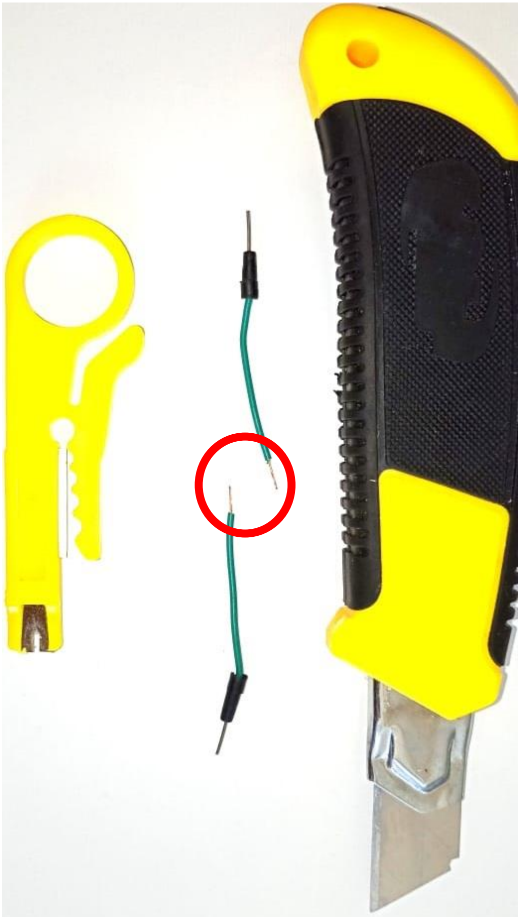

Soldering the motors for better stability

- Now solder the exposed part of the motor’s wire to the exposed part of the jumper wires. Do it for both wires.

- This will make it so the jumper wire connectors can be used with the Dupont wires for the motors, making their connection more stable.

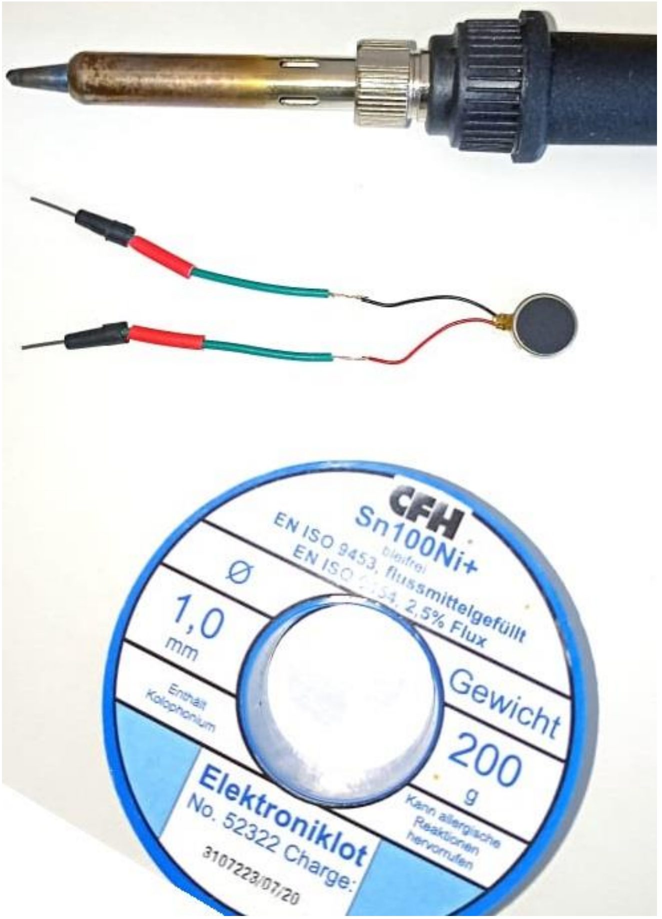

Soldering the motors for better stability

- After soldering the connections, cover them with the thermo-retractable tubes and warm them so they contract. Be careful not to melt the wires.

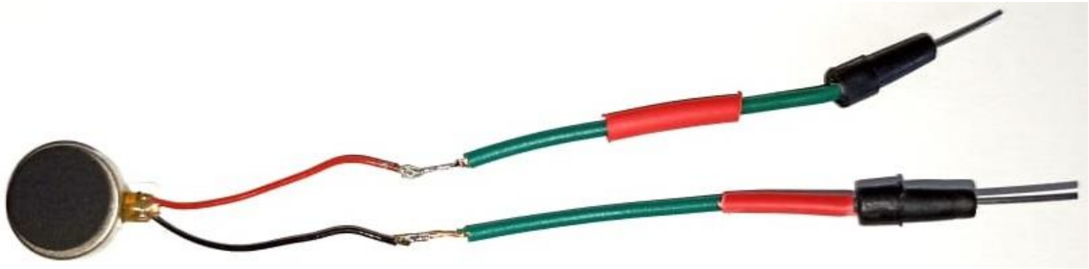

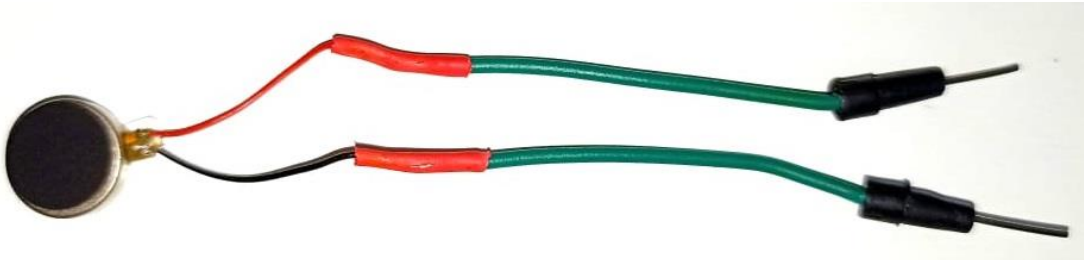

Soldering the motors for better stability

- Connect the motors to the board using the newly soldered connectors and the corresponding female side of the female to male jumper wires that were added before.

- Notice that in some cases the wire will not go in fully, this is not a problem if the connection is stable enough so that is does not fall when vibrating.

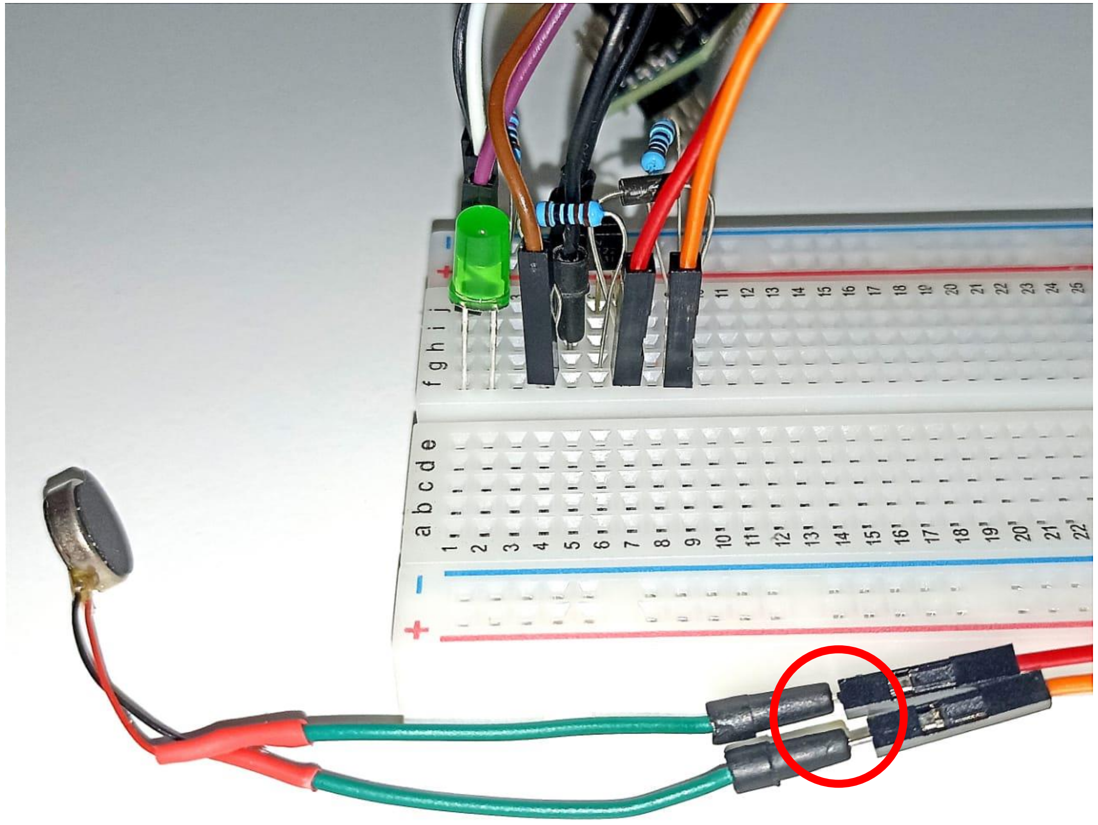

Testing the Tactile Device Motor

- After connecting everything to the breadboard, reconnect the USB cable to the Arduino and connect it to the PC.
- Go back to the Unity application, press the Play button and Left click the “Experiment Controller” phrase. If the Arduino is detected, a message will be displayed on screen.
- Use the keys F1 to F6 on your keyboard to test the LED and the motors. F1 to F5 will vibrate the corresponding motor, once it is installed on the board, and F6 will vibrate them all together when the circuit is done. All keys will always make the LED blink at the same time.
- Notice that the numbers pressed in the keyboard are corresponding to the ports in the code:

```
//These numbers are related to which pin in the Arduino each motor is connected.
//Change the numbers if you connect the motors in different pins.

int led = 2;
int motor1 = 3;
int motor2 = 5;
int motor3 = 6;
int motor4 = 9;
int motor5 = 11;
```

- In this example, pressing F1 will trigger the pin D3 in the Arduino, which we used in the example for the first motor. Sequentially, as shown in the code above, F2 will trigger the pin D5 and so on. The LED should always light up while the motor vibrates (if it is connected to the D2 pin as it should).

Troubleshooting

- Make sure to only press play on Unity after connecting the Arduino device, as its detection is made on the program startup.
- There should only be COM1 and the Arduino port in the system (this can be seen in the Tools -> Port menu in the Arduino software). If there are other devices, try disconnecting them before using the application.
- If Windows does not detect the Arduino board, it may be a generic one instead of the original. Search for which board it is and find the corresponding driver before using the Arduino. CH340 is a common one.
- Make sure all the connections are in the right pins in the Arduino or change the pin in the code if you are using a different one and upload it again to the board.
- See if all the pins are fully connected and in the right row, as displayed in the diagram. Change components one at a time, always disconnecting the Arduino from the PC before doing so, if the circuit is still not working. You may find a faulty one. Test in the order: new motor, new transistor, new diode, new resistors, new wires, new breadboard – always reconnecting after changing each.

Finalizing the Tactile Stimulation Device

- Now that the device has the LED and one motor circuit working, the same motor circuit just needs to be repeated four more times on the board to finish the device. Leave one or two empty rows between each motor circuit to create more space to install the components.
- As stated before, the ports used in the default firmware are as follows:
  - LED – D2
  - Motor 1 (Thumb Motor) – D3
  - Motor 2 (Index Finger Motor) – D5
  - Motor 3 (Middle Finger Motor) – D6
  - Motor 4 (Ring Finger Motor) – D9
  - Motor 5 (Pinky Finger Motor) – D11
- Make sure every row has only the correct components connected to it and that every component is inserted fully into the board.
- It is also recommended to use a Jumper Wire to connect the + column on one side of the board to the + column on the other side of the board, so they both become functional. Do the same for the – column.

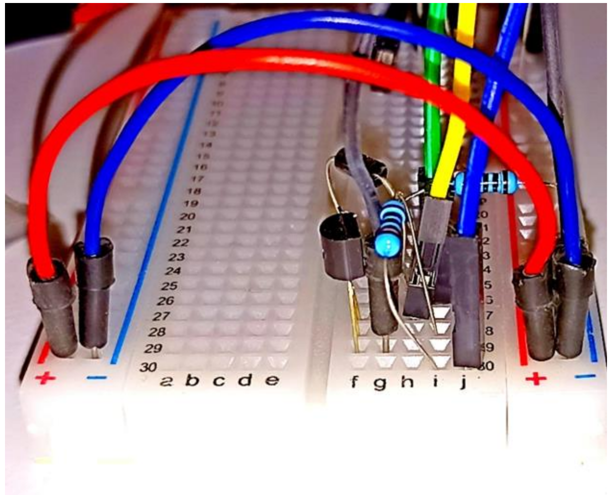

Fixating the Motors to the User's Fingers

- To fixate the motors to the user's fingers, any type of tape can be used, but this may damage the wires if not careful.

- A better option is to use small hook-and-loop fastener piece and attach them to the motors. Afterwards, the motors can be attached to the fingers using the other side of the fasteners or an adequate fabric, as described in the paper.

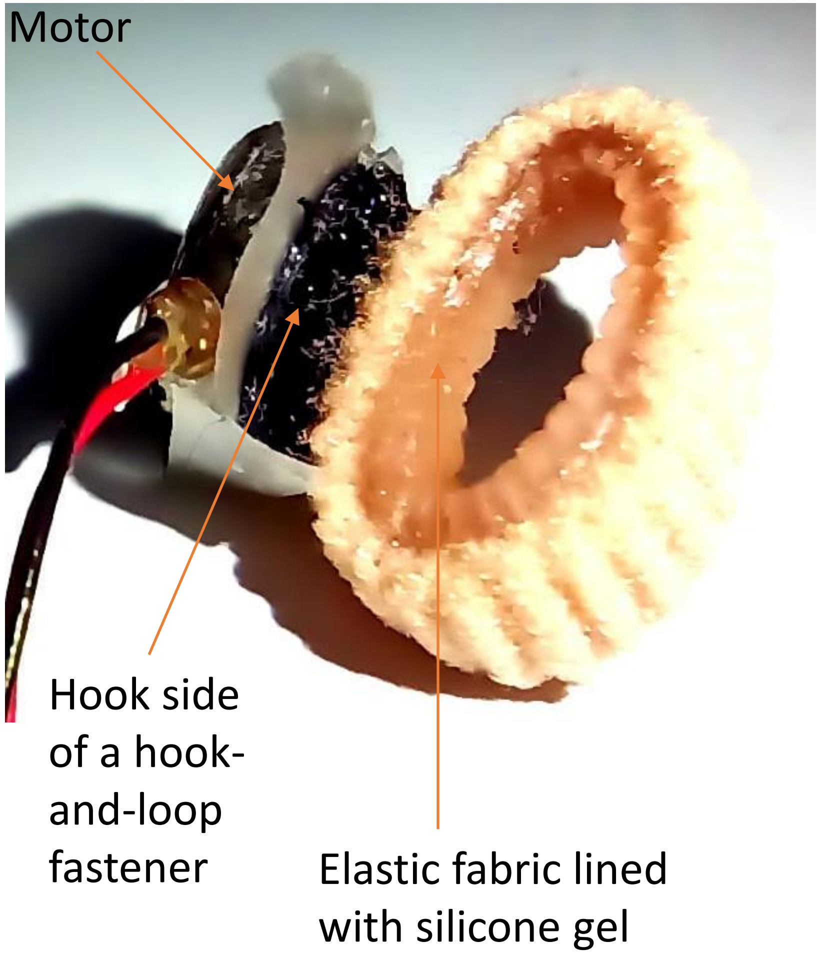

Setting up the Network Communication

- After all the five motors and the LED are connected and tested using the Unity application, the network communication can be setup.
- For the application to work with the VR Headset, the Experiment PC and the VR Headset must be in the same network and the network ports should be open. The IP field must have the correct network address **of the other device**, in each Network Controller (both on the VR Application and the Experiment Controller).
- If you need to change the ports in the application, you can do so by selecting the network controller in the **Hierarchy** (top image) and changing them in the **Inspector** (bottom image).
- Make sure the Application is receiving on the same port the VR Headset is sending and sending on the port the VR Headset is receiving (this is the Default).

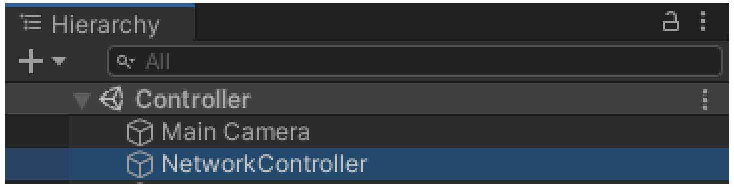

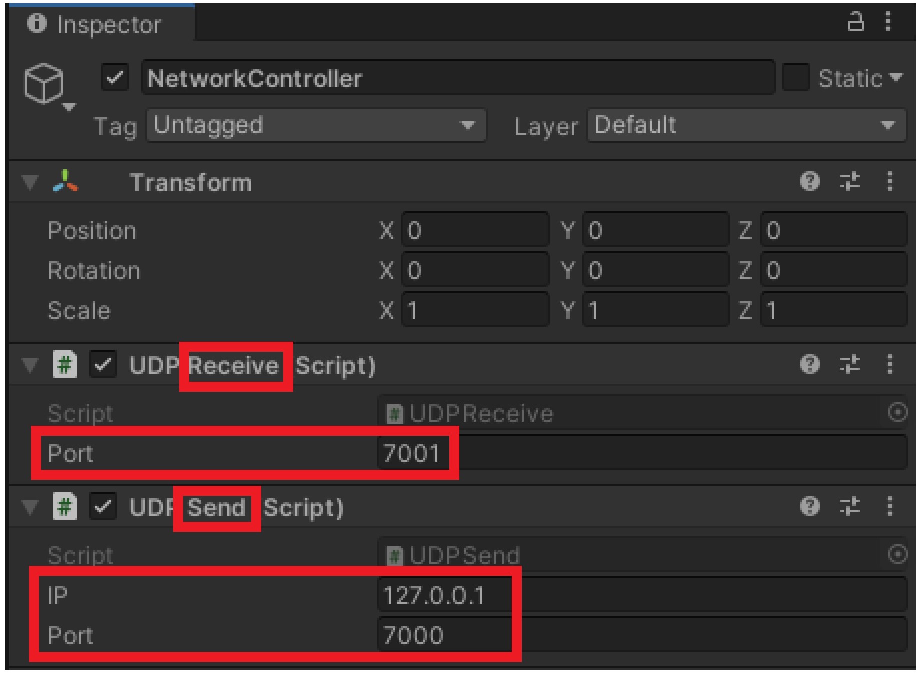

Setting up the Network Communication

- The application is now ready to communicate with the VR application and transfer the requests to the Tactile Device. Click play with the Arduino connected to execute the application and keep it ready to receive requests from the VR Headset.

- Refer to the Supplementary Material 2 for instructions on how to setup the headset and test the connection with the Experiment PC.

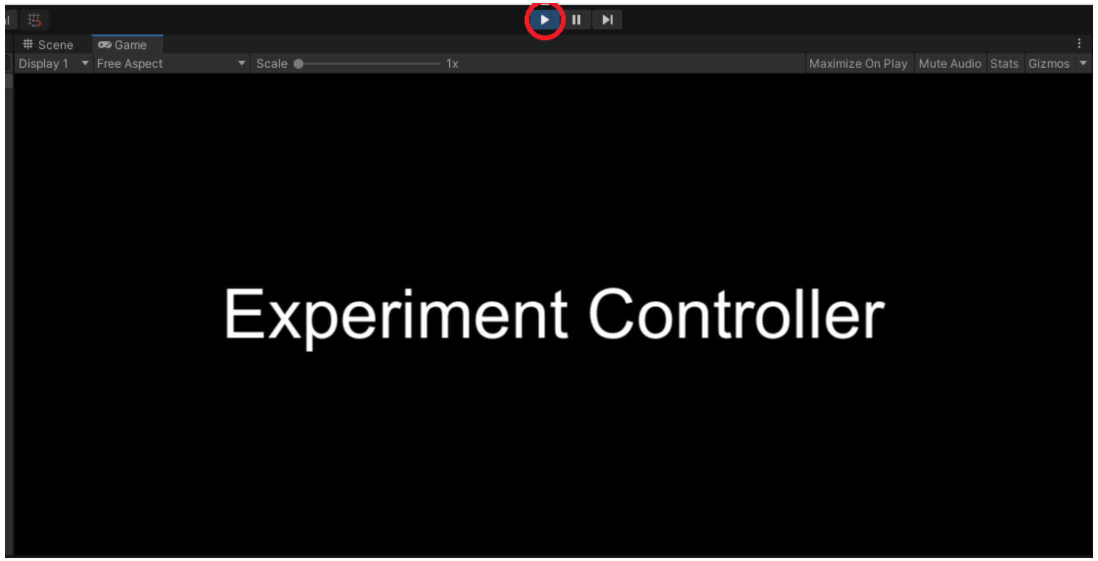

Tactile Stimulation Device

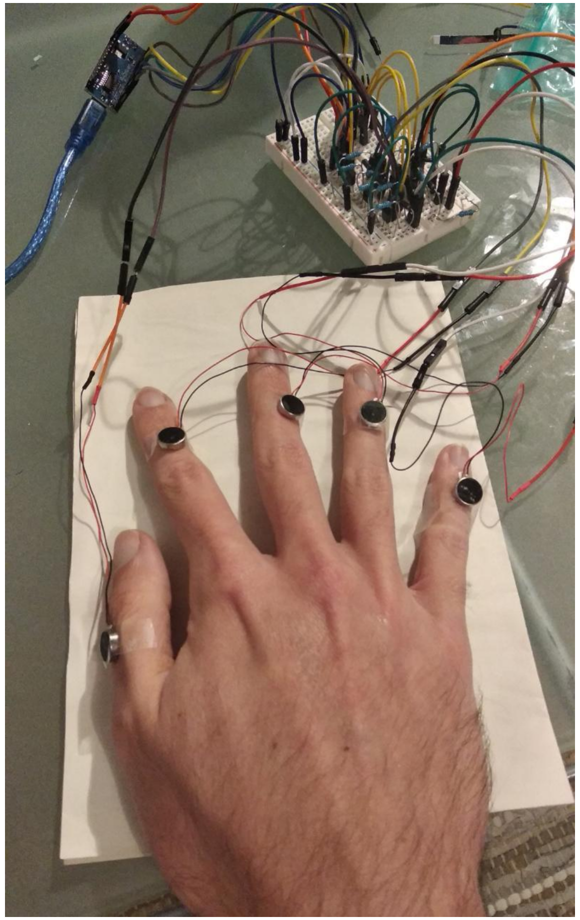

Supplement: Supplementary file 1 [file Presentation_1.pdf]
