# Supplementary material for: Combining virtual reality and tactile stimulation to investigate embodied finger-based numerical representations
Source: Front Psychol. 2023 Apr 27;14:1119561. doi: 10.3389/fpsyg.2023.1119561 (PMC10174462; doi:10.3389/fpsyg.2023.1119561)

Combining virtual reality and tactile stimulation to investigate embodied finger-based numerical representations

Supplementary Material 2

Virtual Reality System

Equipment

**Equipment**	**Quantity**
Oculus Quest VR Headset	1
Experiment PC	1
USB-C to USB-C<br>or USB-C to USB cable (according to available computer port – USB 3.0 recommended)	1

Setting up the Unity Project

- Download and Install Unity version 2020.3 and its Android Support components, as described in Supplementary Material 1.
- Create a new Unity Project using the 3D Core as the template. Choose a relevant project name.
- After opening the new project, go to File -> Build Settings (Ctrl + Shift + B, on Windows)

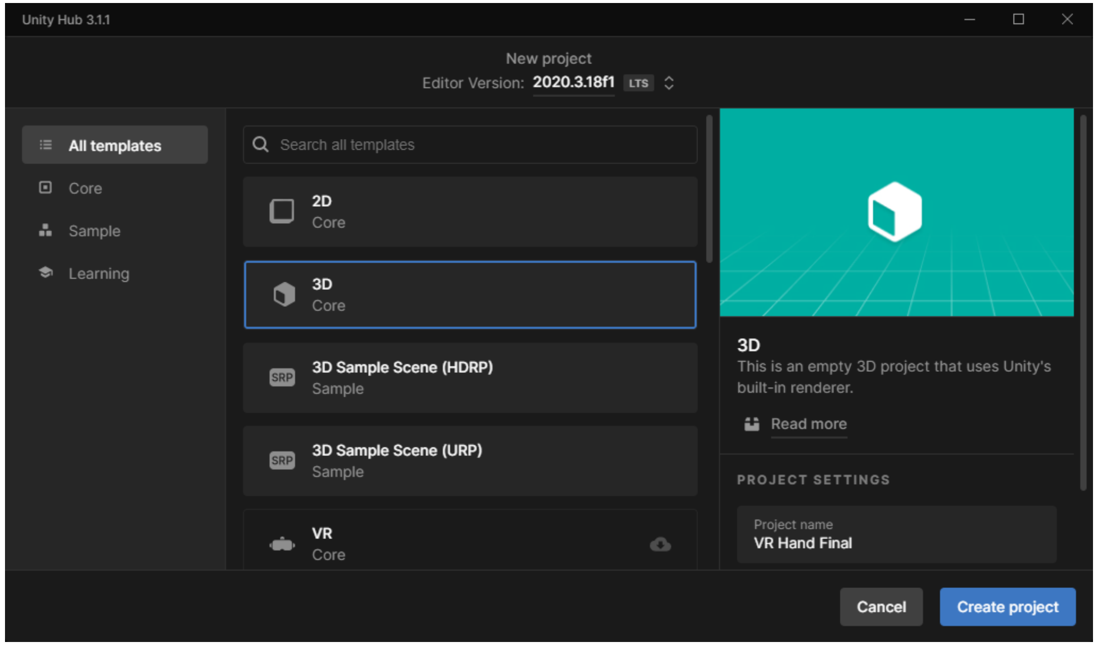

Setting up the Unity Project

- In the Build Settings window, choose the Android option, change the Texture Compression to ASTC and click Switch Platform. The image on the right has the final configuration.

- After switching the platform, the Unity logo marker will switch from PC, Mac & Linux Standalone to the Android line.

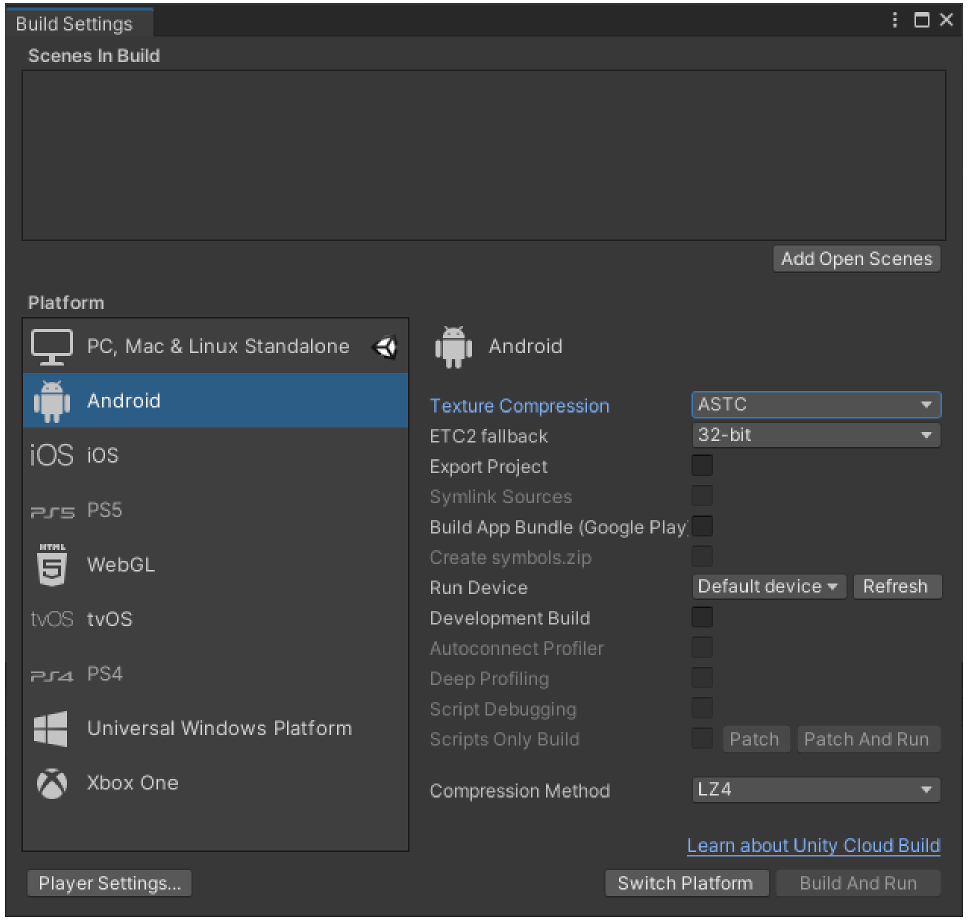

Adding Oculus Quest Support to the Project

- Download the Oculus Integration SDK from the link: https://developer.oculus.com/downloads/package/unity-integration-archive/35.0

- In your Unity Project go to the menu Assets -> Import Package -> Custom Package and select the downloaded file.

- Import all files when prompted. The importing may take a while.

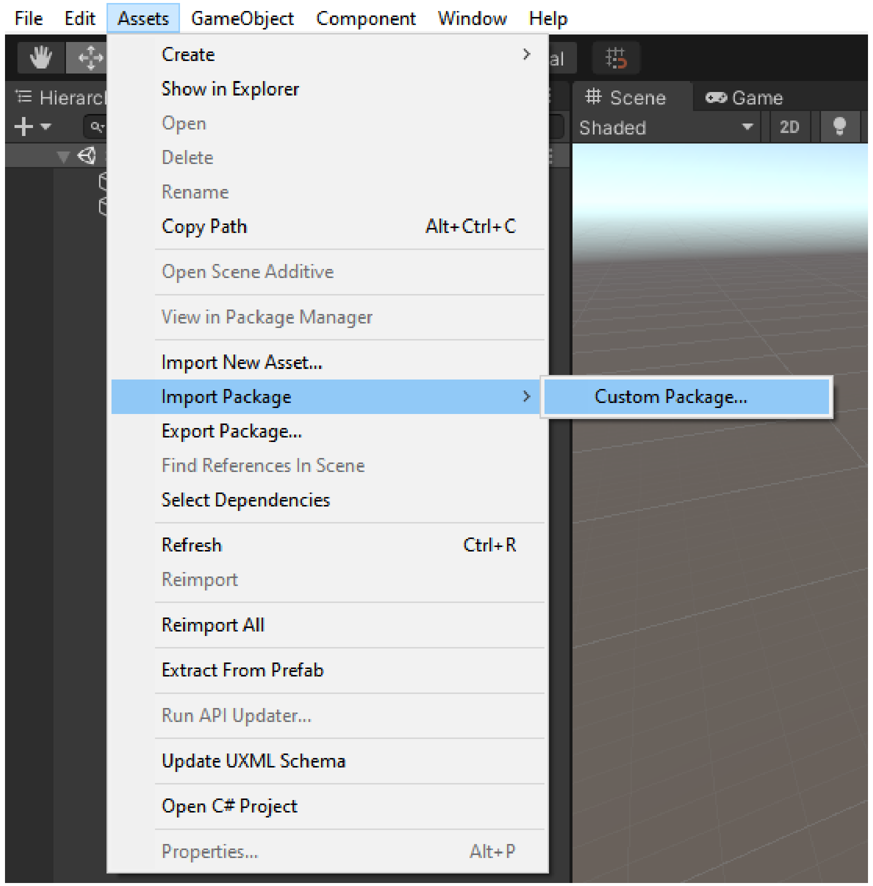

Adding Oculus Quest Support to the Project

- When prompted to update the Oculus Utilities plugin, click **Yes**. Then click **Cancel** on the OpenXR window. Click **Ok** on the next one and then click **Restart**. Lastly, click **Upgrade** when asked about the Spatializer and click **Restart** again.

- Wait for Unity to restart.

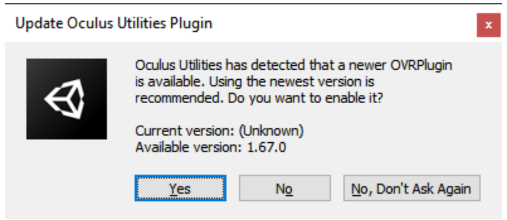

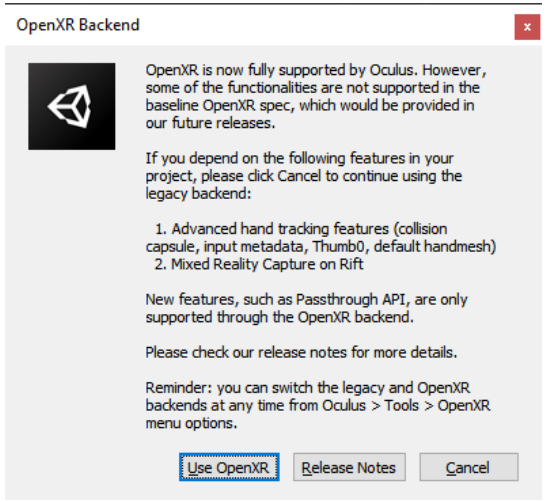

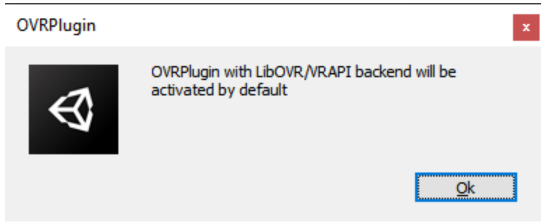

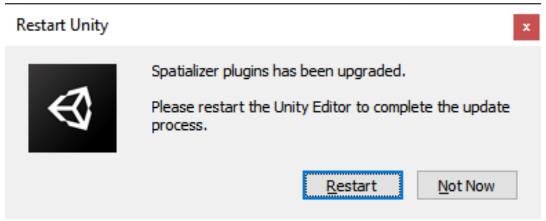

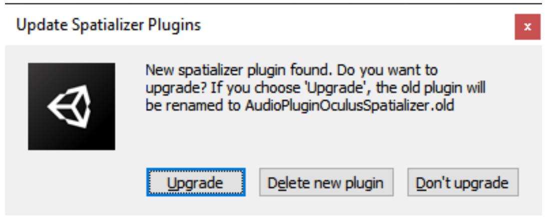

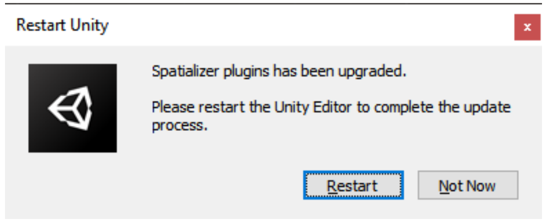

Configuring the Unity Project

- Configure the names in the project. In the menu Edit -> Project Settings -> Player chose a Company Name, a Product Name and a Version (can use the one already there).

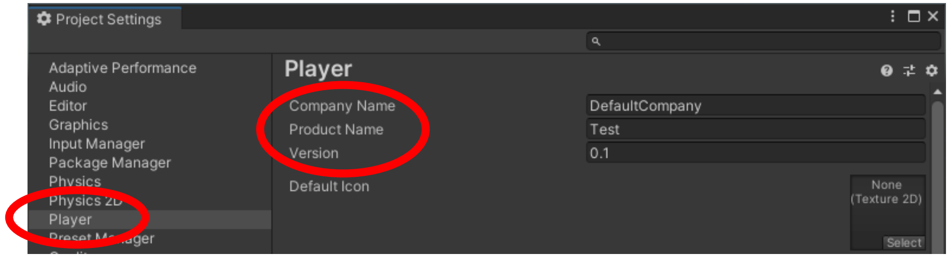

- In the same window, in the Other Settings tab, find the Minimum API Level and select Android 6.0 Marshmallow (API level 23). Leave the Target API Level as Automatic (highest installed).

- Still in the Other Settings tab, under Configuration, change the Scripting Backend to IL2CPP. Clear the ARMv7 checkbox and select ARM64.

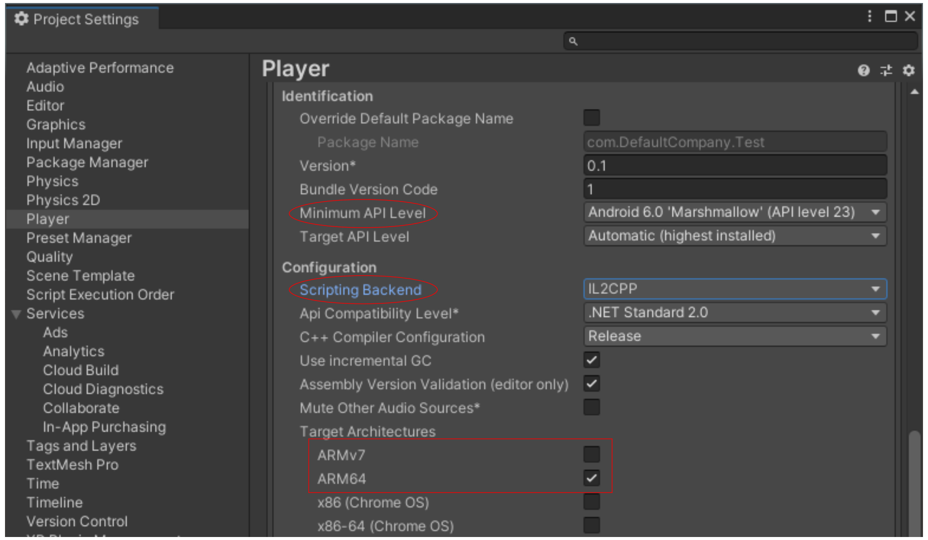

Configuring the Unity Project

- Go to Edit -> Project Settings -> XR Plugin Management and click Install XR Plugin Management.

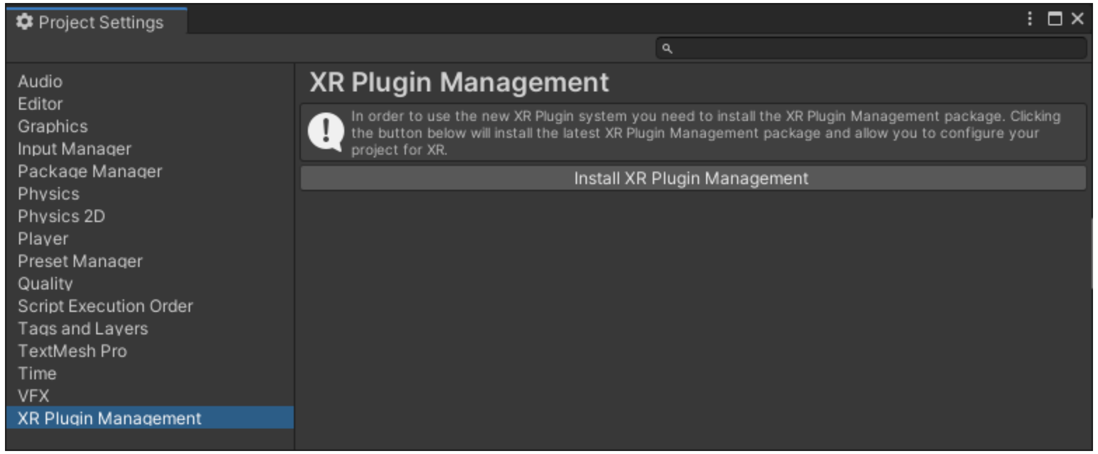

- After installing, in the Android tab, select Oculus.

- Click the PC tab (red circle on the image) and select Oculus.

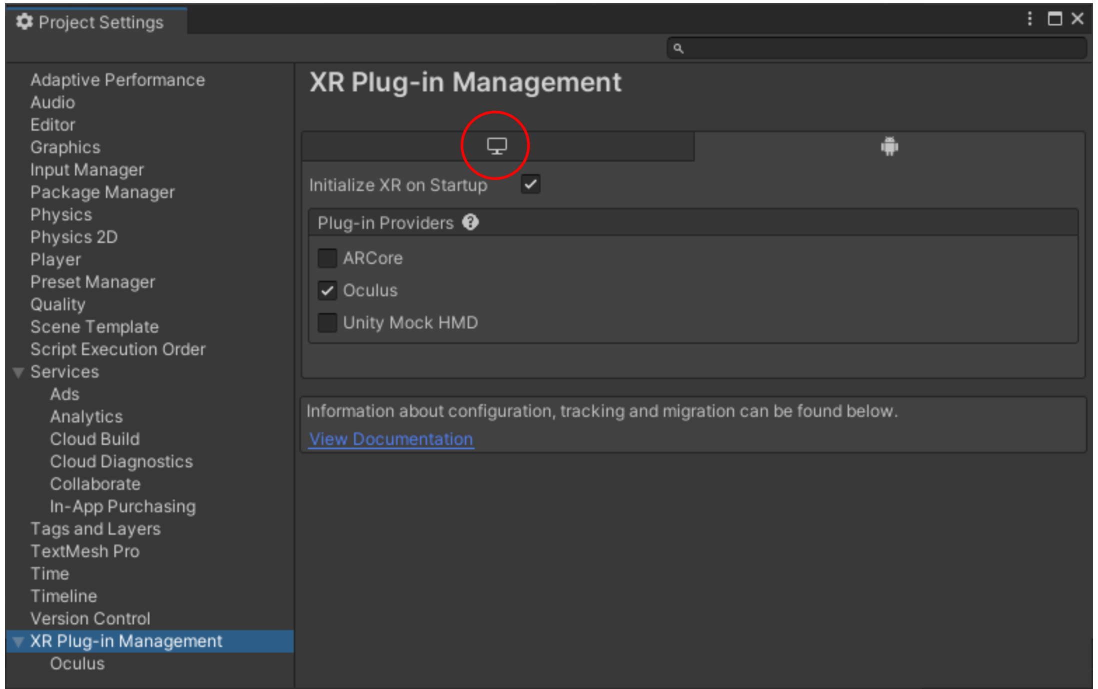

Configuring the Unity Project

- Go back to Edit -> Project Settings -> Player and then the Other Settings tab. Under the Rendering option, change the Color Space to Linear.

- Clear the Auto Graphics API checkbox and make sure OpenGLES3 is the first one in the Graphics API below.

- Make sure Multithreaded Rendering is selected.

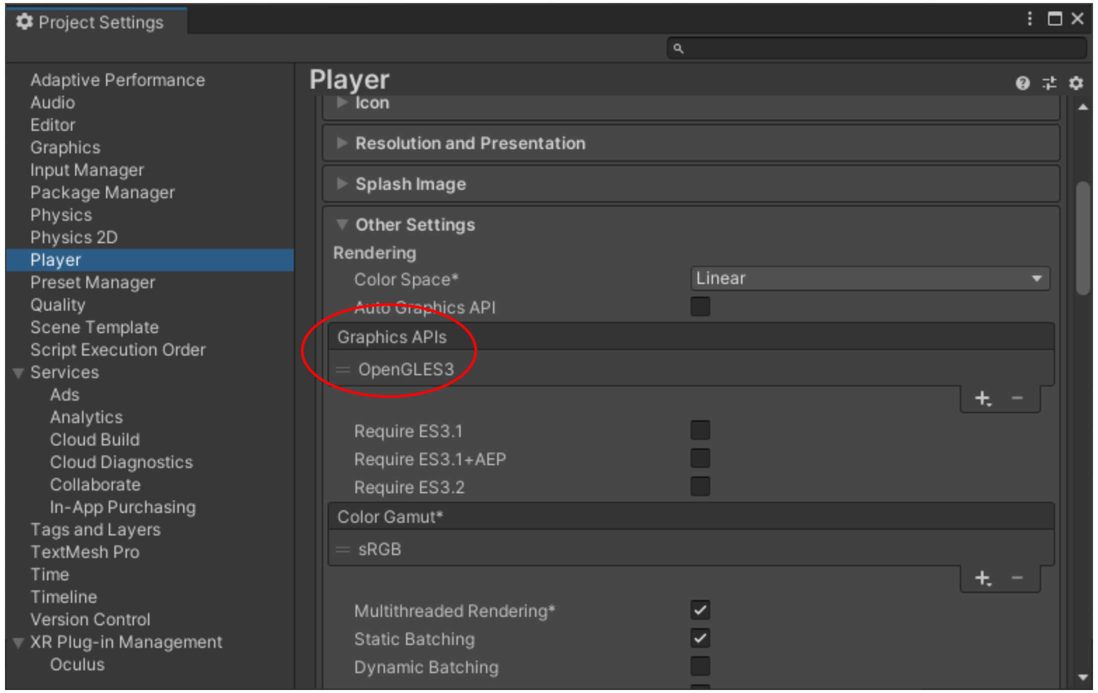

Configuring the Unity Project

- Go back to Edit -> Project Settings -> XR Plugin Management and click the submenu Oculus, on the left.
- Select the version of the Oculus Quest that will be used and check the Low Overhead Mode (GLES). Disable all other checkboxes.

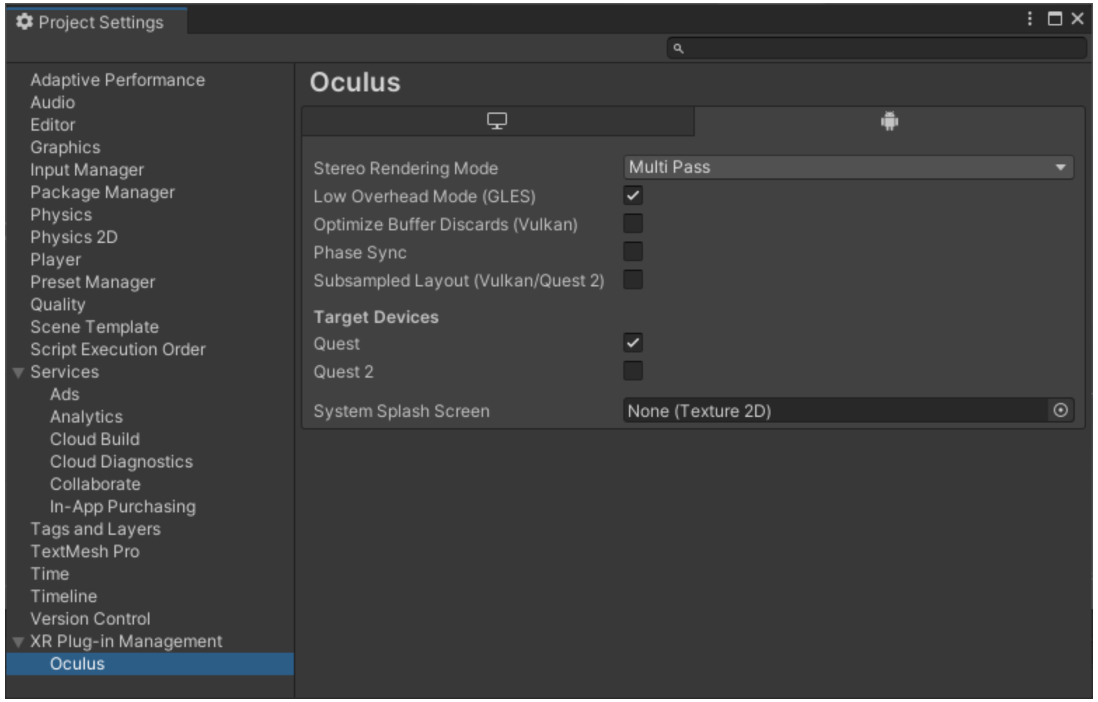

Configuring the Unity Project

- The last step is to configure the Quality of the project. Access Edit -> Project Settings -> Quality and click on Medium (should have a green checkmark under the Android icon).

- Set Pixel Light Count to 1.
- Set Texture Quality to Full Res.
- Set Anisotropic Textures to Per Texture.
- Set Anti Aliasing to 4x.
- Clear the Soft Particles check box.
- Select Realtime Reflections Probes.
- Select Billboards Face Camera.

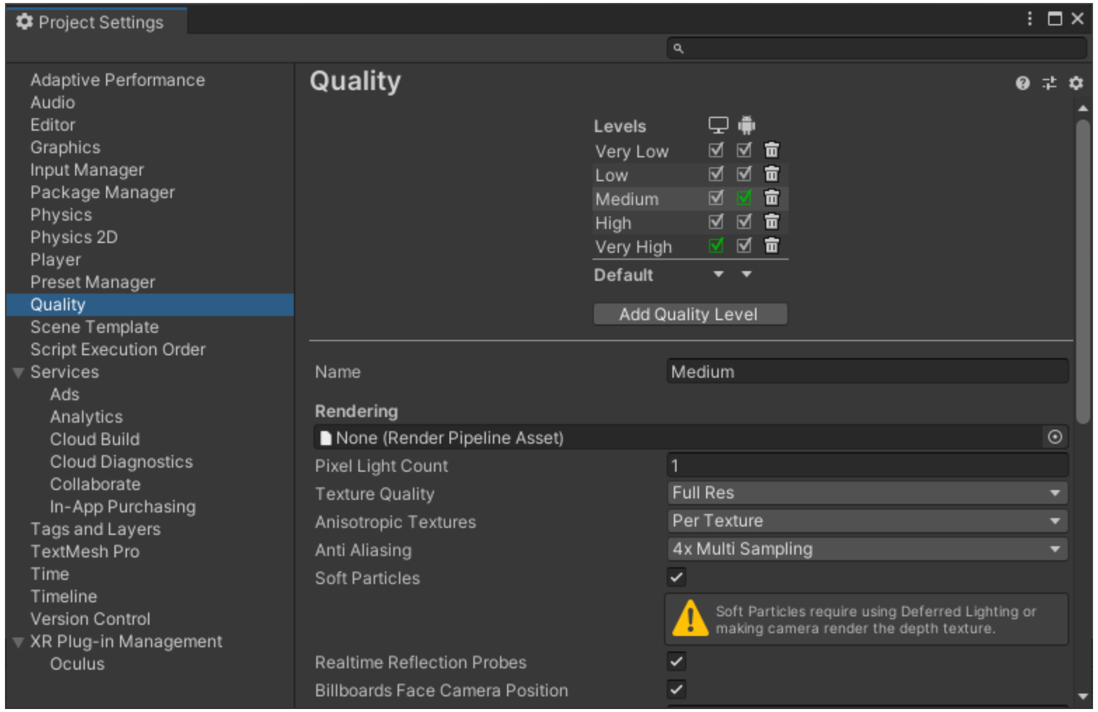

Importing the Unity Project Files

- With the project configured and the Oculus SDK installed, download the project files **(VRAppFinal.unitypackage)** in the link: https://github.com/AlysonSouza/FingerCountingVR
- Import the downloaded package using the menu Assets -> Import Package -> Custom Package.
  - Select the downloaded unitypackage file.
  - Import all assets.
- In the Project tab, navigate to the Hand Experiment folder, then Scenes and open the BaseRoomScene.

Importing the Unity Project Files

- In the Hierarchy tab, on the left side of the screen, expand the OVRCameraRig by clicking on the arrow next to it. Expand TrackingSpace, then expand LeftHandAnchor. Select OVRCustomHandPrefab_L. In the Inspector tab, on the right side of the screen, click “Auto Map Bones”. The list below should fill with finger names.

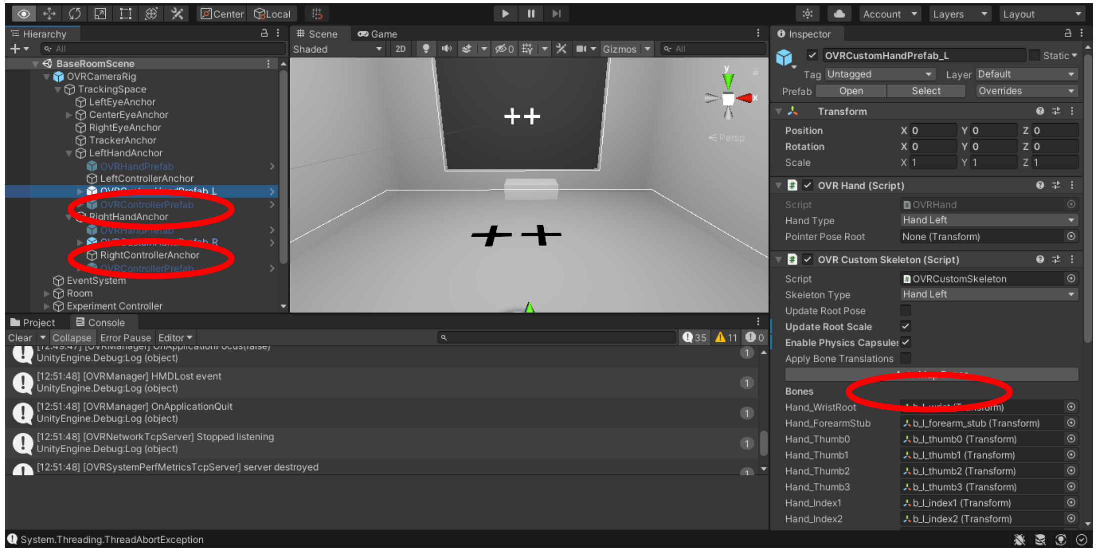

- Do the same to the OVRCustomHandPrefab_R, under RightHandAnchor.

- The application is now ready to be tested inside Unity.

Testing the Unity Project

- Press Play on the Unity Editor. The Game tab should show the blackboard seen from the floor (this happens because Unity is not detecting a VR Headset, so it positions the camera on the ground).
  - It is possible to click **Maximize on Play** in the Game tab before pressing play to have a full screen view.

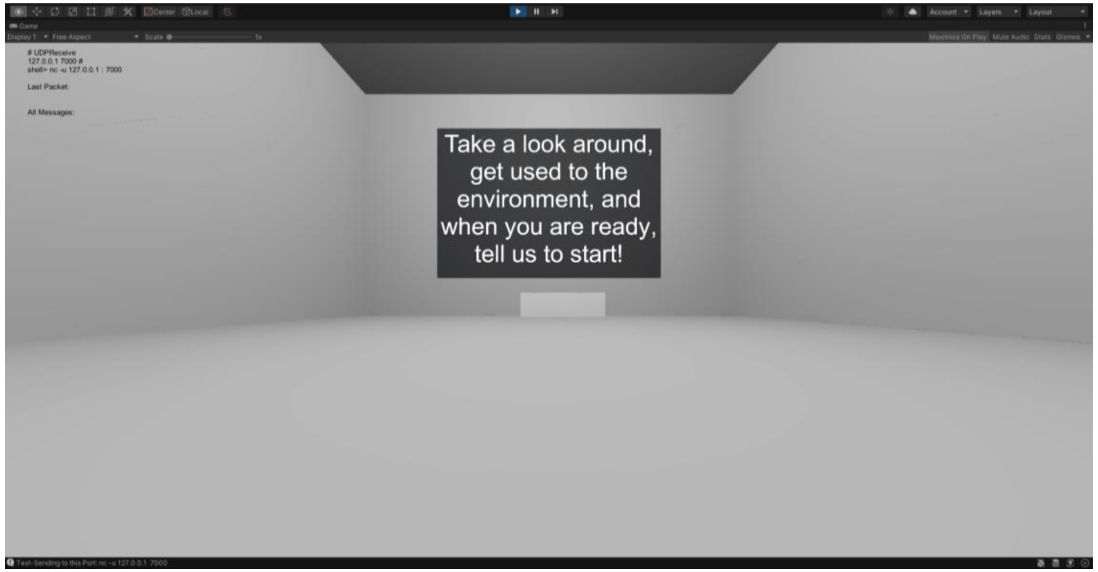

- There is some text on the top left corner of the screen to help debug the connection with the Experiment Controller in the next steps. This will not show in the VR headset.

Testing the Unity Project

- The application has some shortcuts to enable testing on the Unity Editor. Start the application for a test run by clicking inside the game window (represented in the image by the red rectangle) and pressing **S**.

- After seeing the message about the dominant hand, press **R** to simulate a right-handed individual or **L** to simulate a left-handed individual.

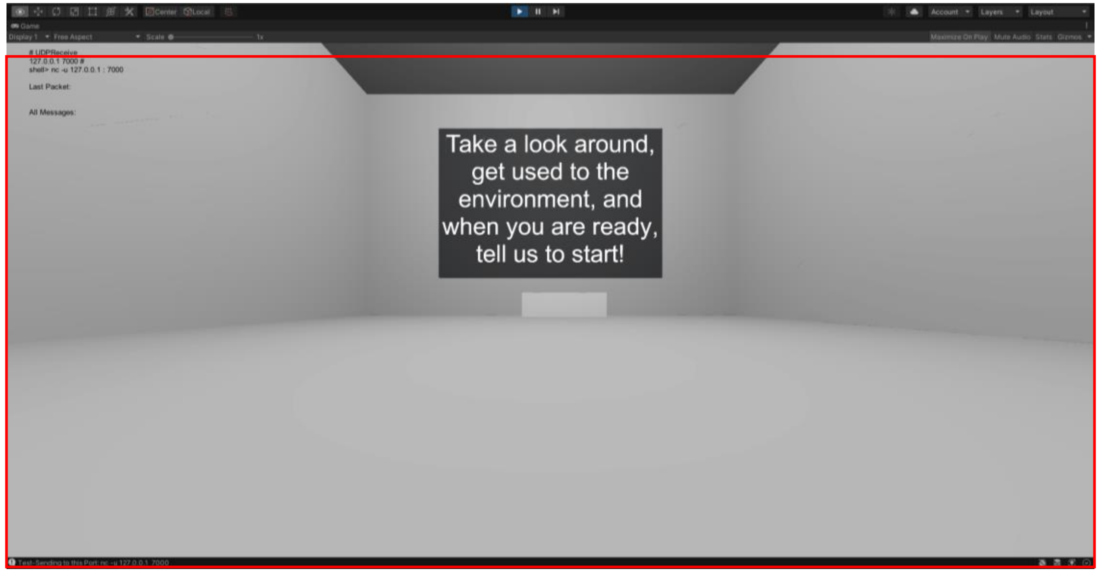

Testing the Unity Project

- Next, the blackboard will display a plus sign. This signals the participant to rest their hand on the ball or table.
- Press **A** to simulate that the participant has rested their hand. This will deliver the tactile stimulation and show a minus sign on the screen, showing the application is waiting for the participant’s gesture.
- Press **Z** to simulate that the gesture was made by the participant. This will trigger the visual stimulation part of the experiment.
- Press **C** to simulate that the participant has given their verbal response so the application moves to the next trial, going back to the +.

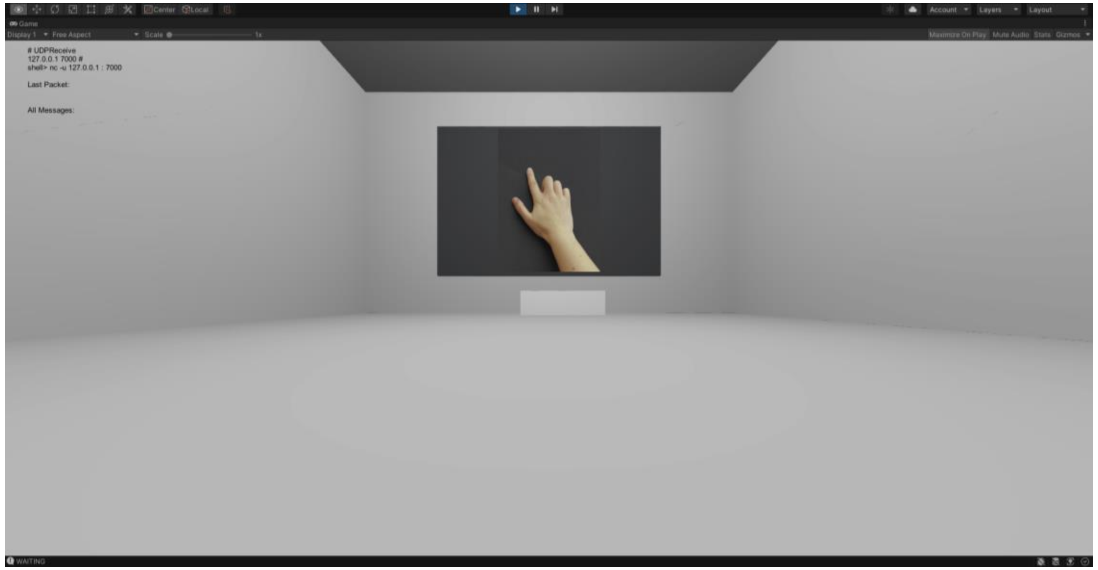

Testing the Unity Project

- After going trough all the trials (8, in the example), the experiment will be finished, displaying a “thank you” message.

- Note that the last three trials have no visual cue, as they are supposed to use the participant virtual hand as the visual cue, which depends on the Oculus Quest being connected to work.
  - In those trials, the numbers used to generate the fake virtual hand and the motor stimuli (explained later in this Supplementary Material) are instead shown on the blackboard (this will be disabled after testing).

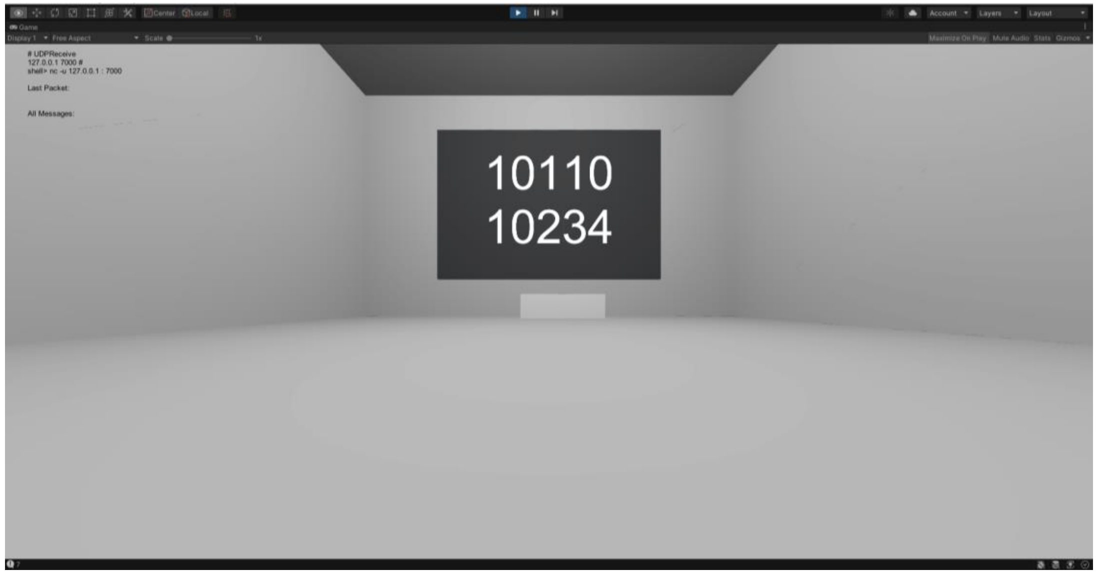

Testing the Unity Project

- After reaching the last screen, press Play again to stop the application.
- The next step is to test the network communication between the Experiment Controller and the VR application.
- Open two instances of Unity, one with the Experiment Controller and other with VR appliation. Connect the Arduino to the PC as described in Supplementary Material 1. Press Play on both instances.

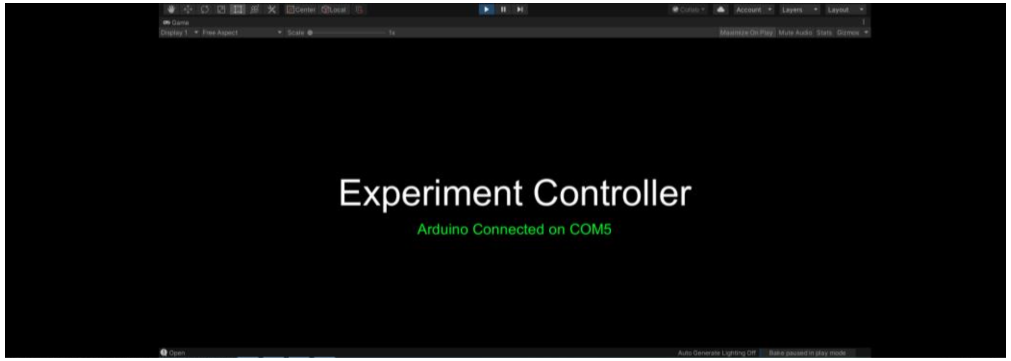

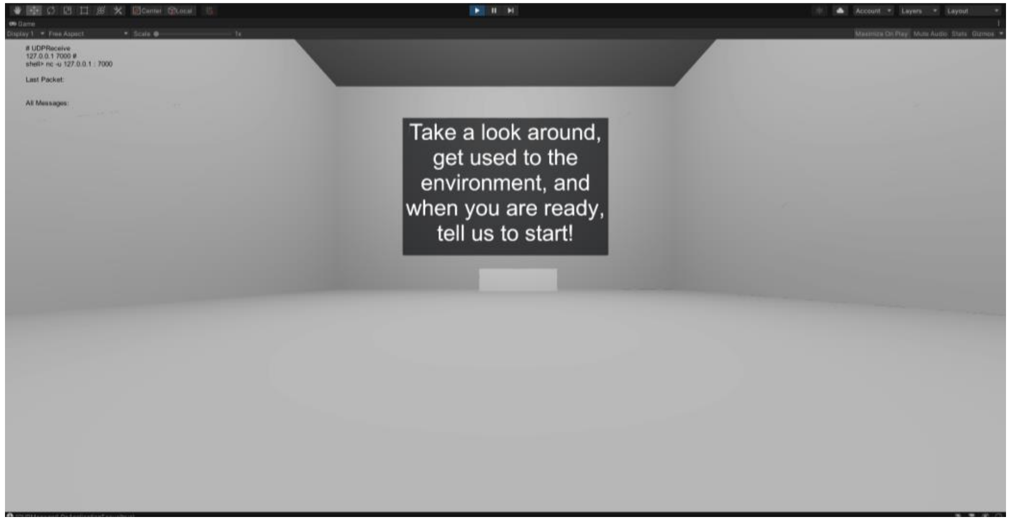

Testing Network Communication

- Be **very** mindful of which instance is active when you press the keyboard shortcuts. The keypress will only be identified by the Unity that is active when it happens.
- With both projects on Play mode, select the Experiment Controller and press **S**. This should send a signal to the VR application telling it to start.
- If everything worked correctly, a Start message will be displayed on the top left corner of the VR application (as shown on the image to the right) and the application will swap to the Dominant Hand part.

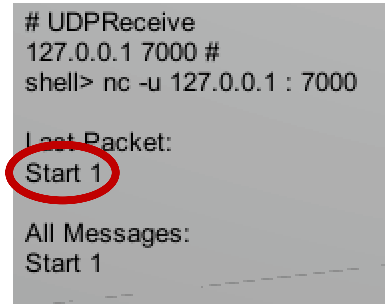

Testing Network Communication

- After once again selecting the Experiment Controller, press **R** for the right hand or **L** for the left hand. This will display another message in the VR application and will start it.

- To test the connection back, go to the VR Application (it should be displaying a + sign) and press **A**. This should trigger the first tactile stimulation request, vibrating the motors on the Arduino and lighting the LED, validating the other side of the communication (from the VR application to the Controller).

- To test all the trials, the **A**, **Z**, **C**, combination mentioned before can be used on the VR application (with its window selected) so it keeps sending stimulation requests to the Experiment Controller.

Troubleshooting Network Communication

- If no messages were displayed in the VR application, first make sure both are running and that the Experiment Controller was selected when pressing **S** to start.

- In the Hierarchy, click on the Network Controller in both applications. Check to see if both have 127.0.0.1 as the IP and the ports are matching correctly – the Send in one application must be the Receive in the other, as described in Supplementary Material 1, and vice-versa.

- If still no messages are seen, try to use different ports than those selected (5300 and 5301, for example). Remember to stop both applications before changing those values and then pressing Play again.

- If the messages are still blocked, try to contact the network admin about blocked UDP ports.

Preparing the VR Headset

- If this is the first use of the Oculus Quest VR headset, it needs to be configured using a mobile app. Download the Oculus Mobile App on an Android or iOS phone and follow the in-app instructions to sign.

- Pair the headset with the app following the instructions on screen and conclude the setup process through the Oculus Quest headset. Make sure to also update it to the latest version.

- The official website has a guide for setting up and updating the device: https://support.oculus.com/articles/getting-started/getting-started-with-quest-2/index-getting-started-quest-2

Preparing the VR Headset

- With the Oculus Quest already setup and signed in, enable the Developer Mode. To do so, open the Oculus mobile app, select the paired headset and turn on the Developer Mode.
- With a USB-C cable, connect the device to the PC (it is also possible to use a USB-C to USB cable). A popup should show on the device asking to allow data. Click Allow, in the device.
- In Unity, go to File -> Build Settings (Ctrl+Shift+B on Windows). On Run Device, the headset should now be showing as an option. Select it.

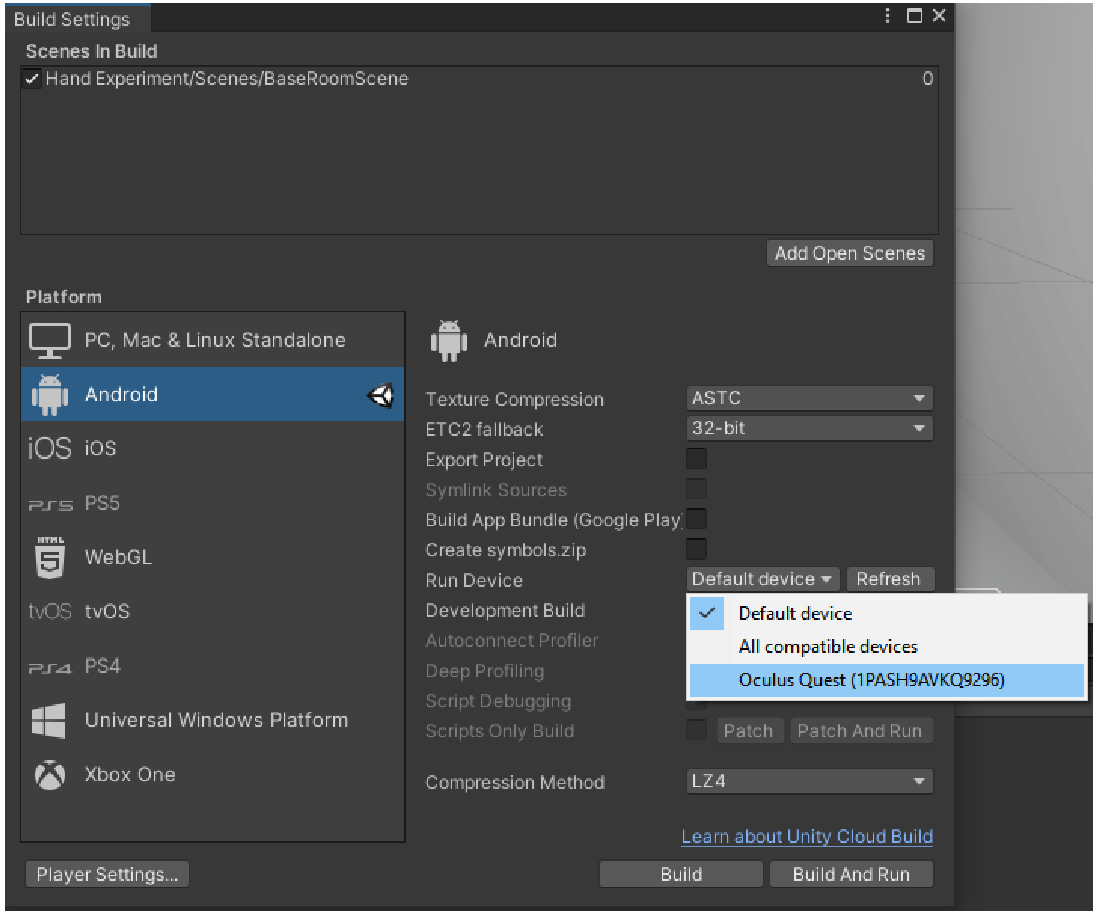

Preparing the Application for the VR Headset

- There are two different ways the application can be run on the VR headset. The easiest way is to use Oculus Link to simulate a tethered VR headset. This way, the application can be run from Unity directly to the headset, as if it was being run on the PC. This makes network communication more trivial and the application execution and testing easier. This will be described in the next steps.

- The second way is to compile the application as a .apk file and install it in the headset as a native application. This will require a network connection between the VR headset and the PC, the correct configuration of both devices IPs in the Network Controller component in both and a redeploy of the application file every time a change is made in the application code. The advantage is that the headset can be totally wireless, and the application can be started without the need for a PC, after installing, directly on the VR headset.
  - To run using this method, first configure both Network Controllers with correct IPs and ports as mentioned in the Supplementary Material 1, then select the Run Device as mentioned before and click Build and Run. Select the APK name and Unity should install it in the device.

Using the Oculus Link to Run the Application in Unity

- For the Oculus to work directly inside Unity, the Oculus Link PC app is needed. Unplug the headset then download and install the software on the PC from the official website: https://www.oculus.com/setup/

- After installing the software, plug the headset back to the PC and you may be prompted with three popups:
  - **DENY** data access, this time.
  - **ALLOW** USB debugging from the PC.
  - **ENABLE** the Oculus Link.

- You may unplug the headset and plug it again if a different option is selected.
  - The official page has some extra troubleshooting if needed: https://support.oculus.com/articles/headsets-and-accessories/oculus-link/connect-link-with-quest-2/

Using the Oculus Link to Run the Application in Unity

- In the VR headset, Go to Quick Settings and select Oculus Link. The VR headset should now be ready to run directly from the Unity Editor.

- When ready to test, connect the Tactile Device to the PC and the motors to the user's fingers, run the Experiment Controller and press play. Afterwards, run another Unity instance with the VR Application and press Play. The VR Application should now be displaying in the VR headset.

- Raise both hands in front of the headset to detect them. Afterwards, select the Experiment Controller and press **S** to start the application and then press **R** or **L** to select the dominant hand.

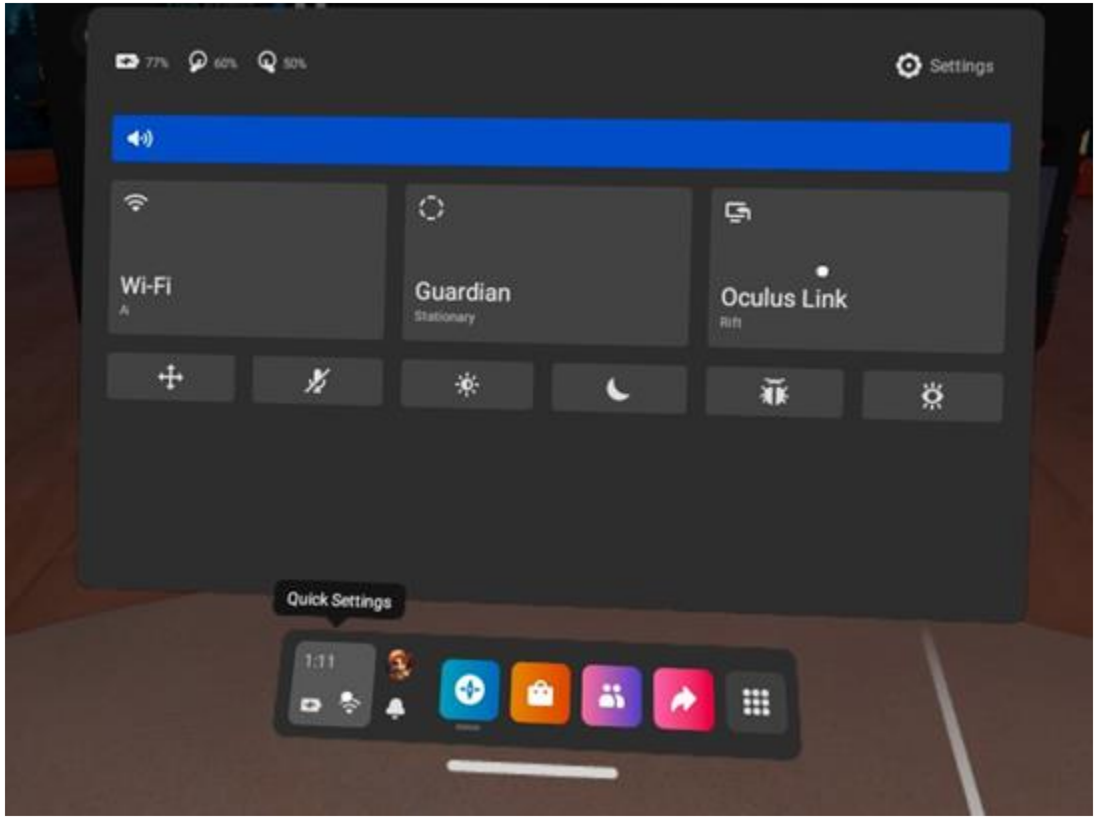

Using the Oculus Link to Run the Application in Unity

- Rest (curl) the selected dominant hand to generate a tactile stimulation.
- Make the gesture according to the tactile stimuli to trigger the visual stimuli.
- Say the number on the screen to activate the voice threshold and enable the answer input.
- The application will wait for the input of the answer. Select the Experiment Controller on the PC and press 1-6 on the keyboard to record the number as the answer – 6 should represent any not possible answer.
- Rest the hand again for the next stimuli. This is the cycle for a single trial.
  - The sample file contains 8 trials.

- Now that the application is working, the next step is to customize it.

Configuring the Voice Detector Threshold

- If the application is to be used in a noisy environment or on a quiet one, the voice detection can vary. If needed, it is possible to change the detectable threshold by going to the Voice Detector component in the Hierarchy and changing the value for the threshold. Test the application again to find one that is good enough.

- To do so, press play with the Voice Detector component selected. It will display the values of noise it is detecting in the Test Sound field. Change the threshold to one that only triggers when something is said close to the mic. Change this after **STOPPING** the application, as changes in the play mode are automatically reverted afterwards.

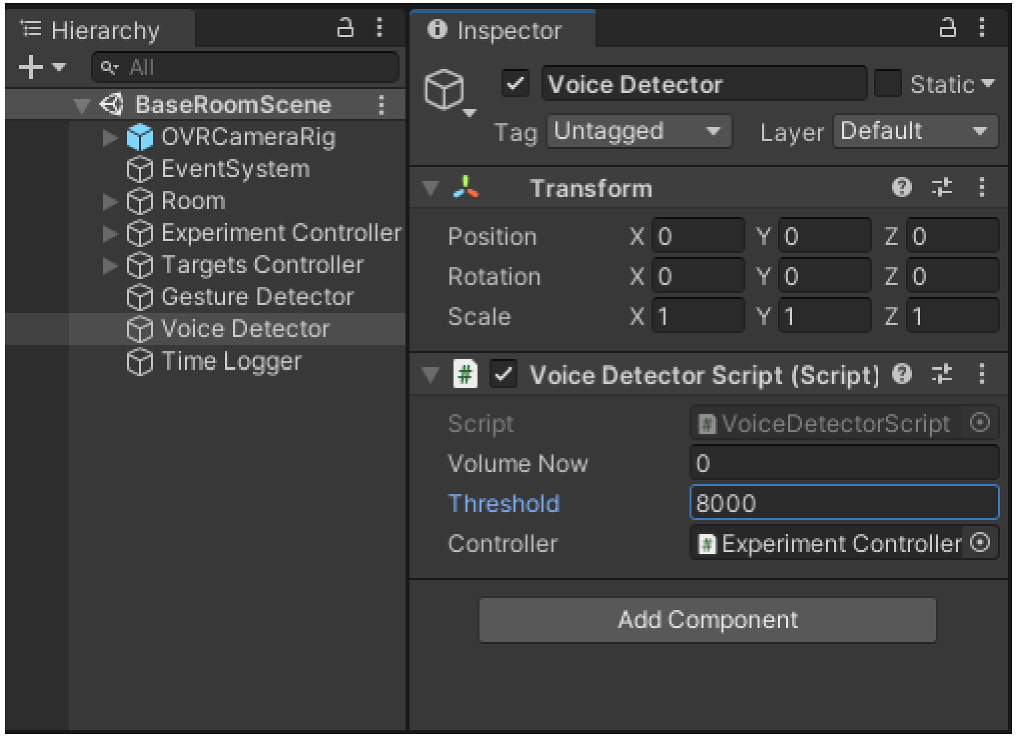

Configuring the Trials List

- All the trials are directly imported from a trial file in the Assets -> Resources folder. Customizing this trials file is the easiest way to change the application.

- The trial file, shown in the image, is a .csv file containing 5 elements for each trial – the trial ID, the motor stimulus, the format of the visual stimulus, the visual stimulus to display and the canonicity of the visual stimulus. The software uses this file to determine which image to show on the black board and how to deliver the tactile stimulation.

Trial ID	Motor Stimulation	Visual Stimulation Format	Visual stimulation	Canonical or Non-Canonical
1	11000	Picture	2	C
2	11110	Picture	3	NC
3	11100	3D Model	2	C
4	10001	3D Model	3	NC
5	11101	Arabic Digit	4	NA
6	11111	VR Hand	12345	C
7	10110	VR Hand	10234	NC
8	10011	VR Hand	13425	NC

Configuring the Trials List

- Trial ID is a number identifier for each trial.
- Motor Stimuli tells the Tactile Stimulation Device which fingers to vibrate (1 being on and 0 being off, from thumb to pinky).
- The Visual Stimulation Format must be one of the following:
  - **Picture** – Shows an image of the hand on the blackboard.
  - **3D Model** – Shows a 3D model of the hand on the blackboard.
  - **Arabic Digit** – Shows a single digit on the blackboard.
  - **VR Hand** – Enables the finger swapping and shows the virtual copy of the user's hand.

	A	B	C	D	E
1	Trial ID	Motor Stimulation	Visual Stimulation Format	Visual stimulation	Canonical or Non-Canonical
2	1	11000	Picture	2	C
3	2	11110	Picture	3	NC
4	3	11100	3D Model	2	C
5	4	10001	3D Model	3	NC
6	5	11101	Arabic Digit	4	NA
7	6	11111	VR Hand	12345	C
8	7	10110	VR Hand	10234	NC
9	8	10011	VR Hand	13425	NC

Configuring the Trials List

- Visual Stimulation is the number to show on the blackboard, using the format in the previous column. It can be 2, 3 or 4 for **Picture**, **3D Model**, and **Arabic Digit** formats and must be a hand configuration (described later) for the **VR Hand** condition.

	A	B	C	D	E
1	Trial ID	Motor Stimulation	Visual Stimulation Format	Visual stimulation	Canonical or Non-Canonical
2	1	11000	Picture	2	C
3	2	11110	Picture	3	NC
4	3	11100	3D Model	2	C
5	4	10001	3D Model	3	NC
6	5	11101	Arabic Digit	4	NA
7	6	11111	VR Hand	12345	C
8	7	10110	VR Hand	10234	NC
9	8	10011	VR Hand	13425	NC

- Canonical or Non-Canonical describes if the pose to be displayed, when relevant, is the canonical or non-canonical one. This column accepts C, NC or NA (when not applicable).

- For example, if the format is **Picture**, the visual stimulation is 2 and the canonical is C, the image on the right will be displayed.

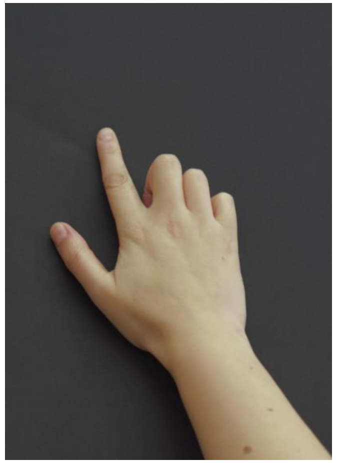

Configuring the Trials List

- The **VR Hand** stimulation format enables the finger swapping in the virtual hand, as described in the paper. This swap occurs according to the values in the Visual Stimulation field.

- The field takes 5 numbers, representing the virtual fingers from thumb to pinky. The first number will describe which real finger controls the virtual thumb, the second number which real finger controls the index, and so on. Each number ranges from 0 to 5, with 1 being the thumb and 5 the pinky and 0 being used to make the virtual finger immobile. **DO NOT** use spaces between the numbers.

- For example, the text “12345” makes the virtual hand behave as the real one, as no fingers are swapped. The text “00000” makes the virtual hand have no fingers moving, although the palm and wrist would still move.

Configuring the Trials List

- As described in the paper, the thumb should only be mapped to itself or be left immobile. Therefore, the first number must always be 0 or 1.

- For the other 4 numbers, they can vary from 0 to 5 (again, 1 is not recommended, as the virtual finger will bend to be oriented as the thumb). 0 makes the corresponding virtual finger immobile, 2 makes the corresponding finger move according to the real **index** finger, 3 makes the corresponding finger move according to the real **middle** finger, and so on.

Configuring the Trials List

- Looking at trial 7, for example, the tactile device will stimulate the thumb, middle and ring finger (10110).

- Afterwards, when the visual stimulation happens, the virtual hand will have the virtual thumb mapped to the real one (1), the virtual index finger not moving (0), the virtual middle finger being controlled by the real index (2), the virtual ring finger being controlled by the real middle finger (3) and the virtual pinky being controlled by the real ring finger (4), creating the visual stimulation text **10234**. A comparison between the real hand and the virtual one, in this case, can be seen in the images to the right.

	A	B	C	D	E
1	Trial ID	Motor Stimulation	Visual Stimulation Format	Visual stimulation	Canonical or Non-Canonical
2	1	11000	Picture	2	C
3	2	11110	Picture	3	NC
4	3	11100	3D Model	2	C
5	4	10001	3D Model	3	NC
6	5	11101	Arabic Digit	4	NA
7	6	11111	VR Hand	12345	C
8	7	10110	VR Hand	10234	NC
9	8	10011	VR Hand	13425	NC

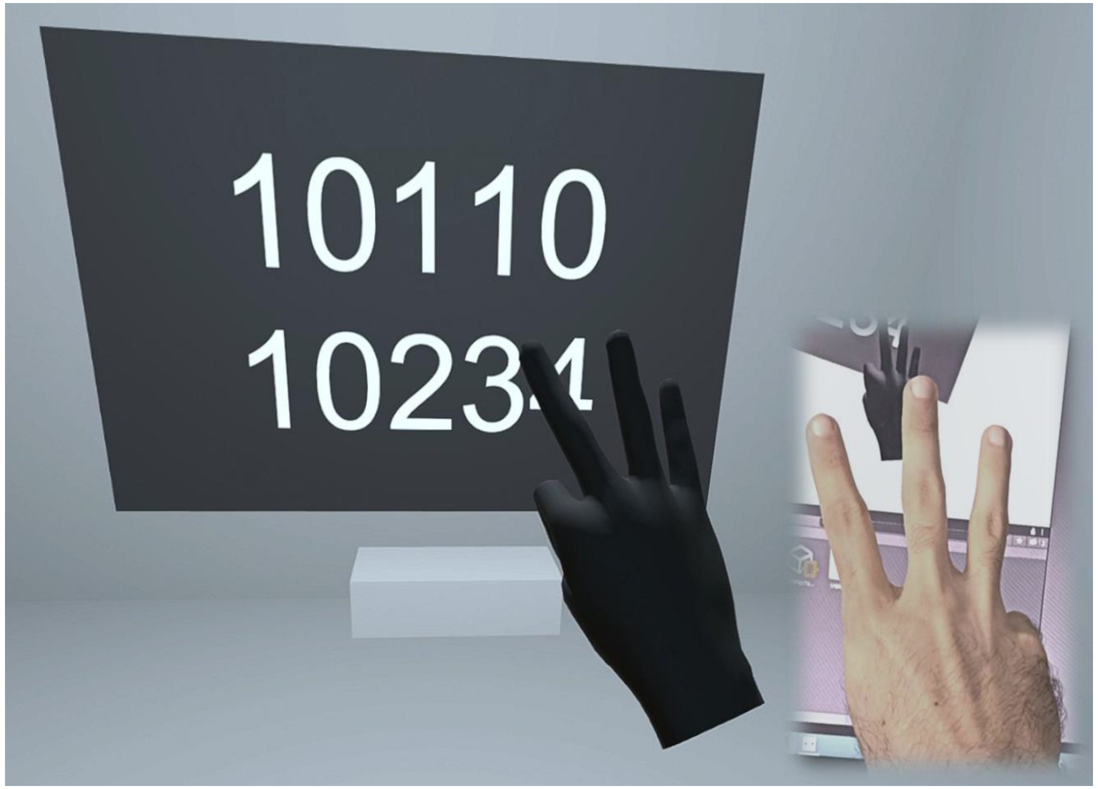

Configuring the Trials List

- When editing the trials file, do not change its name or format (trialsList.csv). New trials can be added or removed from the list, but the table structure must be kept the same. The possible values for each column must be respected. The VR Application will bring up an error on startup if any line is detected to be wrong.

- If using a text editor to edit the file, be careful not to add spaces or remove the semicolons that are in the file. If using an app such as Microsoft Excel, be careful with the cell format as it may delete zeroes on the left of the number, making it have less than 5 digits.

Removing the Test Tag

- When done testing and ready to run the application, select the Experiment Controller in the Hierarchy tab and uncheck the Test checkbox in the Inspector.

- This will disable the numbers in the screen in the **VR Hand** visual format and disable the key presses on the VR Application, allowing the Experiment Controller to take over.

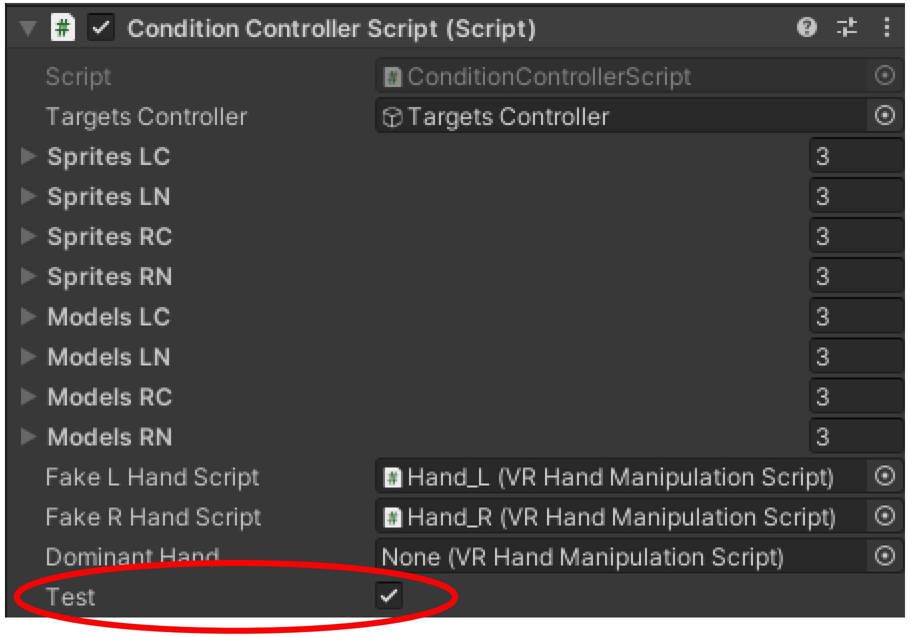

Customizing the Application

- The application can be fully modified, as the source code is all made available. Other modifications are out of the scope of this supplementary material, but feel free to add new images, new trials or new poses to the models presented in the application. The ones contained are the ones used in the first experiment using the method.

- The images are in the folder Assets -> Hand Experiment -> Images and respect the naming format of the canonicity plus the trial with an L indicating if it is the left hand – C2, C2L, and so on.

- The models are defined in Unity directly, in the Hierarchy, Targets Controller -> Hands.
  - The Base Hand component can be activated with the application in Play Mode and the numbers in the keyboard can be used to control individual fingers and generate new patterns.

Retrieving Application Data

- After each experiment is started, a new file with the results of that experiment is created in the application folder. The default folder can be found at:
  - C:\Users**YOUR-USER**\AppData\LocalLow**YOUR-COMPANY****YOUR-PROJECT**

- The files are created with a sequential number, each time the application is executed. To restart from zero, simply delete or move the files from the folder. The application looks for the first available number.

- The file registers:
  - Participant ID – Same as file name
  - Start Time – Date and Time
  - Load Time – Time to load application in milliseconds (ms)
  - Dominant Hand
  - Trial ID | Motor Stimulus Time | Motor Stimulus | Gesture Detection Time | Visual Stimulus Time | Visual Stimulus | Canonical | Voice Detection Time | Answer

  - All times are recorded in milliseconds since the application started and should be used relative to each other.

Virtual Reality System

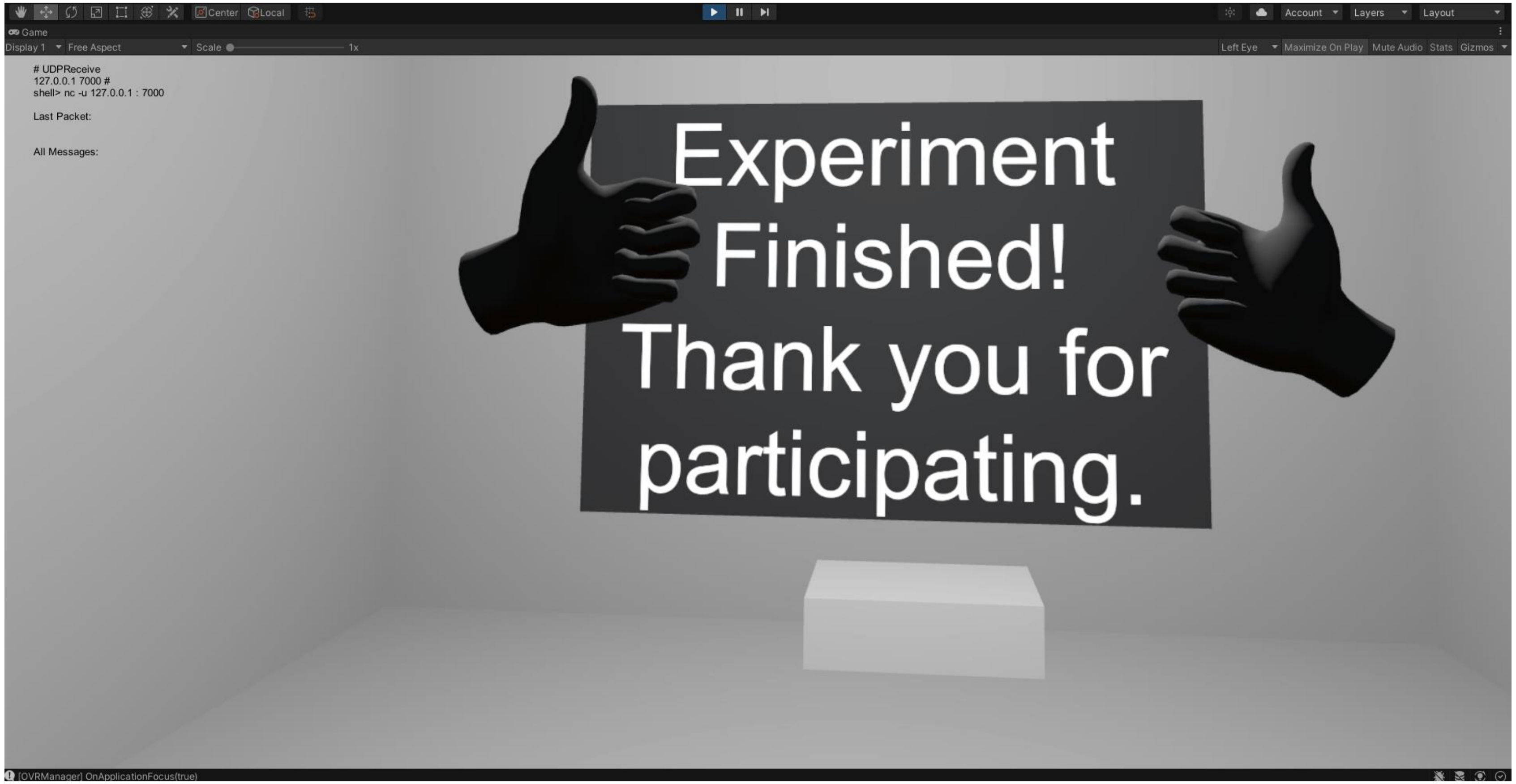

Supplement: Supplementary file 2 [file Presentation_2.pdf]
